# Epithelial Dynamics of Cystogenesis in Genetic Models of Autosomal Dominant Polycystic Kidney Disease

**DOI:** 10.3390/cells15030297

**Published:** 2026-02-04

**Authors:** Mengyan Sun, Zhaohui Wu, Mingqiang Hu, Wei Luo, Xiaole Chen, Ming Ma

**Affiliations:** Institute of Developmental Biology and Regenerative Medicine, Southwest University, Beibei, Chongqing 400715, China; smy690218@email.swu.edu.cn (M.S.); wuzhaohui0711@163.com (Z.W.); hmq21297@163.com (M.H.); kk0701@email.swu.edu.cn (W.L.); cxl24362025@163.com (X.C.)

**Keywords:** ADPKD, cyst, clonal expansion, epithelial dynamics, mathematical modeling

## Abstract

Autosomal dominant polycystic kidney disease (ADPKD), caused by mutations in *PKD1* or *PKD2*, is characterized by progressive and exponential enlargement of renal and hepatic cysts. However, the epithelial dynamics that generate this growth pattern remain incompletely understood. Using *Brainbow/Confetti* multicolor clonal lineage tracing in developmental and adult-onset ADPKD mouse models, we show that polycystin-deficient epithelial cells initiate clonal expansion at early stages of tubule dilation and continue to expand throughout cyst progression. Concurrently, cyst-lining cells undergo a progressive transition from columnar to flattened morphology, which amplifies luminal enlargement independent of cell number. Integrating these measures, we developed a mathematical model demonstrating that the combination of this clonal expansion and epithelial cell shape remodeling is sufficient to produce the exponential growth trajectory observed in ADPKD. Together, these findings define the core epithelial mechanisms that drive cyst initiation and expansion, and may provide a mathematical framework for the emergent exponential growth of cysts.

## 1. Introduction

Autosomal dominant polycystic kidney disease (ADPKD) is one of the most common monogenic diseases, with an occurrence of 1:1000 to 1:500. Cysts start to develop in the kidneys and liver from adulthood and cysts increase in size and number. Half of patients progress to end stage renal disease by age 60, requiring dialysis or renal transplantation.

ADPKD is caused by mutations on *PKD1* and *PKD2*, which encode polycystin 1 (PC1) and polycystin 2 (PC2), respectively [1,2]. Although inherited in a dominant manner, cystogenesis follows a cellular recessive model, where a somatic “second hit” mutation inactivates the remaining functional allele [3,4,5,6]. This loss of PC1 or PC2 function in individual renal and biliary epithelial cells is the established trigger for cyst initiation. “Second hit” events happen stochastically in renal and biliary epithelial cells throughout life; therefore, cysts increase in size and number continuously [3,4,5]. Mouse models with conditional inactivation of the ortholog *Pkd1* or *Pkd2* genes recapitulate the human disease, with the timing of cyst onset and progression dependent on the induction stage, developmental inactivation causing rapid cystogenesis and adult-onset inactivation yielding a slower, more clinically relevant course [7,8].

PC1/2 form a complex and localize to primary cilia, a cell antenna that detects stimuli and transmits to the inside of cells [9,10,11]. Ablation of cilia suppresses cyst progression, suggesting that cilia harbor signal to drive cyst progression termed cilia dependent cyst activation (CDCA) [12]. *Glis2*, a transcription factor, whose expression was found to correlate with cilia dependent manner, and its inactivation slows cyst progression [13]. The growth of cysts relies on a series of signaling pathways including cAMP, mTOR signaling [14,15], cellular metabolism [16,17], extra-cellular matrix interactions with Integrins [18], transcription factors [13,19], epigenetic factors [20,21] etc., while the precise mechanistic links between the loss of polycystin and the activation of these signaling networks remain incompletely defined.

Cyst growth dynamics in ADPKD have been characterized using multiple approaches in both human patients and murine genetic models. Segmented computed tomography (CT) and magnetic resonance imaging (MRI) of ADPKD patient kidneys have revealed key features of cyst development, including that the initial number of cysts predicts the rate of subsequent kidney enlargement [22]. Longitudinal MRI analyses tracking total kidney and cyst volumes over three years demonstrated that cysts expand following an exponential growth pattern [23], consistent with the “snowball effect” model of cystic progression [24,25]. At the cellular level, insights from animal models have revealed that cyst progression is associated with several events, including low level proliferation [12,26], low level apoptosis [27], and a marked change in epithelial cell shape from columnar to flattened [27,28]. Interventions that slow cyst growth often correlate with reduced proliferation, underscoring its importance [29,30].

A major unresolved question in polycystic kidney disease concerns the in vivo dynamics of renal epithelial cells during various stages of cyst formation. Ideally, time-lapse imaging could reveal how tubular structures undergo morphogenetic transformation into cysts. However, cystogenesis is exceedingly slow. In humans, cysts often take years to decades to form and enlarge [31], while in adult onset murine genetic models, cyst development occurs over weeks to months [6,7]. Even in developmental models such as *Cdh16^Cre^*; *Pkd1^fl/fl^*, in which *Pkd1* is inactivated in the distal nephron from embryonic stage, cysts still require days to enlarge [26]. The invasive surgical procedures and prolonged kidney exposure required for in vivo imaging render such experiments impractical. To overcome these challenges, lineage tracing provides an alternative means of assessing epithelial cell behavior during cystogenesis. In particular, *Brainbow/Confetti* multi-color clonal analysis systems, which label cells with distinct fluorophores, enable long-term visualization of cell progeny within the same tissue [32].

To track the renal epithelial cell behavior and their contribution to cyst formation, we employed the *Rosa^Brainbow^* mice for multicolor clonal analysis. This approach was combined with inducible *CAG^CreER^*; *Pkd1^fl/fl^* and *CAG^CreER^*; *Pkd2^fl/fl^* models, in which low-dose tamoxifen induced sparse labeling of renal cells with multiple fluorophores while simultaneously inactivating *Pkd1* or *Pkd2*. In the liver, we used *CK19^CreER^*, a cholangiocyte specific inducible transgenic line [33]. We analyzed the dynamics of cell shape during cyst formation and developed a mathematical model coupling cell expansion with morphological change, revealing that cyst growth occurs exponentially. Together, our results delineate the epithelial dynamics underlying cystogenesis, showing that the expansion of cyst-lining cells, coupled with coordinated cell shape changes, drives exponential cyst growth.

## 2. Materials and Methods

### 2.1. Generating Pkd1 and Pkd2 Conditional Mice, and Mice Husbandry

The *Pkd1* conditional allele was generated by Cyagen (Guangzhou, China) using homologous recombination in C57BL/6 embryonic stem cells. To engineer the targeting vector, homologous arms and the conditional knockout region were generated by PCR using BAC clone RP24-282C1 and RP23-46N5 from the C57BL/6 library as template. *LoxP* sites were inserted into intron 11 and intron 14 flanking exons 12–14, enabling Cre-mediated excision and generation of a null allele. The targeting vector contained a *Neomycin* resistance cassette flanked by *SDA* (self-deletion anchor) sites, and diphtheria toxin A (DTA) was used for negative selection. The targeting construct was linearized by restriction digestion with NotI, purified by phenol/chloroform extraction and ethanol precipitation, and transfected into C57BL/6 ES cells. ES cells were selected with G418 (200 μg/mL) beginning 24 h after electroporation. Ninety-three G418-resistant clones were picked and expanded in 96-well plates. Duplicate plates were generated, with one plate frozen at −80 °C and the other used for DNA isolation and PCR screening for homologous recombination. PCR screening identified 20 candidate targeted clones, and five were expanded and validated by Southern blot analysis. Correctly targeted ES clones were injected into blastocysts and transmitted through the germline to establish *Pkd1^fl^* mice. Genotyping of the *Pkd1^fl^* allele was performed using the primer pairs *Pkd1-F1/Pkd1-R1* and *Pkd1-F2/Pkd1-R2* (Table S2). Recombination of the *Pkd1* allele was assessed using genomic DNA isolated from kidneys of control (*Pkd1^fl/^*^+^) and tamoxifen-induced *CAG^CreER^*; *Pkd1^fl/fl^* mice with the primer pair *Pkd1-F1/Pkd1-R2* (Table S2).

*Pkd2^fl^* mice was generated by Cyagen using CRISPR/CAS9-mediated genome editing to insert *loxP* sites flanking exons 4–5, enabling Cre-mediated deletion. Conditional knockout founders were generated by co-injecting gRNA, a donor vector containing *loxP* sites, and Cas9 mRNA into fertilized mouse eggs. F0 founders were identified by PCR and sequence analysis and bred to the F1 generation to establish germline transmission. The gRNA sequences targeting *Pkd2* are provided in Table S2. Genotyping of the *Pkd2^fl^* allele was performed using the primer pairs *Pkd2-F1/Pkd2-R1* and *Pkd2-F2/Pkd2-R2*. Recombination of the *Pkd2* allele was assessed using genomic DNA isolated from kidneys of control (*Pkd2^fl/^*^+^) and tamoxifen-induced *CAG^CreER^*; *Pkd2^fl/fl^* mice with the primer pair *Pkd2-F1/Pkd2-R2*.

The following mouse strains were obtained from The Jackson Laboratory (Bar Harbor, ME, USA): *Ift88^fl^* [34] (JAX Strain # 022409), *CAG^CreER^* (ref. [35]) (JAX Strain # 004682), *CK19^CreER^* (ref. [33]) (JAX Strain # 026925), *Rosa^Brainbow^* (ref. [32]) (JAX Strain # 017492) have been described previously. The mice were ordered from Jax. All animals were housed in a specific pathogen-free facility under a 12 h light/dark cycle. Mice of the C57BL/6J strain were provided standard chow and water without restriction throughout the study.

### 2.2. Genotyping

Mouse tail samples were incubated at 55 °C overnight in lysis buffer containing 1 M Tris-HCl (pH 8.0), 0.5 M EDTA, 3 M KCl, 10% Tween 20, and 0.1 mg/mL proteinase K. Proteinase K was inactivated at 95 °C for 15–20 min, followed by centrifugation at 14,000 rpm for 10 min. PCR amplification was performed using specific primer pairs for the following genes: *Pkd1^fl^*, *Pkd2^fl^*, *CAG^CreER^*, *Ck19^CreER^*, and *Ift88^fl^* (Table S2). The PCR conditions were as follows: initial denaturation at 98 °C for 3 min; 30 cycles of 98 °C for 10 s, 60 °C for 10 s, and 72 °C for 10 s; and a final extension at 72 °C for 2 min. For *Rosa^Brainbow^* genotyping, PCR was performed using specific primers under the following conditions: initial denaturation at 98 °C for 3 min; 30 cycles of 98 °C for 10 s, 55 °C for 10 s, and 72 °C for 10 s; and a final extension at 72 °C for 2 min.

### 2.3. Statistics

Data were analyzed by one-way ANOVA followed by Tukey’s multiple-comparison test using GraphPad Prism 10 software. Comparisons between the two groups were performed using an unpaired two-tailed *t*-test. *p* < 0.05 was considered statistically significant. All data are presented as mean ± SEM.

### 2.4. Mouse Treatment

Tamoxifen (Macklin, Zhangjiang, Shanghai, China) was dissolved in corn oil (Aladdin, Fengxian, Shanghai, China) to a final concentration of 20 mg/mL. A single intraperitoneal injection of tamoxifen (100 mg/kg) was administered to *CAG^CreER^*; *Rosa^Brainbow/^*^+^, *CAG^CreER^*; *Pkd1^fl/fl^*; *Rosa^Brainbow/^*^+^, and *CAG^CreER^*; *Pkd2^fl/fl^*; *Rosa^Brainbow/^*^+^ at *P*_10_ or *P*_28_, while *CAG^CreER^*; *Ift88^fl/fl^*; *Rosa^Brainbow/^*^+^, *CAG^CreER^*; *Ift88^fl/fl^*; *Pkd1^fl/fl^*; *Rosa^Brainbow/^*^+^, *Ck19^CreER^*; *Pkd1^fl/^*^+^; *Rosa^Brainbow/^*^+^, and *Ck19^CreER^*; *Pkd1^fl/fl^*; *Rosa^Brainbow/^*^+^ mice were injected at *P*_28_.

### 2.5. Imaging

Mouse kidneys and livers were fixed overnight at 4 °C in 4% paraformaldehyde (PFA) and then immersed in 30% sucrose solution until the tissues sank. Samples were embedded in Tissue-Tek O.C.T. compound (Sakura Finetek, Torrance, CA, USA) and sectioned at 50 μm thickness using a cryostat. Confocal images were acquired using a Zeiss LSM 880 laser scanning confocal microscope (Microscopy GmbH, Oberkochen, Germany). Excitation wavelengths were 458 nm (mCFP), 488 nm (nuclear GFP), 514 nm (EYFP), and 561 nm (RFP). Emission detection windows were set as follows: mCFP, ~466–495 nm, EGFP, ~498–510 nm, EYFP, ~521–560 nm, RFP, ~590–650 nm. Images were processed and analyzed using Zeiss Zen 2.3 software (Microscopy GmbH, Oberkochen, Germany).

### 2.6. Clonogenic Cell Analysis

Clones in the outer and inner medulla from three mice per genotype were quantified independently by two blinded observers. Counting was terminated when a predefined threshold of observable clones was reached for each group. For control mice, the counting threshold was set at 891 clones. In the developmental models of *CAG^CreER^*; *Pkd1^fl/fl^*; *Rosa^Brainbow/^*^+^ and *CAG^CreER^*; *Pkd2^fl/fl^*; *Rosa^Brainbow/^*^+^, the threshold was 513 clones. For adult models, where cysts were significantly larger, counting was stopped after 291 clones. Regarding *CK19^CreER^*; *Pkd1^fl/^*^+^; *Rosa^Brainbow/^*^+^ and *CK19^CreER^*; *Pkd1^fl/fl^*; *Rosa^Brainbow/^*^+^ mice, the lower labeling efficiency of *CK19^CreER^* resulted in fewer observable clones compared to the *CAG^CreER^* models. As cyst development progressed in *CK19^CreER^*; *Pkd1^fl/fl^*; *Rosa^Brainbow/^*^+^ mice, each field of view often contained only a single, continuous monochromatic clone. Therefore, for this genotype at 4 and 6 months, counting was concluded once 228 clones had been recorded.

### 2.7. Plotting Cyst Growth Curve via Simplified Mathematical Model (Section 3.7)

The total surface area of a cyst (*A*_(*cyst*)(*t*)_) is the number of cyst lining cells at time *t* cross to surface area of cyst lining of each cell *A*_(*t*)_ at time *t*:

$$A_{\left(cyst)(t\right)}\;=\;N_{\left(t\right)}\;\times \;A_{\left(t\right)}\;=\;N_{\left(0\right)}\;\times \;[P_{1}\;\times \;2^{\frac{t}{td_{1}}}+P_{2}\;\times \;2^{\frac{t}{td_{2}}}+P_{3}]\;\times \;\frac{3\sqrt{3}}{8}{\left(d_{\left(o\right)}+\frac{\Delta d}{\Delta t}t\right)}^{2}$$

*P*_1_, *P*_2_, and *P*_3_ are the ratio of subpopulation of cells with different cystogenic potential, *P*_3_ cell is quiescent and do not proliferate. *P*_1_ + *P*_2_ + *P*_3_ = 1.
*N*_(*t*)_ is total number of cells at time *t*;
*N*_(0)_ is the initial number of cystogenic cells;
*dt*_1_ is the doubling time for *P*_1_ population, *dt*_2_ is the doubling time for *P*_2_ population.

Given the surface area of a sphere is *A* = π*D*^2^, the cyst diameter over time can be calculated as:

$$D_{cyst(t)}\;=\;\sqrt{\frac{A_{\left(cyst\right)\left(t\right)}}{\pi }}\;=\;\sqrt{\frac{N_{\left(t\right)}\cdot A_{\left(t\right)}}{\pi }}$$

To further refine our model of cyst expansion, we incorporated clonal heterogeneity by assigning distinct proliferative capacities to three clonal subpopulations as inferred from lineage tracing in adult onset models. *P*_1_ (highly cystogenic potential): 5% of initial cell pool with a doubling time of 10 days. *P*_2_ (medium cystogenic): 45% of cells, with a doubling time of 20 days, and *P*_3_ (non-cystogenic): 50% of cells.

To illustrate the mathematical model, we calculate the estimated total cyst area at 30 days post tamoxifen induction, assuming an initial mutant cell pool of 10 cystogenic cells.

$$N_{\left(0\right)}\;=\;10\;\mathrm{cells}\;A_{\left(cyst\right)\left(30\right)}\;=\;N_{\left(30\right)}\;\times \;A_{\left(30\right)}\;=\;10\;\times \;\left(0.05\;\times \;2^{30/10}+0.45\;\times \;2^{30/20}+0.5\right)\;\times \;\frac{3\sqrt{3}}{8}\;\times \;(12.53+0.118\times 30{)}^{2}\;\approx \;22\;\mathrm{cells}\;\times \;167.8\;{\mu m}^{2}\;\approx \;3691.6\;{\mu m}^{2}.\;D_{cyst(30)}\;\approx \;34.3\;\mu m.$$

At 30 days post induction, based on the model, the cyst is estimated to consist of ~22 epithelial cells, each with an apical area of ~168 μm^2^, giving a total cyst surface area of approximately 3691.6 μm^2^, and cyst diameter is ~34.3 μm.

At 31 days post induction, the cyst contains ~22 cells. For the next phase of cyst growth (31 to 90 days after induction, we assume: 20% of these cells become highly cystogenic (doubling time = 10 days), 30% remain medium cystogenic (doubling time = 20 days), and 50% cells remain quiescent based on adult onset models.

$$A_{\left(cyst)(90\right)}\;=\;N_{\left(90\right)}\;\times \;A_{\left(90\right)}\;=\;22\;\times \;\left(0.20\;\times \;2^{60/10}+0.30\;\times \;2^{60/20}+0.5\right)\;\times \;\frac{3\sqrt{3}}{8}\;\times \;{\left(12.53+0.118\;\times \;90\right)}^{2}\;\approx \;345.4\;\mathrm{cells}\;\times \;348.1\;{\mu m}^{2}\;\approx \;{120,227.4\;\mu m}^{2}.\;D_{cyst(90)}\;\approx \;195.6\;\mu m.$$

At 90 days post induction, based on the model, the cyst is estimated to consist of ~345.5 epithelial cells, each with an apical area of ~348.1 μm^2^, giving a total cyst surface area of approximately ~120,227.4 μm^2^, and cyst diameter is ~195.6 μm.

At day 91, the population is ~345.4 cells. For the next phase of cyst growth (91 to 150 days after induction), we assume the following: 30% cells become highly cystogenic (doubling time = 10 days), 30% cells remain medium cystogenic (doubling time = 20 days), and 40% cells remain quiescent.

$$A_{\left(cyst\right)\left(150\right)}\;=\;N_{\left(150\right)}\;\times \;A_{\left(150\right)}\;=\;345.4\;\times \;\left(0.3\;\times \;2^{60/10}+0.3\;\times \;2^{60/20}+0.4\right)\;\times \;\frac{3\sqrt{3}}{8}\;\times \;(12.53+0.118\;\times \;150{)}^{2}\;\approx \;7598.8\;\mathrm{cells}\;\times \;593.5\;{\mu m}^{2}\;\approx \;4,474,635.6\;{\mu m}^{2}.$$

$$D_{cyst(150)}\approx 1193.5\;\mu m.$$

At 150 days post induction, based on the model, the cyst is estimated to consist of ~7598.8 epithelial cells, each with an apical area of ~593.5 μm^2^, giving a total cyst surface area of approximately ~4,474,635.6 μm^2^, and cyst diameter is ~1193.5 μm.

### 2.8. Cysts Diameter Measurement

H&E-stained kidney sections were scanned using an Olympus microscope (Hachioji, Tokyo, Japan). Cyst diameter was defined as the distance between the two most distant points of each cyst on the section (which represents an underestimate of the true three-dimensional diameter) and was measured using ImageJ 1.53e software (Bethesda, MD, USA).

### 2.9. Western Blot

Mouse kidney tissues were homogenized in ice-cold RIPA lysis buffer (150 mM NaCl, 50 mM Tris-HCl pH 8.0, 1% Triton X-100, 0.5% sodium deoxycholate, 0.1% SDS, 1 mM EDTA) containing protease and phosphatase inhibitors (Beyotime, Songjiang, Shanghai, China). The homogenates were incubated on ice for 30 min and centrifuged at 16,000× *g* for 10 min. The supernatant was mixed with 5× SDS loading buffer and denatured at 95 °C for 10 min. Equal amounts of protein were separated by 10–12.5% SDS-PAGE (EpiZyme Biotech, Minhang, Shanghai, China) and electrophoretically transferred onto PVDF membranes (Millipore, Burlington, MA, USA). After blocking with 5% fat-free milk for 1 h at room temperature, membranes were washed three times with TBST (5 min each) and incubated overnight at 4 °C with the specified primary antibodies. Following another three TBST washes, membranes were incubated for 1 h at room temperature with the appropriate horseradish peroxidase (HRP)-conjugated secondary antibodies, then washed again. The primary antibodies used were: rabbit anti-Ift88 (1:2000, 13967-1-AP, Proteintech, Donghu, Wuhan, China), mouse anti-Polycystin-2 (1:2000, Santa Cruz, sc-228331, Dallas, TX, USA), and rabbit anti-Hsp90 (1:2000, 13171-1-AP, Proteintech, Donghu, Wuhan, China). Corresponding secondary antibodies were: HRP-conjugated goat anti-rabbit IgG (1:5000, Proteintech) and HRP-conjugated goat anti-mouse IgG (1:5000, Proteintech, Donghu, Wuhan, China). Protein bands were visualized using a chemiluminescence substrate (Abclonal, Donghu, Wuhan, China) and imaged with a Vilber Fusion Solo S imaging system using Evolution-Capt V18.00 software (Marne-la-Vallée, France).

### 2.10. Immunofluorescence

Immunofluorescence staining was performed on 10 μm cryosections. Sections were incubated with the following primary antibodies: rabbit anti-Arl13b (1:200, 17711-1-AP, Proteintech, Donghu, Wuhan, China) and mouse anti-Aqp2 (1:50, sc-515770, Santa Cruz, Dallas, TX, USA). After washing, sections were incubated with appropriate secondary antibodies: goat anti-rabbit IgG (H + L)-Alexa Fluor 594 (cross-adsorbed, Invitrogen, A11008, Waltham, MA, USA) and goat anti-mouse IgG (H + L)-Alexa Fluor 488 (cross-adsorbed, Invitrogen, A32728, Waltham, MA, USA). Following secondary antibody incubation, nuclei were counterstained with DAPI dihydrochloride (YEASEN, Pudong, Shanghai, China) before mounting. Images were acquired using a Nikon ECLIPSE Ti2-U microscope equipped with a 10 × 1.5 numerical aperture objective and controlled by NIS-Elements 5.10 software (Nikon, Corporation, Tokyo, Japan).

### 2.11. Morphometric Analysis of Renal Tubular Epithelial Cells

Morphometric analysis was performed on photomicrographs using ImageJ software. The scale of each image was set based on the embedded scale bar to ensure accurate calibration of spatial measurements.

Cell height measurement: The height of renal tubular epithelial cells was defined as the perpendicular distance from the basement membrane to the apical lumen. Measurements were performed by manually tracing this distance using the freehand line tool. The “ROI Manager” tool under the “Analyze” menu was used to store and manage all regions of interest (ROIs), and the “Measure” function was applied to record the values.

Mean cell width estimation: The cellular width was estimated indirectly. The luminal circumference of transversely sectioned tubules was traced and measured. The number of nuclei bordering the lumen was then counted. The mean cellular width for each tubule was calculated by dividing the luminal circumference by the number of cells. This analysis was performed on 15–22 individual tubules per group, with the results representing the average mean width.

## 3. Results

### 3.1. Lineage Tracing of Individual Renal Epithelial Cells for Cyst Formation in Developmental Models of ADPKD

**Figure 1:**
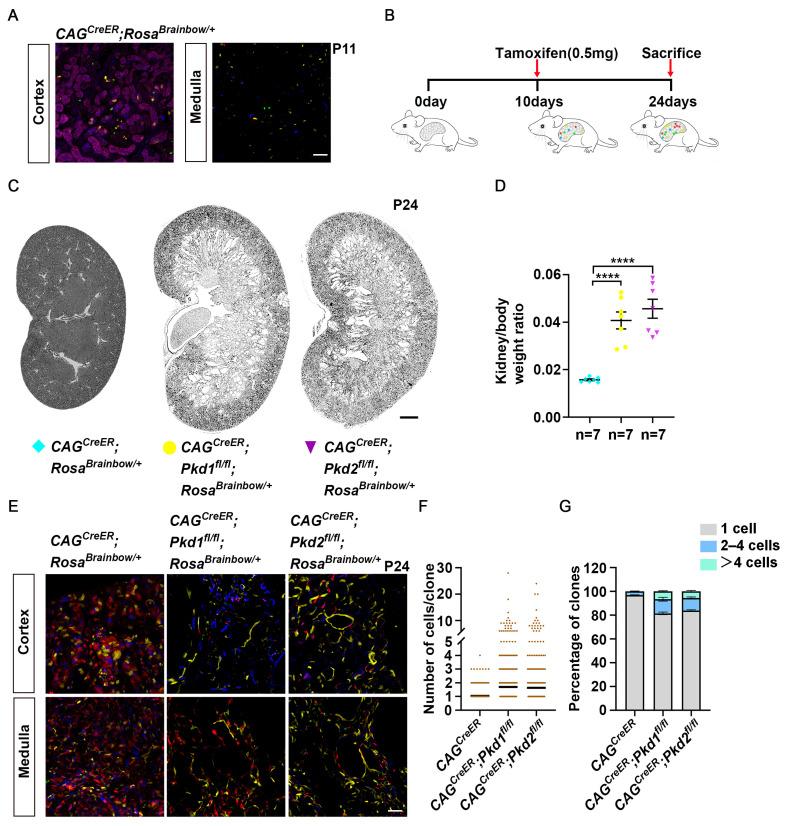
Lineage tracing of individual *Pkd1* or *Pkd2* mutant epithelial cells during cyst formation in developmental ADPKD models. (**A**) Confocal images of the cortical and medullary region from kidney sections of *CAG^CreER^*; *Rosa^Brainbow/^*^+^ mice at *P*_11_ following induction with tamoxifen at *P*_10_. (**B**) Schematic illustration of lineage tracing strategy in the early-onset ADPKD model. Tamoxifen-induced recombination simultaneously inactivates *Pkd1* in individual cells and labels them with distinct fluorophores at *P*_10_; cystic phenotypes were analyzed at *P*_24_. (**C**) Representative images of hematoxylin and eosin-stained kidney sections of *CAG^CreER^*; *Rosa^Brainbow/^*^+^, *CAG^CreER^*; *Pkd1^fl/fl^*; *Rosa^Brainbow/^*^+^, and *CAG^CreER^*; *Pkd2^fl/fl^*; *Rosa^Brainbow/^*^+^ mice at *P*_24_ after a single tamoxifen dose at *P*_10_. (**D**) Aggregated data of the kidney weight to body weight ratio of the mice. Data represent mean ± SEM; n = 7 mice per group; **** *p* < 0.0001 by unpaired two-tailed *t*-test. (**E**) Representative confocal images of renal cortex and medulla of *CAG^CreER^*; *Rosa^Brainbow/^*^+^, *CAG^CreER^*; *Pkd1^fl/fl^*; *Rosa^Brainbow/^*^+^, and *CAG^CreER^*; *Pkd2^fl/fl^*; *Rosa^Brainbow/^*^+^ mice at *P*_24_. (**F**) Quantitative analysis of cell numbers in each clone. n = 3 mice per group. (**G**) Percentage distribution (means ± SEM) bar plot of clone size. A total of 891 clones were analyzed in control (*CAG^CreER^*; *Rosa^Brainbow/^*^+^) kidneys, and 513 clones each in *Pkd1* and *Pkd2* knockouts. Scale bars: (**A**,**E**): 50 μm. (**C**): 1 mm.

To trace individual renal epithelial cells during cyst formation, we first generated *CAG^CreER^*; *Rosa^Brainbow/^*^+^ mice and administered a single dose of tamoxifen at postnatal day 10 (*P*_10_). Examination at *P*_11_ revealed that Cre mediated recombination stochastically induced expression of distinct fluorophores, including nuclear GFP, membrane CFP, cytoplasmic RFP or YFP (Figure S1). Sparse labeling was achieved under this induction condition: 98.65 ± 0.19% of cells expressed a single fluorophore without labeled neighbors, while only 0.67 ± 0.26% of cells had neighboring cells expressing the same fluorophore (Figure 1A, Table S1). This ensured effective resolution for lineage tracing at the single cell level during cyst formation.

Next, we generated and validated *Pkd1-flox* and *Pkd2-flox* mice (Figures S2A–G and S3A–H) and applied the lineage tracing system to inducible the inducible polycystic kidney disease model. We crossed *Rosa^Brainbow^* with *CAG^CreER^*; *Pkd1^fl/fl^* and *CAG^CreER^*; *Pkd2^fl/fl^* mice to obtain *CAG^CreER^*; *Pkd1^fl/fl^*; *Rosa^Brainbow/^*^+^ and *CAG^CreER^*; *Pkd2^fl/fl^*; *Rosa^Brainbow/^*^+^ mice. A single tamoxifen dose at *P*_10_ induced recombination, and kidneys were analyzed at *P*_24_ (Figure 1B). Both *Pkd1* and *Pkd2* mutant mice developed a cystic kidney phenotype by this time point (Figure 1C,D). Confocal imaging of 50 μm sections revealed contiguous stretches of cells expressing identical fluorophores within dilated tubules and cysts (Figure 1E). Quantification of clonal expansion showed minimal cell proliferation in control (*CAG^CreER^*; *Rosa^Brainbow/^*^+^) kidneys (1.04 ± 0.01cells/clone), with the largest clone containing 4 cells (Figure 1F–G, Table S1). Small clusters (2–4 cells) likely reflected limited cell division or coincidental neighboring cells expressing the same fluorophore. In contrast, kidneys from *CAG^CreER^*; *Pkd1^fl/fl^*; *Rosa^Brainbow/^*^+^ exhibited a significantly increased average clone size of 1.7 ± 0.1 cells, with 6.63 ± 0.52% of clones exceeding 4 cells. Similarly, *CAG^CreER^*; *Pkd2^fl/fl^*; *Rosa^Brainbow/^*^+^ kidneys displayed an average clone size of 1.64 ± 0.1 cells, with 5.65 ± 0.78% containing more than four cells. Notably, clones exceeding ten cells emerged in both mutant models. These findings indicate that a subset of *Pkd1* or *Pkd2* deficient epithelial cells undergo clonal expansion and contribute to cyst formation.

### 3.2. Lineage Tracing Individual Renal Epithelial Cells During Cyst Formation in Adult-Onset Models of ADPKD

**Figure 2:**
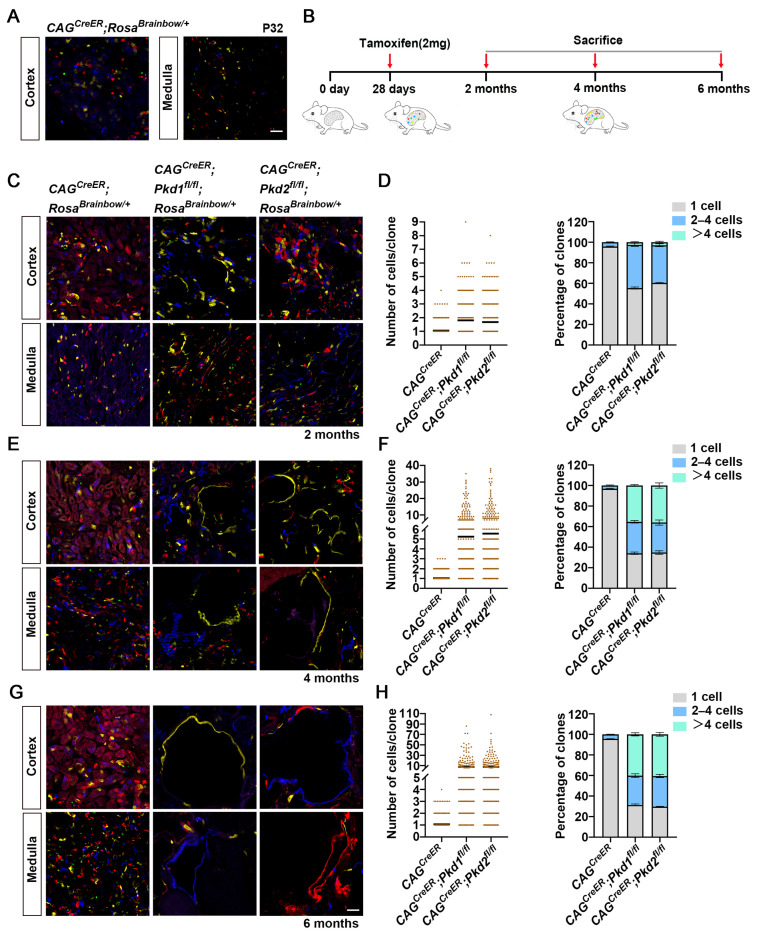
Lineage tracing of *Pkd1* or *Pkd2* mutant renal epithelial cells during cyst formation in adult-onset ADPKD models. (**A**) Confocal images of the cortical and medullary regions of kidney sections of *CAG^CreER^*; *Rosa^Brainbow/^*^+^ mice at *P*_32_ induced with tamoxifen at *P*_28_. (**B**) Schematic illustration of lineage tracing strategy used to follow *Pkd1* or *Pkd2* mutant cells during cystogenesis in an adult-onset renal cyst model. Tamoxifen was administered at *P*_28_, and phenotypes were analyzed at 2, 4, and 6 months. (**C**) Representative confocal images of cortical and medullary regions from the indicated genotypes at 2 months. (**D**) Quantitative analysis of cell numbers in each clone. n = 3 mice per group. The percentage (means ± SEM) distribution of clone sizes in *CAG^CreER^*; *Rosa^Brainbow/^*^+^, *CAG^CreER^*; *Pkd1^fl/fl^*; *Rosa^Brainbow/^*^+^, and *CAG^CreER^*; *Pkd2^fl/fl^*; *Rosa^Brainbow/^*^+^ mice. A total of 891 clones were analyzed in control (*CAG^CreER^*; *Rosa^Brainbow/^*^+^) kidneys and 513 clones in each *Pkd1* and *Pkd2* mutant group. (**E**) Representative confocal images of the cortex and medulla regions of kidney sections in mice at 4 months. (**F**) Quantitative analysis of cell numbers in each clone. n = 3 mice per group. The percentage (means ± SEM) distribution of clone sizes in *CAG^CreER^*; *Rosa^Brainbow/^*^+^, *CAG^CreER^*; *Pkd1^fl/fl^*; *Rosa^Brainbow/^*^+^, and *CAG^CreER^*; *Pkd2^fl/fl^*; *Rosa^Brainbow/^*^+^ mice. A total of 891 clones were analyzed in control (*CAG^CreER^*; *Rosa^Brainbow/^*^+^) and 291 in each *Pkd1* and *Pkd2* mutant group. (**G**) Representative confocal images of the cortical and medullary regions of kidney sections of mice at 6 months. (**H**) Quantitative analysis of cell numbers in each clone. n = 3 mice per group. The percentage (means ± SEM) distribution of clone sizes in *CAG^CreER^*; *Rosa^Brainbow/^*^+^, *CAG^CreER^*; *Pkd1^fl/fl^*; *Rosa^Brainbow/^*^+^, and *CAG^CreER^*; *Pkd2^fl/fl^*; *Rosa^Brainbow/^*^+^ mice. A total of 891 clones were analyzed in control (*CAG^CreER^*; *Rosa^Brainbow/^*^+^) and 291 in *Pkd1* and *Pkd2* mutant groups. Scale bar: (**A**,**C**,**E**,**G**): 50 μm.

To investigate the behavior of renal epithelial cells during cyst formation in adult-onset ADPKD, we used *CAG^CreER^*; *Rosa^Brainbow/^*^+^ mice, and administered a single dose of tamoxifen at *P*_28_. Examination at *P*_32_ validated that Cre-mediated recombination stochastically induced expression of distinct fluorescent proteins, resulting in sparsely labeled renal epithelial cells with 97.98 ± 0.19% cells having a mono-fluorophore and 1.01 ± 0.31% of cells having neighboring cells of the same fluorophore (Figure 2A, Table S1). We next induced recombination in *CAG^CreER^*; *Rosa^Brainbow/^*^+^, *CAG^CreER^*; *Pkd1^fl/fl^*; *Rosa^Brainbow/^*^+^, and *CAG^CreER^*; *Pkd2^fl/fl^*; *Rosa^Brainbow/^*^+^ mice with a single tamoxifen injection at *P*_28_ and analyzed the kidneys at 2, 4, and 6 months of age (Figure 2B). In both *Pkd1* and *Pkd2* mutant models, mild tubular dilation appeared approximately 2 months (~1 month after induction), progressing to moderate cystic kidney by 4 months (~3 months after induction), and severe cystic pathology by 6 months (~5 months after induction) (Figure S4A,B). In control *CAG^CreER^*; *Rosa^Brainbow/^*^+^ mice, clonal size distribution remained stable over time, with average clone size of 1.05 ± 0.01 cells at 2 months (95.85 ± 0.4% singlets, 4.15 ± 0.4% 2–4 cells), 1.04 ± 0.01 cells at 4 months (96.75 ± 0.49% singlets, 3.25 ± 0.49% 2–4 cells), and 1.06 ± 0.01 cells at 6 months (95.62 ± 0.19% singlets, 4.38 ± 0.19% 2–4 cells), with no clones exceeding four cells (Figure 2C–H, Table S1, Video S1). By contrast, *CAG^CreER^*; *Pkd1^fl/fl^*; *Rosa^Brainbow/^*^+^ kidneys exhibited progressive clonal expansion over time. The average clone size increased from 1.8 ± 0.05 cells at 2 months to 5.24 ± 0.36 cells at 4 months and 7.44 ± 0.71 cells at 6 months. The proportion of singlets decreased from 55.36 ± 1.09% to 34.02 ± 1.19% and further to 31.27 ± 0.91%, while clones exceeding 4 cells rose from 2.53 ± 0.52% (maximal clone size: 9 cells) to 35.4 ± 0.91% (maximal: 35 cells) and ultimately 40.21 ± 1.57% (maximal: 86 cells) during the same period.

Similarly, in *CAG^CreER^*; *Pkd2^fl/fl^*; *Rosa^Brainbow/^*^+^ kidneys, the average clone size increased from 1.68 ± 0.05 cells (2 months) to 5.55 ± 0.41 cells (4 months) to 7.77 ± 0.72 cells (6 months). The proportion of singlets declined from 60.43 ± 0.52% to 34.71 ± 1.72%, to 29.55 ± 0.34%, while clones > 4 cells increased from 2.92 ± 0.89% at 2 months to 36.08 ± 2.59% at 4 months to 40.55 ± 1.82% (maximum 108 cells) at 6 months (Figure S5A,B, Video S2). Together, these data demonstrated that a subset of renal epithelial cells with *Pkd1* or *Pkd2* inactivation undergo clonal expansion. The rate of expansion is modest during the early dilated tubule phase but accelerates markedly once cysts are established, suggesting a transition from slow proliferation to rapid clonal expansion as cystogenesis progresses.

### 3.3. Renal Epithelial Cells Expansion Is Reduced in Pkd1; Ift88 Double Mutant in an Adult-Onset ADPKD Model

**Figure 3:**
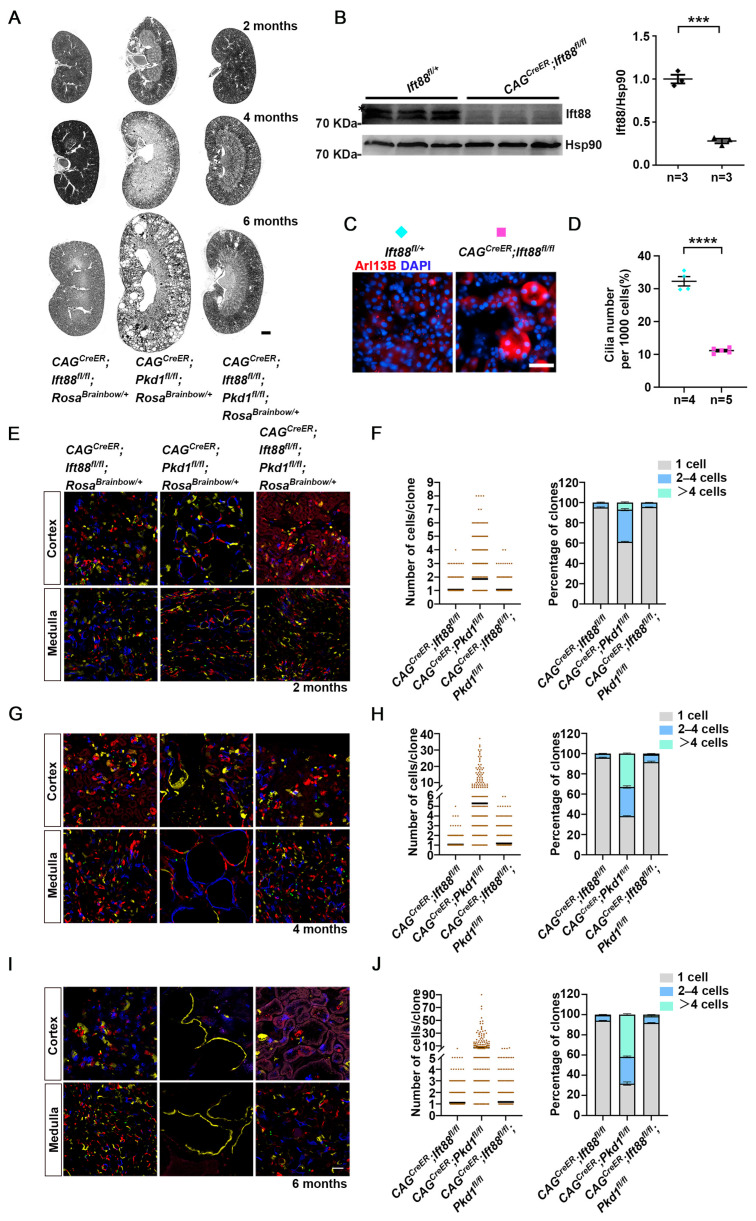
Cilia ablation reduces epithelial cell expansion in adult-onset *Pkd1* mutant kidneys. (**A**) Representative hematoxylin and eosin-stained kidney sections from *CAG^CreER^*; *Ift88^fl/fl^*; *Rosa^Brainbow/^*^+^, *CAG^CreER^*; *Pkd1^fl/f^*; *Rosa^Brainbow/^*^+^ and *CAG^CreER^*; *Ift88^fl/fl^*; *Pkd1^fl/fl^*; *Rosa^Brainbow/^*^+^ mice at 2, 4, and 6 months. (**B**) Immunoblot of kidney lysates from control (*Ift88^fl/^*^+^) and tamoxifen-induced *CAG^CreER^*; *Ift88^fl/fl^* mice using anti-Ift88 and Hsp90 antibodies (loading control), and aggregated data of Ift88-to-Hsp90 densitometry ratio from the kidney lysates of control (*Ift88^fl/^*^+^) and *CAG^CreER^*; *Ift88fl^/fl^* mice induced with tamoxifen at *P*_28_. Each data point represents one mouse. Numbers of biological replicates are indicated below each plot. (**C**) Immunofluorescence of kidney sections from control (*Ift88^fl/^*^+^) and tamoxifen-induced *CAG^CreER^*; *Ift88^fl/fl^* mice at 6 months old, stained with anti-Arl13b antibody. (**D**) Quantification of cilia number per 1000 kidney cells in kidney sections from control (*Ift88^fl/^*^+^) mice and tamoxifen-induced *CAG^CreER^*; *Ift88^fl/fl^* mice. Each data point represents one mouse. Numbers of biological replicates are indicated below each plot. (**E**,**G**,**I**) Representative confocal images of the renal cortex and medulla from the indicated genotypes at 2, 4, and 6 months, respectively. (**F**,**H**,**J**) Quantitative analysis of cell numbers in each clone (n = 3 mice per group) and the corresponding bar graphs showing the percentage distribution (means ± SEM) of clone sizes at 2, 4, and 6 months, respectively. A total of 513 clones (at 2 months) and 291 clones (at 4 and 6 months) were analyzed in *Pkd1* KO mice, while 891 clones were analyzed in both *Ift88* KO and *Pkd1/Ift88* KO mice. (**B**,**D**) Data are means ± SEM; Unpaired two-tailed *t* test; *** *p* < 0.001, **** *p* < 0.0001. Scale bars: (**A**): 1 mm. (**C**,**E**,**G**,**I**): 50 μm.

Since cyst growth in ADPKD is dependent on intact cilia, we examined whether loss of cilia affects the expansion capacity of *Pkd1* deficient renal epithelial cells. To this end, we generated *CAG^CreER^*; *Ift88^fl/f^*; *Rosa^Brainbow/^*^+^ and *CAG^CreER^*; *Ift88^fl/fl^*; *Pkd1^fl/fl^*; *Rosa^Brainbow/^*^+^ mice. Consistent with previous studies [12], ablation of cilia via inactivation of *Ift88* slowed cyst growth in *CAG^creER^*; *Ift88^fl/fl^*; *Pkd1^fl/fl^*; *Rosa^Brainbow/^*^+^ mice, which exhibited milder cystic pathology compared with *Pkd1* single mutants (Figure 3A).

Immunoblot analysis confirmed a substantial reduction in Ift88 protein level in kidneys from *CAG^CreER^*; *Ift88^fl/fl^* mice following tamoxifen induction (Figure 3B), and immunofluorescence staining for Arl13b verified efficient cilia ablation in renal tubules (Figure 3C,D). Quantification revealed that *CAG^CreER^*; *Ift88^fl/fl^*; *Rosa^Brainbow/^*^+^ mice displayed no significant cell expansion from 2 to 6 months, with mean clone sizes remaining stable at 1.06 ± 0.01 cells at 2 months, 1.06 ± 0.01 cells at 4 months, and 1.11 ± 0.02 cells at 6 months. The proportion of singlets also remained consistent (95.29 ± 0.51%, 95.96 ± 0.51% and 93.94 ± 0.39%, respectively) (Figure 3E–J, Table S1). Similarly, *CAG^CreER^*; *Ift88^fl/fl^*; *Pkd1^fl/fl^*; *Rosa^Brainbow/^*^+^ mice exhibited no aberrant cell expansion. Apart from a subtle decrease in single-cell clone percentage (from 95.62 ± 0.19% to 91.69 ± 0.92% to 91.81 ± 0.4%) and a marginal increase in 2–4 cell clones (from 4.38 ± 0.19% to 7.41 ± 0.85% to 6.62 ± 0.49%), no other significant changes were observed.

Together, these findings demonstrate that loss of cilia markedly diminishes the proliferative and expansion capacity of *Pkd1* mutant epithelial cells during cystogenesis, underscoring the essential role of cilia in driving clonal growth in ADPKD.

### 3.4. Lineage Tracing of Individual Biliary Epithelial Cells for Cystogenic Cells in the ADPKD Model

**Figure 4:**
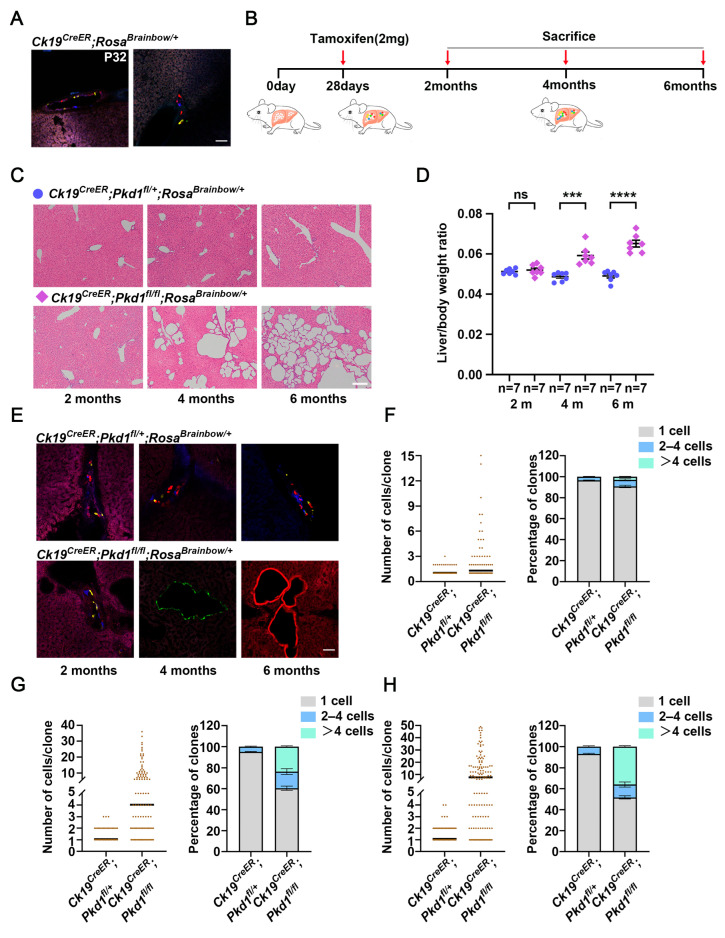
Lineage tracing of individual cholangiocytes for cyst formation in ADPKD model. (**A**) Representative confocal images of liver sections from *CK19^CreER^*; *Rosa^Brainbow/^*^+^ mice at *P*_32_ following tamoxifen induction at *P*_28_. (**B**) Schematic illustrating the lineage tracing strategy for bile duct cells in ADPKD. Mice were induced with tamoxifen at *P*_28_, and phenotypes were analyzed at 2, 4, and 6 months. (**C**) Representative H&E-stained liver sections from *CK19^CreER^*; *Pkd1^fl/^*^+^; *Rosa^Brainbow/^*^+^ and *CK19^CreER^*; *Pkd1^fl/fl^*; *Rosa^Brainbow/^*^+^ mice at 2, 4, and 6 months. Mice were induced with tamoxifen at *P*_28_. (**D**) Aggregated data of liver weight to body weight ratio of *CK19^CreER^*; *Pkd1^fl/^*^+^; *Rosa^Brainbow/^*^+^ and *CK19^CreER^*; *Pkd1^fl/fl^*; *Rosa^Brainbow/^*^+^ mice at 2, 4, and 6 months. Mice were induced with tamoxifen at *P*_28_. Data are represented as mean ± SEM; n = 7 mice per group; ns (no significant), *** *p* < 0.001, **** *p* < 0.0001 by unpaired two-tailed *t*-test. (**E**) Representative confocal images of liver sections from *CK19^CreER^*; *Pkd1^fl/^*^+^; *Rosa^Brainbow/^*^+^; and *CK19^CreER^*; *Pkd1^fl/fl^*; *Rosa^Brainbow/^*^+^ mice at 2, 4 and 6 months. (**F**) Quantification of the cell numbers per clone and the percentage (means ± SEM) distribution of clone sizes in cholangiocytes at 2 (**F**), 4 (**G**), and 6 (**H**) months. n = 3 mice per group. A total of 375 clones were analyzed in control (*CK19^CreER^*; *Pkd1^fl/^*^+^; *Rosa^Brainbow/^*^+^) mice versus 363 clones in *CK19^CreER^*; *Pkd1^fl/fl^*; *Rosa^Brainbow/^*^+^ mice at 2 months, 456 vs. 228 at 4 months, and 387 versus 228 at 6 months. Scale bar: (**A**,**E**): 50 μm. (**C**): 200 μm.

To trace the behavior of biliary epithelial cells and their contribution to hepatic cyst formation, we used *CK19^CreER^* mice [33] to selectively inactivate *Pkd1* in cholangiocytes and simultaneously label individual cells with distinct fluorophores. *CK19^CreER^*; *Rosa^Brainbow/^*^+^ mice were administered a single dose of tamoxifen at *P*_28_, resulting in stochastic expression of fluorophores. Analysis showed that 97.25 ± 0.34% cells expressed mono-fluorophore, and 1.37 ± 0.63% of cells had neighboring cells expressing the same fluorophore (Figure 4A, Table S1).

We next generated *CK19^CreER^*; *Pkd1^fl/^*^+^; *Rosa^Brainbow/^*^+^ and *CK19^CreER^*; *Pkd1^fl/fl^*; *Rosa^Brainbow/^*^+^ mice (Figure 4B). Inactivation of *Pkd1* in cholangiocytes led to progressive cystic liver pathology, with cyst severity increasing from 2 to 6 months (Figure 4C,D). In control *CK19^CreER^*; *Pkd1^fl/^*^+^; *Rosa^Brainbow/^*^+^ mice, the average clone size and distribution of clone sizes remained stable over time (Figure 4E–H, Table S1, Video S3). In contrast, *CK19^CreER^*; *Pkd1^fl/fl^*; *Rosa^Brainbow/^*^+^ experimental mice exhibited marked and progressive clonal expansion of cholangiocytes. The average clone size increased from 1.31 ± 0.07 cells at 2 months to 4.03 ± 0.41 cells at 4 months, and 7.63 ± 0.74 cells at 6 months (Figure 4E–H and Figure S6, Table S1, Video S4). The proportion of single-cell clones declined from 90.63 ± 1.2% at 2 months to 60.53 ± 2.01% at 4 months and 51.75 ± 1.58% at 6 months. Conversely, clones containing >4 cells increased from 3.03 ± 0.28% at 2 months to 23.68 ± 0.76% at 4 months and 35.96 ± 0.51% at 6 months. The largest clone observed at 6 months contained 49 cells. Together, these findings indicate that hepatic cysts arise from clonally expanding *Pkd1* deficient cholangiocytes, mirroring the clonal epithelial expansion observed in renal cystogenesis.

### 3.5. Measure Cell Shape Changes in the Course of Cyst Formation

**Figure 5:**
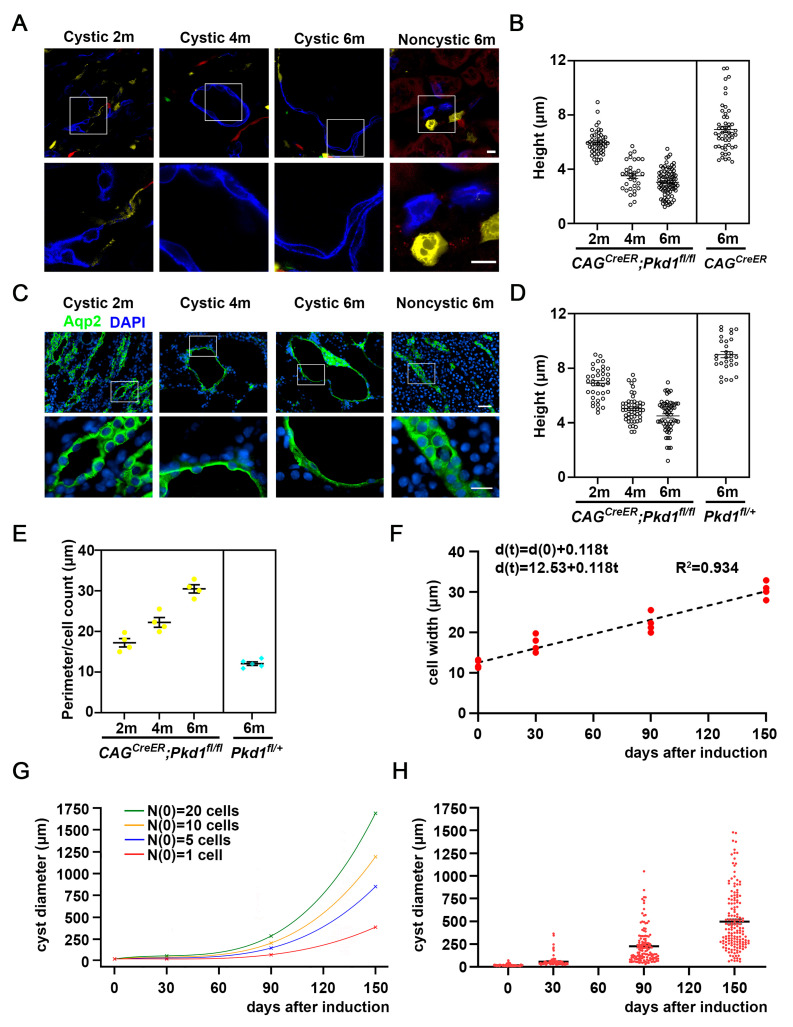
Cell shape remodeling during cyst formation and a mathematical model of cyst formation. (**A**) Representative confocal images of kidney sections from *CAG^CreER^*; *Rosa^Brainbow/^*^+^ mice at 6 months, and *CAG^CreER^*; *Pkd1^fl/fl^*; *Rosa^Brainbow/^*^+^ mice at 2 months, 4 months, and 6 months. Mice were induced with tamoxifen at *P*_28_. (**B**) Scatter plots showing the heights of individual renal epithelial cells in *CAG^CreER^*; *Pkd1^fl/fl^*; *Rosa^Brainbow/^*^+^ mice at 2 months, 4 months, and 6 months, as well as in *CAG^CreER^*; *Rosa^Brainbow/^*^+^ mice at 6 months. Each data point represents a single tubular or cyst-lining cell. (**C**) Immunofluorescence staining of kidney sections with anti-Aqp2 antibodies in *CAG^CreER^*; *Pkd1^fl/fl^* mice at 2, 4, and 6 months, and control (*Pkd1^fl/^*^+^) kidney at 6 months. *CAG^CreER^*; *Pkd1^fl/fl^* mice were induced with tamoxifen at *P*_28_. (**D**) The scatter plot quantifying epithelial cell height in *CAG^CreER^*; *Pkd1^fl/fl^* kidney at 2, 4, and 6 months, and control (*Pkd1^fl/^*^+^) kidney at 6 months. Each symbol corresponds to a single epithelial cell measurement. (**E**) Quantitative analysis of luminal organization showing the ratio of Aqp2-labeled collecting ducts perimeter to the number of Aqp2-positive cells. Data were collected from control (*Pkd1^fl/^*^+^) kidneys (15 lumens, n = 295 cells), and *Pkd1* mutant kidneys at 2 months (20 cystic lumens, n = 468 cells), 4 months (22 cystic lumens, n = 472 cells), and 6 months (20 cystic lumens, n = 501 cells). (**F**) Plot cystic cell width at 30 days, 90 days, and 150 days after induction. d_(t)_ = 12.53 + 0.118t, R^2^ = 0.934. (**G**) Plot the diameter of individual cysts at 30, 90, and 150 days after induction from *N*_(0)_ = 1, *N*_(0)_ = 5, *N*_(0)_ = 10, and *N*_(0)_ = 20 cells. (**H**) Measurement of cyst diameters from randomly picked regions of H&E-stained sections of *CAG^CreER^*; *Pkd1^fl/fl^* mice at ~30 days (2 months), ~90 days (4 months), and ~150 days (6 months), and control (*Pkd1^fl/^*^+^) mice at 2 months. Each data point is the individual cyst diameter; data are means ± SEM. Scale bars: (**A**): upper panels, 10 μm; lower panels, 10 μm. (**C**): upper panels, 50 μm; lower panels, 20 μm.

Cell shape change is another key cellular event associated with cyst progression [27,36]. To evaluate this, we first measured the height of cyst-lining epithelial cells from apical to the basal surface. Notably, we observed a reduction in cell height as early as 1 month after tamoxifen induction, with a progressive decline evident at 3 and 5 months after tamoxifen induction (Figure 5A,B). To assess changes in cell width, we performed immunofluorescence staining with anti-Aqp2 antibody, followed by quantification of peri-luminal cell width, calculated as luminal perimeter divided by the number of lining cells. These measurements confirmed trends observed with in vivo fluorescence lineage tracing, a gradual decrease in cell height accompanied by a concurrent increase in apical cell width over time (Figure 5C–E).

Interestingly, the apical width of epithelial cells increased linearly over time, fitting a simple linear regression model (Figure 5F). The relationship between cell width and time (*t*) is described by the following equation

$$d_{\left(t\right)}\;=\;d_{\left(0\right)}+\frac{\Delta d}{\Delta t}t$$

where

*d*_(*t*)_ is the long diagonal at time *t*.
*d*_(0)_ is the initial diagonal.
$\frac{\Delta d}{\Delta t}$ represents the rate of lateral expansion per unit time (slope).

$$d_{\left(t\right)}\;=\;12.53+0.118t$$

Assuming the epithelial cells hexogen packing, the surface area of cell is: *A* = $\frac{3\sqrt{3}}{8}d^{2}$.

So, each cystic epithelial cell surface area over time is

$$A_{\left(t\right)}\;=\;\frac{3\sqrt{3}}{8}d_{\left(t\right)}^{2}\;=\;\frac{3\sqrt{3}}{8}(d_{\left(0\right)}+\frac{\Delta d}{\Delta t}t{)}^{2}$$

These results support the idea that cyst progression is driven in part by progressive epithelial flattening and lateral expansion.

### 3.6. Mathematical Model of Cyst Growth by Coupling Clonal Expansion and Cell Shape Change

The formation of cysts in ADPKD is a dynamic tissue remodeling process, characterized by a continuous increase in cyst diameter while preserving monolayer epithelial architecture. This consistent monolayered organization offers a predictable geometric framework, enabling the development of a mathematical model that links cell proliferation (clonal expansion) and cell shape transformation to the overall growth of the cysts.

Assuming a constant proliferation rate, no cell cycle exit, and a fixed doubling time (*dt*), the number of cyst-lining epithelial cells at time *t* is given by:

$$N_{\left(t\right)}\;=\;N_{\left(0\right)}\;\times \;2^{\frac{t}{dt}}$$

where:

*N*_(*t*)_ is total number of cells at time *t*.
*N*_(0)_ is the initial number of cystogenic cells, and *dt* is the doubling time.

Cystogenic cells are subject to dynamic variability throughout the course of cystogenesis, influenced by heterogeneous cell cycle dynamics, microenvironmental influences, and local signaling gradients. These sources of variability can result in disproportionate contributions of individual cells to cyst formation. Additionally, some sibling cells may transiently exit cell cycle, further increasing variability in their proliferative output. Therefore, when modeling cyst growth, the total number of cyst-lining epithelial cells derived from a population of initial cystogenic cells should be considered as the sum of independent trajectories.

Assuming the initial number of cystogenic cells is *k*, each cell may follow a distinct proliferation path due to stochastic and environmental factors. Thus, the total cyst cell population *N*_(*t*)_ over time can be expressed as the sum of expanding subclones:

$$N_{\left(t\right)}\;=\;2^{\int_{0}^{t}\frac{1}{{td}_{1}(T)}dT}+2^{\int_{0}^{t}\frac{1}{td_{2}(T)}dT}+\ldots \ldots +2^{\int_{0}^{t}\frac{1}{td_{k}(T)}dT}$$

where:

*N*_(*t*)_ is total number of cells at time *t*.
*td*_1_(*T*), *td*_2_(*T*) …. *td_k_*(*T*) define the doubling time of starting cystogenic cell 1, 2, *… k* at time *t*.
$\int_{0}^{t}\frac{1}{td_{k}\left(T\right)}dT$ define the doubling time of cystogenic *k* cell from time 0 to *t*.

The total surface area of a cyst would be:

$$A_{\left(cyst)(t\right)}\;=\;N_{\left(t\right)}\;\times \;A_{\left(t\right)}\;=\;[2^{\int_{0}^{t}\frac{1}{td_{1}\left(T\right)}dT}+2^{\int_{0}^{t}\frac{1}{td_{2}\left(T\right)}dT}+\ldots \ldots +2^{\int_{0}^{t}\frac{1}{td_{k}\left(T\right)}dT}]\;\times \;\frac{3\sqrt{3}}{8}(d_{\left(o\right)}+\frac{\Delta d}{\Delta t}t{)}^{2}$$

Given the surface area of a sphere is *A* = π*D*^2^, the cyst diameter over time can be calculated as:

$$D_{cyst(t)}\;=\;\sqrt{\frac{A_{\left(cyst\right)\left(t\right)}}{\pi }}\;\;=\;\;\sqrt{\frac{N_{\left(t\right)}\cdot A_{\left(t\right)}}{\pi }}$$

### 3.7. Simplified Model of Heterogeneous Clonal Contribution Across Cyst Growth Phases

In the current experimental setting, it is not feasible to track the expansion capacity of each cell over the whole course of cyst formation. To enable tractable modeling while still capturing biological heterogeneity, we introduced a simplified population-based framework. We divide the total cystogenic population into three subpopulations.

$$N_{\left(t\right)}\;=\;N_{\left(0\right)}\;\times \;[P_{1}\;\times \;2^{\frac{t}{td_{1}}}+P_{2}\;\times \;2^{\frac{t}{td_{2}}}+P_{3}]$$

*P*_1_, *P*_2_, and *P*_3_ are the ratio of subpopulation of cells with different cystogenic potential, *P*_3_ cell is quiescent and do not proliferate. *P*_1_ + *P*_2_ + *P*_3_ = 1.
*N*_(*t*)_ is total number of cells at time *t*,
*N*_(0)_ is the initial number of cystogenic cells,
*dt*_1_ is the doubling time for *P*_1_ population, *dt*_2_ is the doubling time for *P*_2_ population.

The total surface area of a cyst would be:

$$A_{\left(cyst\right)\left(t\right)}\;=\;N_{\left(t\right)}\;\times \;A_{\left(t\right)}\;=\;N_{\left(0\right)}\;\times \;[P_{1}\;\times \;2^{\frac{t}{dt_{1}}}+P_{2}\;\times \;2^{\frac{t}{dt_{2}}}+P_{3}]\;\times \;\;\frac{3\sqrt{3}}{8}(d_{\left(0\right)}+\frac{\Delta d}{\Delta t}t{)}^{2}$$

Given the surface area of a sphere is *A* = π*D*^2^, the cyst diameter over time can be calculated as:

$$D_{cyst(t)}\;=\;\sqrt{\frac{A_{\left(cyst\right)\left(t\right)}}{\pi }}\;=\;\sqrt{\frac{N_{\left(t\right)}\cdot A_{\left(t\right)}}{\pi }}$$

We separate three phases of cyst growth from 0 to 30, from 31 to 90, and from 91–150 days after tamoxifen induction. We also assume a fixed doubling time for each phase doubling time for each subpopulation of cells. We plot the graph of cyst size (diameter), *N*_(0)_ = 1 cell, *N*_(0)_ = 5 cells, *N*_(0)_ = 10 cells, and *N*_(0)_ = 20 cells by time (Figure 5G). The detailed calculation could be found in the method section, clearly, the graph shows exponential growth. We measured the size of the individual cysts of *CAG^CreER^*; *Pkd1^fl/fl^* kidney at 1 month, 3 months, and 5 months after tamoxifen induction at *P*_28_, and plotted each data point; we found that there is a correlation of the actual size of the individual cysts, and the data points generated by the mathematical formula (Figure 5H).

## 4. Discussion

Here, using multicolor lineage tracing, we visualized the in vivo behavior of individual *Pkd1* or *Pkd2* mutant epithelial cells during cyst initiation and progression. Our findings reveal that the cystic epithelium is highly dynamic during cystogenesis. A subset of polycystin-deficient cells undergoes early clonal expansion during the initial tubule dilation phase, preceding the appearance of overt cysts. As cysts grow, additional subsets of previous non-expanding cells later enter the cell cycle and begin to proliferate. As cysts enlarge, their cyst lining becomes composed of clonally distinct patches, consisting of identically labeled cells, indicating sustained proliferation derived from single renal tubular epithelial cells or bile ductal cells. This clonal expansion behavior was observed in both kidneys and liver in adult-onset models, which closely mirror the growth dynamics observed in human ADPKD [37]. In parallel with clonal expansion, we observed progressive epithelial remodeling, beginning early and persisting throughout cyst enlargement. Specifically, epithelial cells underwent flattening and lateral spreading, resulting gradual increase in apical surface area. By integrating quantitative lineage data with measurement of cell morphological changes, we constructed a mathematical model that links clonal expansion and epithelial geometry to the resulting cyst lumen diameter. This framework provides a mechanistic explanation for how small populations of polycystin-deficient cells can drive sustained cyst growth over time.

We observed that epithelial cell height progressively decreases and transitions into flattened morphology already at the early tubule dilation stage, prior overt cyst formation. This flattening is a continuous and dynamic process, accompanied by lateral expansion of the cell body and a corresponding increase in apical surface area. Quantitative measurements revealed that the length of the apical diagonal of cyst-lining cells increases linearly over time, indicating a geometrically predictable transformation in cell shape. Importantly, this cell shape remodeling is cell-autonomous [36]. In a mosaic model generated by morula-stage aggregation of WT and *Pkd1^−/−^* cells, the resulting kidneys develop mosaic cysts composed of both WT cells and *Pkd1^−/^^−^* mutant cells. Within the same cysts, *Pkd1^−/^^−^* cells consistently adopt a flattened morphology, whereas adjacent WT cells maintain a columnar shape [36]. This observation suggests a connection between loss of polycystin, the disrupted ciliary signaling, and the initiation of cell flattening, implying the involvement of additional downstream mechanisms, possibly involving cytoskeletal remodeling and cell intrinsic shape regulation.

Our mathematical model couples two key biological drivers for cyst growth, epithelial cell proliferation and cell shape remodeling. By linking the dynamics of cell number increase with progressive lateral expansion of apical surface area, the model estimates total cyst surface area, which can then be used to calculate the cyst lumen diameter over time. This framework provides a scalable representation of cyst growth dynamics grounded in measurable cellular parameters.

Cellular heterogeneity is incorporated implicitly at the collective level rather than explicitly resolved at the single-cell resolution. Specifically, epithelial cell width and length are calculated as average values derived from total cyst perimeter divided by the number of epithelial cyst-lining cells, this approach captures global geometric behavior of the epithelium, while averaging out cell-to-cell variability. As such, the model does not explicitly distinguish between cuboidal and flattened epithelial subtypes, nor does it differentiate mutant from non-mutant contributors within the cyst lining.

Importantly, the model establishes an explicit quantitative link between cell-level properties—namely epithelial expansion and geometry—and the emergence of exponential cyst growth at the tissue level. In this regard, the model is primarily explanatory rather than predictive. Its purpose is not to forecast outcomes in its current form, but to formalize and integrate experimentally observed mechanisms into a coherent mathematical structure. As such, the model consolidates existing biological knowledge and provides a foundation that can support future hypothesis-driven refinements and experimental testing. With independent validation and incorporation of additional parameters, the framework could be extended toward predictive applications.

In ADPKD animal models, histological measurements closely correlate with MRI and ultrasound based imaging readouts [37,38], supporting the relevance of surface-based metrics for interpreting cyst morphology in vivo [39]. Imaging-derived metrics such as total cyst volume or total kidney volume reflect the integrated outcome of collective cyst expansion over time, driven by epithelial proliferate and cell shape remodeling. By providing a quantitative relationship between cell-scale behaviors and volumetric growth, our model may offer a conceptual framework for interpreting longitudinal imaging trajectories. In principle, this framework could help bridge cellular mechanisms with imaging-based disease monitoring and improve the biological interpretability of prognostic imaging tools. Integration with advanced radiological analysis approaches, including deep-learning based segmentation and volumetric tracking, may further enhance such application. However, a key distinction exists between humans and inducible mouse models. In human ADPKD, ongoing somatic “second hit” mutations continuously generate new cystogenic clones, each with variable genotype and proliferative potential, introducing stochasticity into overall cyst burden [3,4]. In contrast, Cre inducible mouse models have a fixed number of recombined cystogenic cells established at the time of induction, without subsequent mutational events. To improve the predictive accuracy of cyst growth algorithms, it may be necessary to incorporate parameters reflecting continual emergence of new clones, especially in the context of genetically unstable alleles such as *Pkd2^Ws25^* (ref. [4]), where spontaneous recombination or mutational events lead to progressive cyst initiation throughout the lifespan.

A limitation of our study is the use of ubiquitous inducible *CAG^CreER^* driver for lineage tracing, which induces fluorophore expression across the entire nephron. While this allows comprehensive labeling of cystic epithelial cells, it prevents segment-specific quantification of cyst expansion rates. Prior studies have demonstrated that collecting ducts develop more rapid cyst formation than the thick ascending limb [26,40]. Therefore, our finding may underrepresent segmental variation in cystogenesis. To address this, future studies should incorporate segment specific inducible Cre lines. For example, *Cdh16^CreER^* targets distal nephron [41], while *Ubc^CreER^* for the proximal tubule [42]. These approaches would allow for more refined analysis of the spatiotemporal dynamics of cyst initiation and expansion within each nephron segment. Additionally, our current model does not account for apoptosis, which, although relatively infrequent in cyst-lining epithelium, may contribute to cyst size regulation and turnover. Apoptotic events could influence both cell number and local remodeling; therefore, incorporating apoptosis into the model may refine the epithelial dynamics during cystogenesis.

In summary, by performing lineage tracing of individual epithelial cells with polycystin deficiency, we demonstrate that cyst expansion arises from coordinated interplay between clonal expansion and progressive cell shape remodeling. Our quantitative model captures the exponential “snowball” kinetics of cyst growth and might offer a conceptual framework for linking epithelial dynamics to imaging based prognostics in ADPKD.

## Data Availability

The data presented in this study are available in this paper.

## Supplementary Materials

The following supporting information can be downloaded at: https://www.mdpi.com/article/10.3390/cells15030297/s1, Figure S1: Four possible outcomes of the *Rosa^Brainbow/+^* locus after Cre-mediated recombination; Figure S2: Generating *Pkd1* conditional knockout allele in mice and inactivation of *Pkd1* yields polycystic kidney and liver diseases; Figure S3: Generating *Pkd2* conditional knockout allele in mice and inactivation of *Pkd2* yields polycystic kidney and liver diseases; Figure S4: Progressive cyst development following inactivation of *Pkd1* or *Pkd2* at P28; Figure S5: Representative supplementary views of confocal imaging of kidney sections from *CAG^CreER^; Pkd1^fl/fl^; Rosa^Brainbow/+^* and *CAG^CreER^; Pkd2^fl/fl^; Rosa^Brainbow/+^* mice; Figure S6: Supplementary confocal views of liver sections from *Ck19^CreER^; Pkd1^fl/fl^; Rosa^Brainbow/+^* mice (2–6 months old); Table S1: Summary of clone size distributions (single cells, 2–4 cells, and >4 cells) for all experimental and control groups in kidney and liver tissues; Table S2: List of all oligonucleotide primers employed for genotyping and molecular validation in mice models; Video S1: 3D reconstruction of 50 µm kidney sections from control *CAG^CreER^; Rosa^Brainbow/+^* mice at 4 and 6 months; Video S2: 3D reconstruction of 50 µm kidney sections from *CAG^CreER^; Pkd1^fl/fl^; Rosa^Brainbow/+^* and *CAG^CreER^; Pkd2^fl/fl^; Rosa^Brainbow/+^* mice at 4 and 6 months; Video S3: 3D reconstruction of 50 µm liver sections from control *CK19^CreER^; Pkd1^fl/+^; Rosa^Brainbow/+^* mice at 4 and 6 months; Video S4: 3D reconstruction of 50 µm liver sections from *CK19^CreER^; Pkd1^fl/fl^; Rosa^Brainbow/+^* mice at 4 and 6 months.

## Abbreviations

ADPKD	autosomal dominant polycystic kidney disease
PC1	polycystin-1
PC2	polycystin-2
CDCA	cilia dependent cyst activation
CT	Computed Tomography
MRI	magnetic resonance imaging
