# Supplementary material for: Epithelial Dynamics of Cystogenesis in Genetic Models of Autosomal Dominant Polycystic Kidney Disease
Source: Cells. 2026 Feb 4;15(3):297. doi: 10.3390/cells15030297 (PMC12896517; doi:10.3390/cells15030297)
Supplement: Supplementary file 1 [file cells-15-00297-s001.zip › Supplementary Figure Legends.pdf]

Supplementary Figure Legends

Figure S1 Four possible outcomes of the *Rosa*$^{Brainbow/+}$ locus after Cre-mediated recombination.
Schematic illustration showing the four distinct fluorescent protein expression outcomes at the *Rosa*$^{Brainbow/+}$ locus following Cre-mediated recombination. Each recombination event leads to stochastic expression of one of four fluorophores, enabling multicolor clonal labeling.

Figure S2 Generating *Pkd1* conditional knockout allele in mice and inactivation of *Pkd1* yields polycystic kidney and liver diseases.
(A) Schematic illustration of the *Pkd1* conditional allele, showing exon locations, homology arms, *loxP* sites, conditional knockout regions, and self-deletion anchor site flanking the neomycin selection cassette. Cre-mediated recombination results in excision of exons 12–14, generating a null *Pkd1* allele. Removal of the neomycin cassette occurs following germline transmission. Locations of primers F1, R1, F2, and R2 used for genotyping are indicated. (B) PCR genotyping of genomic DNA from WT, *Pkd1*$^{fl/+}$, and *Pkd1*$^{fl/fl}$ mice. Using primers F1/R1, WT mice yield a 296-bp band, *Pkd1*$^{fl/+}$ mice generate 296-bp and 385-bp bands, and *Pkd1*$^{fl/fl}$ mice generate a 385-bp band. Using primers F2/R2, WT mice yield a 707-bp band, *Pkd1*$^{fl/+}$ mice generate 707-bp and 787-bp bands, and *Pkd1*$^{fl/fl}$ mice generate a 787-bp band. (C) PCR analysis of genomic DNA from kidneys of wild-type and tamoxifen-induced *CAG*^{CreER}; *Pkd1*$^{fl/fl}$ mice using primers F1/R2. WT mice show no detectable PCR product, whereas *CAG*^{CreER}; *Pkd1*$^{fl/fl}$ mice generate a 474-bp band. (D) Representative scanned images of mouse kidney sections stained with hematoxylin and eosin of indicated genotypes at 18 weeks, and (E) aggregate quantitative data for kidney-body weight ratio of control (*Pkd1*$^{fl/+}$) and *CAG*^{CreER}; *Pkd1*$^{fl/fl}$ mice. (F) Representative scanned images of mouse livers section stained with hematoxylin and eosin of indicated genotypes at 18 weeks, and (G) aggregate quantitative data for liver-body weight ratio of control (*Pkd1*$^{fl/+}$) and *CAG*^{CreER}; *Pkd1*$^{fl/fl}$ mice. Mice were induced with tamoxifen from P28 to P32. Data are represented as mean ± SEM, and were analyzed using the unpaired, two-sided t test, ** $P < 0.01$, *** $P < 0.001$; The numbers of animals (n) are 6 in each group. Scale bars: D, 2 mm; 1mm, F, 2 mm; 0.5 mm.

Figure S3 Generating *Pkd2* conditional knockout allele in mice and inactivation of *Pkd2* yields polycystic kidney and liver diseases.
(A) Schematic illustration of the *Pkd2* conditional knockout allele, showing exon locations, homology arms, conditional knockout regions, and *loxP* sites. Cre-mediated recombination results in excision of exons 4–5, generating a null *Pkd2* allele. Locations of primers F1, R1, F2, and R2 used for genotyping are indicated. (B) PCR genotyping of genomic DNA from WT, *Pkd2*$^{fl/+}$, and *Pkd2*$^{fl/fl}$ mice. Using primers F1/R1, WT mice yield a 219-bp band, *Pkd2*$^{fl/+}$ mice yield 219-bp and 253-bp bands, and *Pkd2*$^{fl/fl}$ mice generate a 253-bp band. Using primers F2/R2, WT mice yield a 219-bp band, *Pkd2*$^{fl/+}$ mice generate 219-bp and 253-bp bands, and *Pkd2*$^{fl/fl}$ mice generate a 253-bp band. (C) PCR analysis of genomic DNA from kidneys of WT and *CAG*^{CreER}; *Pkd2*$^{fl/fl}$ mice induced with tamoxifen. Using primers F1/R2, WT mice generate no detectable PCR product, whereas *CAG*^{CreER}; *Pkd2*$^{fl/fl}$ kidneys generate a 275-bp band. (D) Immunoblot analysis using an anti-PC2 antibody to examine PC2 protein expression in kidney lysates from control (*Pkd2*$^{fl/+}$) and *CAG*^{CreER}; *Pkd2*$^{fl/fl}$ mice at 17 weeks of age. (E) Representative scanned images of mouse kidney sections stained with hematoxylin and eosin of indicated genotypes at 18 weeks, and (F) aggregate quantitative data for kidney-body weight ratio of control (*Pkd2*$^{fl/+}$) and *CAG*^{CreER}; *Pkd2*$^{fl/fl}$ mice. (G) Representative scanned images of mouse livers section stained with hematoxylin and eosin of indicated genotypes at 18 weeks, and (H) aggregate quantitative data for liver-body weight ratio of control (*Pkd2*$^{fl/+}$) and *CAG*^{CreER}; *Pkd2*$^{fl/fl}$ mice. Mice were induced with tamoxifen from P28 to P32. Data are represented as mean ± SEM, and were analyzed using the unpaired, two-sided t test, ** $P < 0.01$, *** $P < 0.0001$; The numbers of animals (n) are 6 in each group. Scale bars: E, 2 mm; 1mm, G, 2 mm; 0.5 mm.

Figure S4 Progressive cyst development following inactivation of *Pkd1* or *Pkd2* at P28.
(A) Representative hematoxylin and eosin-stained kidney sections from *CAG*^{CreER}; *Rosa*$^{Brainbow/+}$, *CAG*^{CreER}; *Pkd1*$^{fl/fl}$; *Rosa*$^{Brainbow/+}$, and *CAG*^{CreER}; *Pkd2*$^{fl/fl}$; *Rosa*$^{Brainbow/+}$ mice at 2, 4, and 6 months. Mice were induced with tamoxifen at P28.
(B) Aggregated data of kidney to body weight ratio of *CAG*^{CreER}; *Rosa*$^{Brainbow/+}$, *CAG*^{CreER}; *Pkd1*$^{fl/fl}$; *Rosa*$^{Brainbow/+,}$ and

CAG^{CreER}*; Pkd2*$^{fl/fl}$*; Rosa*$^{Brainbow/+}$ mice at 2, 4, and 6 months, the mice were induced with tamoxifen at P28. Data represent mean ± SEM; n=7 mice; Unpaired two-tailed t-test was used for comparison; ***P < 0.001, ****P < 0.0001. Scale bar: A: 1 mm.

Figure S5 Representative supplementary views of confocal imaging of kidney sections from *CAG*^{CreER}*; Pkd1*$^{fl/fl}$*; Rosa*$^{Brainbow/+}$ and *CAG*^{CreER}*; Pkd2*$^{fl/fl}$*; Rosa*$^{Brainbow/+}$ mice.
(A) The apparent discontinuity of observed clones stems from the two-dimensional optical sectioning of three-dimensional cysts; Z-stack scanning reveals continuous clonal architecture throughout the cystic structure. (B) Boxed region (red) highlights the largest observed clone (108 cells). Scale bar: A, B: 50 μm.

Figure S6 Supplementary confocal views of liver sections from *Ck19*^{CreER}*; Pkd1*$^{fl/fl}$*; Rosa*$^{Brainbow/+}$ mice (2–6 months old). Scale bar: 50 μm.
