# Supplementary material for: Epithelial Dynamics of Cystogenesis in Genetic Models of Autosomal Dominant Polycystic Kidney Disease
Source: Cells. 2026 Feb 4;15(3):297. doi: 10.3390/cells15030297 (PMC12896517; doi:10.3390/cells15030297)
Supplement: Supplementary file 1 [file cells-15-00297-s001.zip › Table S1.pdf]

Figure 1F,1G

24 days								
WT			PKD1 f/f CAG			PKD2 f/f CAG		
WT1	WT2	WT3	P1C1	P1C2	P1C3	P2C1	P2C2	P2C3
1	1	1	18	1	6	1	1	1
1	1	1	1	1	2	1	1	1
1	1	1	1	1	1	1	4	3
1	1	1	1	1	1	1	1	1
1	1	1	2	1	1	20	1	1
1	1	1	1	1	7	1	1	1
1	1	1	1	1	1	1	1	1
1	1	1	4	1	1	1	1	1
1	1	1	1	3	1	1	6	1
2	1	1	1	1	1	1	1	1
1	2	1	1	1	6	1	1	8
1	1	1	2	1	1	9	1	1
1	1	1	1	1	1	1	1	1
1	1	1	1	4	1	1	1	1
1	1	1	1	1	1	1	1	1
1	1	1	1	1	2	1	5	1
1	1	1	1	1	1	3	1	2
1	1	1	1	1	1	1	1	1
1	1	1	8	4	3	1	1	2
1	1	1	9	4	1	1	1	1
1	1	1	1	1	1	4	1	1
1	1	3	4	1	1	2	1	4
1	1	1	1	1	1	1	3	1
1	1	1	1	1	1	1	1	1
1	1	1	2	1	4	1	1	1
1	1	1	1	1	2	5	1	1
1	1	1	1	1	1	1	3	3
1	1	1	1	4	1	1	1	1
1	1	1	3	1	1	1	1	1
1	1	1	1	1	1	1	1	1
1	1	1	1	1	1	1	1	1
1	1	1	1	1	5	1	1	1
1	1	1	6	1	1	1	1	5
1	1	1	1	1	1	1	1	1
1	1	1	1	3	2	1	1	1
1	1	1	1	1	2	8	2	1
1	1	1	1	1	1	1	2	1
1	1	1	2	1	1	2	1	3
2	1	1	1	1	1	1	1	1
1	1	1	1	1	5	1	1	1
1	1	1	1	9	1	1	1	1
1	1	1	1	3	1	1	1	2
1	1	1	1	1	1	3	1	1
1	1	1	1	1	13	1	1	1
1	1	1	4	1	1	1	1	1
1	1	1	1	3	1	1	1	1
1	2	1	1	1	1	1	1	1
1	1	1	2	1	1	1	14	1
1	1	1	1	3	1	4	1	1
1	1	1	1	2	6	1	2	1
1	1	1	1	1	1	1	1	1
1	1	1	1	1	1	1	2	1
1	1	1	8	2	1	1	1	1
1	1	1	1	1	1	7	1	1
2	1	1	1	1	3	1	1	1
1	1	1	1	4	1	1	8	1
1	1	1	1	1	1	1	1	1
1	1	1	5	1	1	1	1	1
1	1	1	1	1	1	1	1	20
1	1	1	1	1	2	3	1	1
1	1	2	2	1	1	1	5	1
1	1	1	1	1	1	1	1	1
1	1	1	1	4	1	1	1	1
1	1	1	1	3	1	1	1	6
1	1	1	1	1	1	1	1	1
1	1	1	2	1	7	1	1	1
1	1	1	1	1	1	1	10	1
1	1	1	3	1	1	1	1	1
1	1	1	1	5	1	2	1	1
1	1	1	1	1	1	1	1	1
1	1	1	4	1	9	1	3	3
1	1	1	1	1	1	1	1	1
1	1	1	1	1	1	1	1	1
1	1	1	1	1	1	1	1	1
2	1	1	4	10	1	1	4	1
1	1	1	1	1	1	1	1	1
1	1	1	1	1	1	3	1	1
1	1	1	1	1	6	1	1	4
1	1	1	1	1	1	1	1	1
1	1	1	1	1	1	1	3	1
1	1	1	3	1	1	1	1	1
1	1	1	1	1	1	1	1	1
1	1	1	1	5	1	1	1	1
1	1	1	1	1	6	1	1	1
1	1	1	1	1	1	1	1	1
1	1	1	1	1	1	2	1	1
1	1	1	1	1	1	2	1	6
1	1	1	1	1	1	2	1	1
1	2	1	1	1	1	3	3	1
1	1	1	1	1	1	1	1	1
1	1	1	11	1	1	1	1	2
1	1	1	1	1	1	1	1	1
1	1	1	1	1	1	1	1	1
1	1	1	1	1	8	2	1	1
1	1	1	3	1	1	1	1	1
1	1	1	6	7	1	1	1	1
1	1	1	1	1	1	1	9	1
1	1	1	1	1	1	1	1	1
1	1	1	1	1	1	1	1	4
1	1	1	1	1	2	1	1	1
1	1	1	1	1	1	1	1	1

Cluster magnitude

24 days Frequency (%)

	WT1	WT2	WT3	P1C1	P1C2	P1C3	P2C1	P2C2	P2C3
1	96.633	96.2963	97.3064	80.11696	80.70175	83.62573	84.21053	82.45614	84.79532
2	2.693603	2.693603	1.683502	5.847953	3.508772	5.263158	5.847953	4.678363	3.508772
3	0.673401	0.673401	1.010101	4.093567	5.847953	1.754386	3.508772	4.678363	2.923977
4	0	0.3367	0	3.508772	4.093567	1.754386	2.339181	1.754386	2.339181
5	0	0	0	1.169591	1.169591	1.169591	0.584795	1.169591	1.754386
6	0	0	0	1.754386	1.169591	2.923977	0	0.584795	1.169591
7	0	0	0	0	0.584795	1.169591	0.584795	0.584795	1.169591
8	0	0	0	1.169591	0	0.584795	1.169591	1.169591	1.169591
9	0	0	0	0.584795	1.754386	1.169591	1.169591	0.584795	0
10	0	0	0	0.584795	0.584795	0	0	1.169591	0
11	0	0	0	0.584795	0	0	0	0	0.584795
12	0	0	0	0	0	0	0	0	0
13	0	0	0	0	0	0.584795	0	0	0
14	0	0	0	0	0	0	0	0.584795	0
15	0	0	0	0	0	0	0	0	0
16	0	0	0	0	0	0	0	0	0
17	0	0	0	0	0	0	0	0	0
18	0	0	0	0.584795	0	0	0	0	0
19	0	0	0	0	0	0	0	0	0
20	0	0	0	0	0	0	0.584795	0	0.584795
24	0	0	0	0	0	0	0	0.584795	0
28	0	0	0	0	0.584795	0	0	0	0

Final data points of the plot

Cluster size	WT			P1C			P2C		
	WT1	WT2	WT3	PC1	PC2	PC3	P2C1	P2C2	P2C3
1	96.633	96.2963	97.3064	80.11696	80.70175	83.62573	84.21053	82.45614	84.79532
2-4	3.367003	3.703704	2.693603	13.45029	13.45029	8.77193	11.69591	11.11111	8.77193
>4	0	0	0	6.432749	5.847953	7.602339	4.093567	6.432749	6.432749

	WT	P1C	P2C
1	96.75±0.3%	81.48±1.09%	83.82±0.7%
2-4	3.25±0.3%	11.89±1.56%	10.53±0.89%
>4	0±0%	6.63±0.52%	5.65±0.78%

Average clone size

WT	P1C	P2C
1.04±0.01	1.7±0.1	1.64±0.1

1	1	1	1	6	1	1	3	1
1	2	1	10	1	1	1	1	1
1	1	1	1	1	2	1	1	1
1	1	1	1	1	1	4	2	8
1	1	1	1	2	1	1	1	3
1	1	1	1	1	1	1	1	1
1	1	1	1	1	1	1	1	1
1	1	1	1	1	1	1	2	1
1	1	1	1	6	1	1	1	1
3	1	1	1	1	1	1	1	1
1	1	1	6	1	1	1	1	1
1	1	1	1	1	3	1	4	1
1	1	1	1	28	1	1	1	1
1	1	1	1	1	1	1	1	1
1	1	1	1	1	1	1	1	2
1	1	1	1	1	1	2	1	1
1	1	2	1	1	1	1	1	1
1	1	1	1	1	1	1	1	1
1	1	1	4	9	1	1	1	1
1	1	1	1	1	1	1	1	7
1	1	1	1	1	1	1	8	5
1	1	1	1	1	4	1	1	1
1	1	1	1	1	1	1	1	1
1	1	1	1	1	1	1	1	1
1	1	1	1	1	1	4	1	1
1	1	1	1	1	1	1	1	1
1	3	1	1	1	1	1	1	1
1	1	1	1	9	1	1	3	1
1	1	1	1	1	1	1	1	1
1	1	1	3	1	1	1	1	5
1	1	1	1	1	1	1	1	1
1	1	1	1	1	1	1	1	1
1	1	1	1	1	1	1	1	1
1	1	1	1	2	1	1	3	1
1	1	1	1	1	1	1	1	1
1	1	1	1	1	1	1	1	1
1	1	1	1	1	1	9	1	4
1	1	2	1	1	1	1	1	1
1	1	1	1	1	1	1	1	1
1	1	1	1	1	1	1	1	1
1	1	1	1	3	1	3	1	1
1	1	1	1	3	2	1	1	1
1	1	1	2	1	1	1	2	1
1	1	1	5	1	1	1	1	1
1	2	1	1	1	1	1	10	1
1	1	1	1	1	1	1	1	1
1	1	1	1	1	1	1	2	7
1	1	1	1	2	1	1	1	1
2	1	1	1	1	1	2	1	1
1	1	1	1	1	1	1	1	1
1	1	1	3	1	1	1	1	1
1	1	1	1	1	1	1	1	1
1	1	1	1	3	4	1	1	1
1	1	1	1	2	1	1	1	1
1	1	1	1	1	1	1	7	1
1	1	1	3	1	1	1	1	1
1	1	1	1	1	1	1	1	1
1	1	1	1	4	1	2	1	1
1	1	1	1	1	1	1	1	1
1	1	1	1	1	1	1	1	1
1	1	1	1	1	1	1	1	11
1	1	1	2	1	1	1	1	1
1	2	1	1	1	1	1	1	1
1	1	1	1	1	9	1	1	1
1	1	1	1	1	1	8	1	1
1	1	1	1	3	1	1	1	1
1	1	1	1	1	1	1	1	2
1	1	1	2	1	1	1	24	1
1	1	1	1	1	1	1	1	1
1	1	3	1	1	1	1	1	1
1	1	1						
1	1	1						
1	1	1						
1	1	1						
1	1	1						
1	1	1						
1	1	1						
1	1	1						
1	1	1						
2	1	1						
1	1	1						
1	1	1						
1	1	1						
1	1	1						
1	1	1						
1	1	1						
1	1	1						
1	2	1						
1	1	1						
1	1	1						
1	1	1						
1	1	1						
1	1	1						
1	1	1						
1	1	1						
1	1	1						
1	1	1						
1	1	1						
1	1	1						
1	1	1						
1	1	2						
1	1	1						
1	1	1						
1	1	1						
1	1	1						

1	1	1		
2	1	1		
1	1	1		
1	1	1		
1	1	1		
1	1	1		
1	1	1		
1	1	1		
1	2	1		
1	1	1		
1	1	1		
1	1	1		
1	1	1		
1	1	1		
1	1	1		
1	1	1		
1	1	1		
1	1	1		
1	1	1		
1	1	1		
1	1	1		
1	1	1		
1	1	1		
1	1	2		
1	1	1		
1	1	1		
1	1	1		
1	1	1		
1	1	1		
1	1	1		
1	1	1		
1	1	1		
1	1	1		
3	1	1		
1	1	1		
1	1	1		
1	1	1		
1	1	1		
1	1	1		
1	1	1		
1	1	1		
1	1	1		
1	1	1		
1	1	1		
1	1	1		
1	1	1		
1	1	1		
1	1	1		
1	1	1		
1	1	1		
1	1	1		
1	1	1		
1	1	1		
1	1	1		
1	1	1		
1	1	1		
1	1	1		
1	1	1		
1	4	1		
1	1	1		
1	1	1		
1	1	1		
1	1	1		
1	1	1		
1	1	1		
1	1	1		
1	1	1		
1	1	1		
1	1	1		
1	1	3		
1	1	1		
1	1	1		
1	1	1		
2	1	1		
1	1	1		
1	1	1		
1	1	1		
1	1	1		
1	1	1		
1	1	1		
1	1	1		
1	1	1		
1	1	1		
1	1	1		
1	1	1		
1	3	1		
1	1	1		
1	1	1		
1	1	1		
1	1	1		
1	1	1		

Figure 2D.3F

2 months																	
WT			IFT88f/f CAG			PKD1 f/f CAG(1)			IFT88f/f PKD1 f/f CAG			PKD2 f/f CAG			PKD1 f/f CAG(2)		
WT1	WT2	WT3	IC1	IC2	IC3	P1C1	P1C2	P1C3	IPC1	IPC2	IPC3	P2C1	P2C2	P2C3	P1C1	P1C2	P1C3
1	1	1	1	1	1	3	1	1	1	1	1	3	1	2	1	3	6
1	1	1	1	1	2	1	2	1	1	1	1	2	5	2	3	1	3
1	1	1	1	1	1	9	1	3	1	2	1	1	1	1	1	2	1
1	1	1	1	1	1	1	3	1	1	1	1	2	2	4	1	1	2
1	1	1	1	1	1	1	1	1	1	1	1	1	3	1	2	1	1
1	1	1	1	1	1	1	4	3	1	1	1	1	2	1	1	4	3
1	1	1	1	1	1	1	1	1	1	1	1	2	1	6	1	3	1
1	1	1	1	1	1	2	1	4	1	1	1	2	1	5	2	1	1
1	1	1	1	1	1	3	3	2	1	2	1	2	2	1	1	2	3
1	1	1	1	1	1	1	1	1	1	1	1	1	4	1	1	1	1
1	1	1	1	2	1	1	2	3	1	1	1	1	1	2	3	2	2
1	1	1	1	1	1	1	1	1	1	1	1	1	1	2	2	1	1
1	1	2	1	1	1	5	2	1	1	1	1	2	1	3	1	8	1
2	1	1	1	1	1	1	1	2	1	1	1	4	2	1	1	1	3
1	1	1	2	1	1	1	2	1	1	1	1	2	1	5	1	2	1
1	1	1	1	1	1	3	1	3	2	1	1	1	1	1	4	1	2
1	1	1	1	1	1	1	2	1	1	1	1	3	3	1	1	1	1
1	1	1	1	1	1	1	1	1	1	1	2	1	1	1	1	3	1
1	1	1	1	1	1	2	2	4	1	1	1	3	1	3	2	1	2
1	1	1	1	1	1	1	1	2	1	1	1	1	1	3	1	2	1
1	1	1	1	1	1	4	3	1	1	1	1	4	1	1	1	1	6
1	1	1	1	1	1	1	1	2	1	1	1	1	2	4	2	1	1
1	1	1	1	1	1	1	3	1	1	1	1	2	2	1	1	4	1
1	1	1	1	1	1	2	1	1	1	1	1	1	2	2	4	1	2
1	1	1	1	1	1	1	1	3	1	1	1	4	2	1	3	1	1
1	2	1	1	1	1	3	1	4	1	1	1	1	3	1	1	4	1
1	1	1	1	1	1	3	1	4	1	1	1	1	1	1	3	1	4
1	1	2	1	1	1	1	2	1	1	1	1	5	1	1	1	2	1
1	1	1	1	1	1	2	3	3	1	1	1	1	1	1	1	1	1
1	1	1	1	1	1	1	5	1	1	1	1	1	2	1	3	1	3
1	1	1	1	1	1	1	1	4	1	1	2	1	2	1	1	2	1
1	1	1	1	1	1	3	1	3	1	1	1	1	3	2	2	1	1
1	1	1	1	2	1	2	2	2	1	1	1	1	3	2	1	3	3
1	1	1	1	1	1	1	1	1	1	1	1	2	2	2	1	1	2
1	1	1	1	1	1	1	2	1	2	1	1	1	1	1	1	2	1
1	1	1	1	1	1	1	1	2	1	1	1	1	4	1	3	2	1
1	1	1	1	1	1	2	1	1	1	1	1	1	1	1	3	1	5
1	1	1	1	1	1	2	1	2	1	3	1	2	1	1	1	1	1
1	1	1	1	1	1	1	1	1	1	1	1	1	1	1	1	3	1
2	1	1	2	1	1	3	2	3	1	1	2	3	2	4	1	1	1
1	1	1	2	1	1	1	1	1	1	1	1	2	2	1	3	1	6
1	1	1	1	1	1	1	1	4	1	1	1	2	1	1	1	3	4
1	1	2	1	1	1	5	3	1	1	2	1	1	6	2	2	1	2
1	1	1	1	1	1	1	1	3	1	1	1	1	1	2	1	2	1
1	1	1	1	1	1	2	1	1	3	1	1	1	1	2	4	1	3
1	2	1	1	3	1	1	3	1	1	1	1	1	1	3	2	3	1
1	1	1	1	1	1	3	2	3	1	1	1	2	1	2	6	2	1
1	1	1	1	1	1	2	1	1	1	1	1	1	3	2	1	1	2
1	1	1	1	1	2	1	1	1	1	1	1	2	2	1	1	4	2
1	1	1	1	1	1	2	3	2	1	1	1	2	1	4	2	1	1
1	1	1	1	1	1	1	2	2	1	1	1	2	2	3	1	1	3
2	1	1	1	1	1	1	1	1	1	1	1	1	2	3	1	2	1
1	1	1	1	1	1	6	4	2	1	1	1	3	4	1	4	1	1
1	1	1	1	1	1	1	1	1	1	1	1	3	1	1	1	3	1
1	1	1	1	1	1	3	2	3	1	1	1	1	1	1	2	1	5
1	1	1	1	1	1	1	1	1	1	1	1	1	1	1	3	1	1
1	1	1	1	1	1	1	5	2	1	1	1	4	2	1	1	3	1
1	1	2	1	1	1	4	4	1	1	1	1	1	2	3	1	1	1
1	1	1	1	1	1	3	1	3	1	1	1	1	2	6	1	1	3
1	1	1	1	1	1	2	1	1	1	1	1	1	1	2	5	2	1
1	1	1	1	1	1	3	1	1	1	1	1	2	1	1	6	1	1
1	1	1	1	1	1	2	1	2	1	1	1	2	3	1	1	1	2
1	1	1	1	1	1	1	1	2	1	1	1	3	1	2	1	1	2
1	1	1	4	1	1	3	4	1	1	1	2	1	3	1	3	2	3
1	1	1	1	1	1	1	2	3	1	1	1	1	4	1	1	1	1
1	1	1	1	1	1	3	2	1	1	1	1	3	1	1	1	3	2
1	1	1	1	2	3	1	1	1	1	1	1	1	1	1	2	1	1
1	1	1	1	1	1	4	1	2	2	3	1	1	1	3	1	1	2
1	1	1	1	1	1	2	1	1	1	1	1	2	1	3	1	3	1
1	1	1	1	1	1	1	3	1	1	1	1	1	2	1	1	1	1
2	1	1	1	1	1	3	1	3	1	1	1	1	1	1	4	1	1
1	1	1	1	1	1	1	4	1	1	1	1	1	1	2	1	2	3
1	1	2	1	1	1	2	2	1	1	2	1	1	2	1	1	1	1
1	1	1	1	1	1	5	1	3	1	1	1	1	1	1	5	1	1
1	1	1	1	1	1	1	2	1	1	1	1	3	1	1	2	6	1
1	1	1	1	1	1	1	1	3	1	1	1	2	2	2	1	1	3
1	1	1	1	1	1	1	2	1	1	1	1	4	1	2	1	2	1
1	1	1	1	1	1	2	1	2	1	1	1	2	2	2	7	1	1
1	1	1	1	1	1	3	1	1	1	1	1	1	1	1	1	1	1
1	1	1	1	1	1	1	2	1	1	1	1	1	1	1	1	4	5
1	1	1	1	1	1	2	1	3	1	1	1	2	3	1	1	1	1
1	1	1	2	1	1	1	2	2	1	1	1	3	1	4	6	2	3
1	1	1	1	1	1	2	4	1	1	1	1	1	1	1	2	1	1
1	1	1	1	1	1	1	3	1	1	1	1	1	2	1	1	2	1
1	2	1	1	1	1	3	1	2	1	1	1	1	1	1	2	1	2
1	1	1	1	1	1	2	1	3	1	3	1	1	1	1	1	1	6
1	1	1	1	1	1	1	3	1	1	1	1	2	1	2	1	1	1
1	1	1	1	1	1	4	1	1	1	1	1	2	5	1	2	2	1
1	1	1	1	1	1	1	6	3	1	1	1	2	1	1	1	1	3
1	1	1	1	1	1	2	3	1	1	1	1	4	1	2	1	1	1
1	1	1	1	1	2	1	1	1	1	1	1	3	2	1	1	2	5
1	1	1	1	1	1	1	1	2	1	1	2	1	1	1	3	3	1
1	1	1	1	1	1	3	3	1	1	1	1	1	4	1	1	1	1
1	1	1	1	1	1	1	1	1	1	1	1	1	1	2	1	2	6
1	1	1	1	1	1	3	2	1	1	1	1	1	1	2	1	1	1
1	1	2	1	1	1	3	1	3	1	1	1	2	2	1	4	1	1
1	1	1	1	1	1	1	1	1	1	1	1	3	2	1	1	5	3
1	1	1	1	1	1	1	2	1	2	1	1	2	1	4	1	1	1
1	1	1	1	1	1	1	1	1	1	1	1	2	1	1	3	3	1
1	1	1	1	1	1	1	2	2	1	1	1	2	1	1	1	1	1
1	1	1	1	1	1	1	2	1	1	1	1	1	1	3	1	1	8
1	1	1	2	1	1	1	1	1	1	1	1	1	3	8	1	1	1
1	1	1	1	1	1	1	1	6	1	1	1	3	1	1	4	3	1
1	1	1	1	1	1	2	2	1	1	1	1	2	1	1	1	1	4
1	1	1	1	1	3	1	2	2	1	1	1	1	1	3	1	1	1
1	1	1	1	1	1	2	1	1	1	1	1	2	1	1	1	2	1
1	1	1	1	1	1	2	3	3	1	2	1	1	2	1	3	3	1
2	1	1	1	1	1	1	1	1	1	1	2	1	3	1	1	1	2
1	1	1	1	1	1	3	1	1	1	1	1	1	1	3	2	1	1
1	1	1	1	1	1	1	2	2	1	1	1	2	1	1	1	2	1
1	1	1	1	1	1	1	4	1	1	1	1	3	1	1	1	1	1
1	1	1	1	1	1	3	1	3	1	1	1	1	4	1	5	1	3
1	3	1	2	3	1	3	1	1	1	1	1	1	1	2	1	1	1
1	1	1	1	1	1	1	1	1	1	1	1	1	1	2	1	8	1
1	1	1	1	1	1	4	2	3	2	1	1	1	2	1	3	1	1
1	1	1	1	1	1	1	1	1	1	1	1	3	1	1	1	2	1
1	1	1	1	1	1	1	2	1	1	1	1	1	1	1	1	1	2
1	1	1	1	1	1	3	3	2	1	1	1	1	2	4	4	1	1
1	1	1	1	1	1	1	1	1	1	1	1	2	2	1	2	1	1
1	1	1	1	1	1	4	1	1	1	1	1	2	1	1	1	2	1
1	1	1	1	1	1	1	1	1	1	1	1	1	1	2	1	1	1
1	1	1	1	1	1	1	1	3	1	1	1	1	1	1	1	1	3
1	1	1	1	1	1	2	1	1	1	1	1	2	3	1	1	5	2
1	1	1	1	1	1	4	1	1	1	1	1	1	1	1	5	1	1
1	1	2	1	1	1	1	1	2	1	1	1	1	5	2	1	3	4
1	1	1	1	2	1	2	1	1	1	1	1	3	1	2	1	1	2
1	1	1	1	1	1	1	2	3	1	1	1	1	1	2	3	1	1
1	1	1	2	1	1	3	1	1	1	1	1	1	1	1	1	5	1
1	1	1	1	1	1	1	3	1	1	1	1	4	2	1	2	8	1
1	1	1	1	1	1	1	2	2	1	1	1	1	1	1	1	1	3
1	1	1	1	1	1	1	2	1	1	1	1	1	1	2	1	6	2
1	1	1	1	1	2	1	1	5	1	1	1	1	3	1	2	1	1
1	1	1	1	1	1	1	2	1	1	1	1	2	3	1	5	1	2
1	1	1	1	1	1	2	1	2	1	2	1	1	1	3	1	2	1
1	1	1	1	1	1	1	2	1	1	1	1	1	2	1	1	1	1
1	1	1	1	1	1	2	1	3	1	1	1	1	1	1	3	1	4
1	1	1	1	1	1	3	1	1	1	1	1	1	2	1	1	2	2
1	1	1	2	1	1	1	2	4	1	1	1	1	5	1	2	1	1
1	1	1	1	1	1	1	1	1	1	1	1	1	1	2	1	3	1
1	1	2	1	1	1	3	1	3	1	1	1	1	1	1	1	1	5
1	1	1	1	1	1	1	3	1	1	1	1	1	1	1	1	3	1
1	1	1	1	1	1	2	3	1	1	1	1	1	1	1	1	1	1
1	1	1	1	1	1	1	1	1	1	1	1	3	2	5	4	1	2
1	1	1	1	1	2	1	1	1	1	1	1	1	2	3	3	2	1
1	1	1	1	1	1	1	2	2	1	1	1	1	1	1	1	1	1
1	1	1	1	1	1	6	1	3	1	1	1	1	3	1	1	1	7
2	1	1	1	1	1	1	3	1	1	1	1	1	1	2	1	5	1
1	1	1	1	1	1	1	4	1	1	1	2	2	1	1	6	1	2
1	1	1	1	1	1	1	1	2	1	1	1	1	1	1	1	1	1
1	1	1	1	1	1	1	2	1	3	1	1	1	1	1	1	2	1
1	1	1	1	2	1	1	4	1	1	1	1	1	1	1	3	1	1
1	1	1	1	1	1	2	2	3	1	1	1	1	2	2	1	1	2
1	1	1	1	1	1	1	3	3	1	1	1	3	1	3	2	5	1
1	1	1	1	1	1	2	2	1	1	1	1	1	1	2	1	1	1
1	1	1	1	1	1	1	3	2	1	1	1	1	1	1	6	1	1
1	2	1	2	1	1	3	1	2	1	1	1	1	1	1	2	2	3
1	1	1	1	1	1	1	2	1	1	2	1	1	3	1	1	1	3
1	1	2	1	1	1	3	1	3	1	1	1	1	1	2	3	4	1
1	1	1	1	1	1	1	1	1	1	1	1	3	1	1	1	1	2
1	1	1	1	1	1	4	2	1	1	1	1	2	1	1	1	3	1
1	1	1	1	1	1	2	1	3	1	1	1	1	6	2	1	1	1
1	1	1	1	1	1	1	2	1	1	1	1	1	2	1	5	1	2
1	1	1	1	1	1	2	4	2	1	1	1	1	1	1	1	2	1
1	1	1	1	1	2	1	1	1	1	1	1	1	1	1	3	1	1
1	1	1	1	1	1	3	1	1	1	1	1	5	2	3	1	4	3
1	1	1	1	3	1	1	2	5	1	1	1	1	1	1	2	1	1
1	1	1	1	1	1	2	3	1	1	1	1	1	1	1	1	1	5
1	1	1	1	1	1	1	1	1	1	1	1	1	2	1	1	3	1
1	1	1	1	1	1	1	3	1	1	1	1	2	1	1	1	1	1
1	1	1	1	1	1	1	1	5	1	1	1	2	1	5	2	1	6
1	1	1	1	1	1	1	3	2	2	1	1	3	1	1	1	1	1
1	1	1	1	1	1				1	1	1						
1	1	1	1	1	1				1	1	1						
1	1	1	1	1	1				1	1	1						
1	1	1	1	1	1				1	1	1						
2	1	1	1	1	1				1	1	1						
1	1	3	1	1	1				1	1	2						
1	1	1	1	1	1				1	1	1						
1	1	1	1	1	1				1	1	1						
1	1	1	1	1	1				1	1	1						
1	1	1	1	1	1				1	1	1						
1	1	1	2	1	1				1	1	1						
1	1	1	1	1	1				1	1	1						
1	1	1	1	1	1				1	1	1						
1	2	1	1	1	1				1	1	1						
1	1	1	1	2	1				1	1	1						
1	1	1	1	1	1				1	1	1						
1	1	1	1	1	1				1	1	1						
1	1	1	1	1	1				1	1	1						
3	1	1	1	1	1				1	1	1						
1	1	1	2	1	3				1	1	1						
1	1	1	1	1	1				1	1	1						
1	1	1	1	1	1				2	1	1						

Cluster magnitude

2 months Frequency (%)

	WT1	WT2	WT3	IC1	IC2	IC3	P1C1(1)	P1C2(1)	P1C3(1)	IPC1	IPC2	IPC3	P2C1	P2C2	P2C3	P1C1(2)	P1C2(2)	P1C3(2)
1	95.6229	96.633	95.2862	94.2761	95.6229	95.9596	56.1404	53.2164	56.7251	95.9596	95.2862	95.6229	59.6491	60.2339	61.4035	61.9883	60.2339	61.4035
2	3.367	2.6936	4.0404	4.7138	2.693603	3.0303	18.7135	25.1462	18.1287	3.0303	3.0303	3.7037	23.3918	23.3918	21.0526	14.0351	18.1287	15.2047
3	0.6734	0.6734	0.6734	0.6734	1.683502	1.0101	16.9591	14.0351	18.7135	0.6734	1.3468	0.6734	11.6959	9.35673	9.35673	11.6959	11.6959	12.2807
4	0.3367	0	0	0.3367	0	0	4.67836	5.84795	4.09357	0.3367	0.3367	0	4.09357	3.50877	4.09357	5.26316	4.09357	2.92398
5	0	0	0	0	0	0	1.75439	1.16959	1.75439	0	0	0	1.16959	2.33918	2.33918	3.50877	2.92398	3.50877
6	0	0	0	0	0	0	1.16959	0.5848	0.5848	0	0	0	0	1.16959	1.16959	2.92398	1.16959	3.50877
7	0	0	0	0	0	0	0	0	0	0	0	0	0	0	0	0.5848	0	0.5848
8	0	0	0	0	0	0	0	0	0	0	0	0	0	0	0.5848	0	1.75439	0.5848
9	0	0	0	0	0	0	0.5848	0	0	0	0	0	0	0	0	0	0	0

Final data points of the plot

Cluster size	WT			IC			P1C(1)			IPC			P2C			P1C(2)		
	WT1	WT2	WT3	IC1	IC2	IC3	PC1	PC2	PC3	IPC1	IPC2	IPC3	P2C1	P2C2	P2C3	PC1	PC2	PC3
1	95.6229	96.633	95.2862	94.2761	95.6229	95.9596	56.1404	53.2164	56.7251	95.9596	95.2862	95.6229	59.6491	60.2339	61.4035	61.9883	60.2339	61.4035
2-4	4.3771	3.367	4.7138	5.72391	4.377104	4.0404	40.3509	45.0292	40.9357	4.0404	4.7138	4.3771	39.1813	36.2573	34.5029	30.9942	33.9181	30.4094
>4	0	0	0	0	0	0	3.50877	1.75439	2.33918	0	0	0	1.16959	3.50877	4.09357	7.01754	5.84795	8.18713

	WT	IC	P1C(1)	IPC	P2C	P1C(2)
1	95.85±0.4%	95.29±0.51%	55.36±1.09%	95.62±0.19%	60.43±0.52%	61.21±0.52%
2-4	4.15±0.4%	4.71±0.51%	42.11±1.47%	4.38±0.19%	36.65±1.36%	31.77±1.09%
>4	0±0%	0±0%	2.53±0.52%	0±0%	2.92±0.89%	7.02±0.48%

Average clone size

WT	IC	P1C(1)	IPC	P2C	P1C(2)
1.05±0.01	1.06±0.01	1.8±0.05	1.06±0.01	1.68±0.05	1.86±0.06

1	1	1	1	1	1		1	1	1		
1	1	1	1	1	1		1	1	1		
1	1	1	1	1	1		1	1	1		
1	1	1	1	1	1		1	1	1		
1	1	1	1	1	1		1	1	1		
1	1	1	1	1	1		1	1	2		
1	1	1	1	1	1		1	1	1		
1	1	1	1	1	1		1	1	1		
1	1	1	1	1	1		1	1	1		
1	1	1	1	1	1		1	1	1		
1	1	1	1	1	1		1	1	1		
1	1	1	1	1	1		1	1	1		
1	1	1	1	2	1		1	1	1		
1	1	1	1	1	1		1	1	1		
1	1	1	1	1	1		1	1	1		
1	1	1	1	1	1		1	1	1		
1	1	1	1	1	2		1	1	1		
1	1	1	1	1	1		1	1	1		
1	1	1	1	1	1		1	2	1		
1	1	1	1	1	1		1	1	1		
4	1	3	1	3	1		1	1	1		
1	1	1	1	1	1		1	1	1		
1	1	1	1	1	1		1	1	1		
1	1	1	1	1	1		1	1	1		
1	1	1	3	1	1		1	1	1		
1	3	1	1	1	1		1	1	1		
1	1	1	1	1	1		2	1	1		
1	1	1	1	1	1		1	1	1		
1	1	1	1	1	1		1	1	1		
1	1	1	1	1	1		1	1	1		
2	1	1	1	1	1		1	1	1		
1	1	1	1	1	1		1	1	1		
1	1	1	1	1	1		1	1	1		
1	1	1	1	1	1		1	1	1		
1	1	1	1	1	1		1	1	1		
1	1	1	1	1	1		1	1	1		
1	1	1	1	1	1		1	1	3		
1	1	1	1	1	1		1	1	1		
1	1	1	1	1	1		1	1	1		
1	1	1	1	1	1		1	1	1		
1	1	1	2	1	1		1	1	1		
1	1	1	1	1	1		1	1	1		
1	1	1	1	1	1		1	1	1		
1	1	1	1	1	1		1	1	1		
1	1	1	1	1	1		1	1	1		
1	1	2	1	1	1		1	1	1		
1	1	1	1	2	1		1	1	1		
1	1	1	1	1	1		1	1	1		
1	1	1	1	1	1		1	1	1		
1	1	1	1	1	1		1	1	1		
1	1	1	1	1	1		1	1	1		
1	1	1	1	1	1		1	1	1		
1	1	1	1	1	1		1	1	1		
3	1	1	1	1	1		1	1	1		
1	1	1	1	1	1		1	1	1		
1	1	1	1	1	1		1	1	1		
1	1	1	1	1	1		1	1	2		
1	1	1	1	1	1		1	1	1		
1	1	1	1	1	2		1	1	1		
1	1	1	1	1	1		1	1	1		
1	1	1	1	1	1		1	1	1		
1	2	1	1	1	1		1	1	1		
1	1	1	1	1	1		1	1	1		
1	1	1	1	1	1		1	1	1		
1	1	1	1	1	1		1	1	1		
1	1	1	1	1	1		1	1	1		
1	1	1	1	1	1		1	1	1		
1	1	2	1	1	1		1	3	1		
1	1	1	1	1	1		1	1	1		
1	1	1	1	1	1		1	1	1		
2	1	1	1	1	1		1	1	1		
1	1	1	1	1	1		1	1	1		
1	1	1	1	1	1		1	1	1		
1	1	1	3	1	1		1	1	1		
1	1	1	1	1	1		1	1	1		
1	1	1	1	1	1		1	1	1		
1	2	1	1	1	1		1	1	1		
1	1	1	1	1	1		1	1	1		
1	1	1	1	1	1		1	1	1		
1	1	1	1	1	1		4	2	3		
1	1	1	1	1	1		1	1	1		
1	1	1	1	1	1		1	1	1		
2	1	1	1	1	1		1	1	1		
1	1	1	1	1	1		1	1	1		
1	1	1	1	3	1		1	1	1		
1	1	1	1	1	1		1	1	1		
1	1	1	1	1	1		1	1	1		
1	2	1	2	1	1		1	1	1		
1	1	1	1	1	1		1	1	1		
1	1	2	1	1	1		1	1	1		
1	1	1	1	1	1		1	1	1		
1	1	1	1	1	2		1	1	1		
1	1	1	1	1	1		2	1	1		
1	1	1	1	1	1		1	1	1		
1	1	1	1	1	1		1	1	1		
1	1	1	1	1	1		1	1	1		
1	1	1	2	1	1		1	1	1		
1	1	1	1	1	1		1	4	1		
1	1	1	1	1	1		1	1	1		
1	1	1	1	1	1		1	1	1		
1	1	1	1	1	1		1	1	1		
1	1	1	1	1	1		1	1	1		
1	1	1	1	1	1		1	1	2		
1	1	1	1	1	1		1	1	1		

Figure 2F.3H

4 months																	
WT			IFT88f/f CAG			PKD1 f/f CAG(1)			IFT88f/f PKD1 f/f CAG			PKD2 f/f CAG			PKD1 f/f CAG(2)		
WT1	WT2	WT3	IC1	IC2	IC3	P1C1	P1C2	P1C3	IPC1	IPC2	IPC3	P2C1	P2C2	P2C3	P1C1	P1C2	P1C3
1	1	1	1	1	1	26	1	10	1	1	1	2	9	5	1	7	4
1	1	1	1	1	1	3	1	6	1	1	6	2	3	2	32	3	1
1	1	1	1	1	1	2	22	1	1	1	1	16	1	4	6	4	9
1	1	1	1	1	1	1	2	1	1	1	1	2	1	4	1	1	11
1	1	1	1	1	1	3	1	3	1	4	1	1	10	6	1	24	1
1	1	1	1	1	1	4	1	1	1	1	1	1	2	1	10	6	7
3	1	1	1	1	1	1	17	7	1	1	1	36	1	1	2	1	1
1	1	1	1	1	1	7	8	4	1	1	1	5	8	17	1	2	2
1	1	1	1	1	1	4	1	1	5	1	1	1	1	11	3	1	1
1	1	1	1	1	1	5	1	1	1	1	1	2	5	9	1	18	4
1	1	1	1	1	1	1	1	3	1	1	1	9	2	1	1	1	1
1	1	2	1	1	1	3	1	9	1	1	1	11	1	1	16	1	3
1	2	1	1	1	1	1	3	1	1	1	3	1	37	5	6	5	1
1	1	1	1	1	1	16	1	6	2	1	1	1	1	1	1	2	15
1	1	2	1	1	1	1	4	1	1	3	1	4	3	1	5	1	4
1	1	1	1	1	1	3	1	12	1	1	1	22	1	8	1	2	1
1	1	1	1	1	2	4	6	5	1	1	1	4	1	1	4	1	7
1	1	1	1	1	1	1	6	3	1	1	1	5	12	1	2	6	1
1	1	1	1	1	1	6	1	3	1	1	1	1	3	1	1	1	4
1	1	1	1	1	1	12	1	11	1	1	1	1	6	3	12	2	9
1	1	1	1	1	1	1	8	4	6	1	1	3	3	20	1	2	1
2	1	1	1	1	1	6	9	1	1	1	1	1	2	1	1	8	30
1	1	1	1	1	2	3	7	2	1	1	1	8	16	1	2	17	1
1	1	1	1	1	1	7	1	4	1	1	1	3	8	5	1	1	14
1	1	2	1	1	1	3	6	5	1	2	1	2	1	7	8	1	7
1	1	1	1	2	1	2	1	1	1	1	1	1	1	32	1	3	1
1	1	1	1	1	1	1	2	1	1	1	1	11	2	1	3	1	5
1	1	1	1	1	1	13	1	6	1	1	1	6	5	11	11	1	6
1	1	1	1	1	1	1	2	9	1	1	1	16	7	6	1	29	12
1	1	1	2	1	1	3	2	4	1	1	1	4	1	4	6	1	1
1	1	1	1	1	1	1	1	6	2	1	1	1	3	9	1	8	3
1	1	1	1	1	1	1	1	1	1	1	1	1	3	2	4	1	3
1	3	1	1	1	1	1	9	7	1	2	4	1	17	5	16	4	1
1	2	1	1	1	1	16	7	3	1	1	1	3	4	3	1	1	2
1	1	1	1	1	1	11	29	5	1	1	1	5	1	8	8	3	16
1	1	1	1	1	1	1	30	1	1	1	1	2	1	10	10	7	1
1	2	1	1	1	1	3	4	7	1	1	1	1	2	1	2	4	1
1	1	1	1	1	1	1	1	35	1	1	1	2	14	12	4	8	4
1	1	1	1	1	1	2	6	9	1	3	1	1	5	2	1	1	1
1	1	1	1	1	1	23	2	1	2	1	1	8	1	2	2	5	9
1	1	1	1	1	1	16	1	2	1	1	2	24	1	1	2	1	1
1	1	1	1	1	1	3	6	4	1	1	1	4	1	2	15	3	37
1	1	1	1	1	1	12	1	4	1	1	1	1	11	1	2	2	26
1	1	1	1	1	1	1	1	3	1	1	1	16	30	19	3	1	1
1	1	1	1	1	1	3	8	1	1	1	2	5	1	8	1	2	3
1	2	1	1	1	1	1	6	1	1	5	1	3	2	32	2	4	1
1	1	1	5	1	1	20	15	1	1	1	1	2	2	1	2	6	5
1	1	1	1	1	1	7	3	4	3	1	1	6	3	3	1	1	1
1	1	1	1	1	1	3	3	15	1	1	1	5	3	3	21	18	19
1	1	1	1	1	1	11	2	6	1	1	1	1	3	28	2	6	1
1	1	1	1	1	1	4	3	1	1	1	1	1	1	2	1	4	5
1	1	1	1	1	1	6	10	7	1	3	1	1	3	4	6	1	1
1	1	1	2	1	1	13	7	3	1	1	1	7	5	1	1	10	6
1	1	1	1	1	1	23	6	21	1	1	1	3	1	1	2	1	4
1	1	1	1	1	1	2	1	3	1	1	1	2	5	13	1	3	15
1	2	1	1	1	1	9	1	17	1	1	1	2	8	3	1	1	5
3	1	1	1	1	1	1	1	1	1	2	1	1	23	1	7	9	4
1	1	1	1	1	1	1	5	1	1	1	1	1	3	1	1	4	1
1	1	1	1	1	1	2	1	3	1	1	1	1	6	1	5	1	1
1	1	1	1	1	1	1	1	3	1	1	1	15	1	5	14	3	19
1	1	1	1	1	1	6	16	2	4	1	1	4	1	2	1	3	1
1	1	1	2	1	1	1	4	1	1	1	1	8	11	9	3	23	2
1	1	2	1	1	1	2	6	1	1	1	3	2	15	10	1	1	1
1	1	1	1	1	1	3	8	1	1	3	1	18	2	1	3	30	3
1	1	1	1	1	1	3	4	9	2	1	1	4	4	1	1	2	1
1	2	1	1	1	1	7	7	2	1	1	1	8	1	2	3	1	1
1	1	1	1	2	1	3	6	12	1	1	1	6	1	21	4	5	10
1	1	1	1	1	1	2	1	7	2	1	1	7	1	1	12	8	11
1	1	1	1	1	1	3	17	1	1	1	1	1	2	1	8	1	1
1	1	1	1	1	1	7	4	2	1	1	1	38	1	13	2	1	1
1	1	1	4	1	1	4	3	6	1	1	1	4	4	3	1	2	13
1	1	3	1	1	1	2	4	1	1	1	1	4	1	18	3	3	1
1	1	1	1	1	2	1	3	1	1	1	1	1	1	1	1	1	2
1	1	1	1	1	1	13	1	10	1	1	1	3	12	3	21	4	4
1	1	1	1	1	1	9	1	4	1	1	2	2	7	3	27	19	6
1	1	1	1	1	1	10	1	3	1	1	1	1	5	25	16	1	1
2	1	1	1	1	1	1	1	3	1	1	1	1	1	4	1	5	3
1	1	1	1	1	1	1	18	1	1	1	1	8	10	3	1	33	6
1	2	1	1	1	1	1	2	1	1	2	1	1	1	1	7	2	9
1	1	1	1	1	1	5	2	24	2	1	1	1	2	1	2	1	1
1	1	1	1	1	1	2	2	20	1	1	1	11	3	1	1	6	2
1	1	1	1	1	1	1	7	4	1	1	1	1	1	1	3	5	7
1	1	1	1	1	1	31	4	15	1	1	1	3	14	9	2	9	1
1	1	1	1	1	1	4	4	7	1	1	1	7	29	9	19	2	26
2	1	1	1	1	1	7	1	8	1	1	1	3	1	14	1	1	4
1	1	1	1	1	1	1	3	3	1	1	3	2	1	12	28	2	1
1	1	2	1	1	1	6	3	1	1	1	1	1	6	3	1	1	1
1	1	1	1	1	1	2	4	1	1	1	1	1	1	16	2	1	2
1	1	1	1	1	1	1	23	1	1	1	1	3	3	8	5	6	2
2	1	1	1	1	1	1	2	8	1	3	1	3	13	5	3	25	1
1	1	1	1	1	1	14	3	1	1	1	1	4	1	6	13	1	2
1	1	1	1	1	1	15	1	27	1	1	1	5	1	1	2	4	8
1	1	2	2	1	1	22	1	11	1	1	2	3	9	30	4	1	3
1	1	1	1	1	1	1	5	1	4	1	1	8	1	1	18	13	2
1	1	1	1	1	1	1	6	1	1	1	1	14	1	2	1	1	1
1	1	1	1	1	1	1	2	6	1	1	1	1	3	2	1	5	2
1	2	1	1	1	1	4	1	3	1	1	1	1	20	6	20	1	9
1	1	1	1	1	1				1	1	1						
1	1	1	1	1	1				1	1	1						
1	1	1	2	1	1				1	1	1						
1	1	1	1	1	1				1	1	1						
2	1	1	1	1	1				1	1	1						
1	1	1	1	1	1				1	1	1						
1	1	1	1	1	1				1	1	2						
1	1	1	1	1	1				1	1	1						
1	1	1	1	1	1				5	1	1						
1	1	1	1	4	1				1	1	1						
1	1	1	1	1	1				1	4	1						
1	1	1	1	1	1				1	1	1						
1	1	1	1	1	1				1	1	1						
1	1	1	1	1	1				1	1	1						
1	1	1	2	1	1				1	1	1						
1	1	1	1	1	1				1	1	1						
1	1	1	1	1	2				1	1	1						
1	1	1	1	1	1				1	1	1						
1	1	1	1	1	1				2	1	2						
1	1	1	1	1	1				1	1	1						
1	1	1	1	1	1				1	1	1						
1	1	1	1	1	1				1	1	1						
2	1	1	1	1	1				1	1	1						
1	1	1	1	1	1				1	1	1						
1	1	1	1	1	1				1	1	1						
1	1	1	1	1	1				1	1	1						
1	1	1	1	1	1				1	1	1						
1	1	1	1	1	1				4	1	1						
1	1	1	1	1	1				1	1	1						
1	1	1	1	1	1				1	1	1						
1	1	1	1	1	1				1	1	1						
1	1	1	2	1	1				1	1	1						
1	1	1	1	3	1				1	1	1						
2	1	1	1	1	1				1	1	1						
1	1	1	1	1	1				1	1	1						
1	1	1	1	1	1				1	1	1						
1	1	1	1	1	1				1	1	1						
1	1	1	1	1	1				1	1	1						
1	1	1	1	1	2				3	1	1						
1	1	1	1	1	1				1	1	1						
1	1	1	1	1	1				1	1	1						
2	1	1	3	1	1				1	1	1						
1	2	1	1	1	1				1	1	3						
1	1	1	1	1	1				1	1	1						
1	1	1	1	1	2				1	2	1						
1	1	1	1	1	1				1	1	1						
1	1	1	1	1	1				3	1	1						
1	1	1	1	1	1				1	1	1						
1	1	1	1	1	1				1	1	1						
1	1	1	1	1	1				1	1	1						
2	1	1	1	1	1				1	1	1						
1	1	1	1	1	1				1	1	1						
1	1	1	1	1	1				1	1	1						
1	1	1	1	1	1				1	1	1						
1	1	1	1	1	1				2	1	1						
1	1	1	1	1	1				1	1	1						
1	1	1	1	1	1				1	1	1						
1	1	1	1	2	1				1	1	1						
1	1	1	1	1	1				4	1	1						
1	1	1	1	1	1				1	4	1						
2	1	1	1	1	1				1	1	1						
1	1	1	1	1	1				1	1	1						
1	1	1	1	1	1				2	3	2						
1	1	1	1	1	1				1	1	1						
1	1	1	1	1	1				1	1	1						
1	1	1	2	1	1				1	1	1						
1	1	1	1	1	1				1	1	1						
1	1	1	1	1	1				3	1	1						
1	1	1	1	1	1				1	1	1						
1	1	1	1	1	1				1	1	1						
1	1	1	1	1	2				1	1	2						
1	1	1	1	1	1				1	1	1						
1	1	1	1	1	1				1	1	1						
1	1	1	1	1	1				1	1	1						
1	1	1	1	1	1				1	1	1						
1	1	1	1	1	1				1	1	1						
1	1	1	1	1	1				1	1	1						
1	1	1	1	1	1				1	1	1						
1	1	1	1	1	1				1	1	1						
1	1	1	1	1	1				1	1	1						
1	1	1	1	1	1				1	1	1						
1	1	1	1	1	1				1	1	1						
1	1	1	1	1	1				1	1	2						
1	1	1	1	1	1				3	1	1						
1	1	1	1	1	3				3	1	1						
1	1	1	1	1	1				1	1	1						
1	1	1	1	2	1				1	1	1						
1	1	1	1	1	1				1	1	1						
1	1	1	1	1	1				1	5	1						
1	1	1	1	1	1				1	1	1						
1	1	1	1	1	1				1	1	1						
1	1	1	1	1	1				1	1	1						
1	1	1	1	1	1				1	1	1						
1	1	1	2	1	1				1	1	1						
1	1	1	1	1	1				1	1	1						
1	1	1	1	1	1				1	1	1						

Cluster magnitude

4 months Frequency (%)																		
	WT1	WT2	WT3	IC1	IC2	IC3	P1C1(1)	P1C2(1)	P1C3(1)	IPC1	IPC2	IPC3	P2C1	P2C2	P2C3	P1C1(2)	P1C2(2)	P1C3(2)
1	95.9596	96.633	97.6431	94.9495	96.633	96.2963	31.9588	36.0825	34.0206	89.899	92.2559	92.9293	32.9897	38.1443	32.9897	37.1134	38.1443	39.1753
2	3.367	3.0303	2.0202	3.7037	2.6936	2.6936	10.3093	11.3402	5.15464	4.0404	2.6936	3.367	13.4021	10.3093	10.3093	16.4948	12.3711	10.3093
3	0.6734	0.3367	0.3367	0.6734	0.3367	0.6734	15.4639	9.27835	14.433	3.0303	2.6936	2.0202	11.3402	14.433	10.3093	9.27835	8.24742	7.21649
4	0	0	0	0.3367	0.3367	0.3367	7.21649	9.27835	9.27835	2.0202	1.3468	1.0101	9.27835	3.09278	5.15464	5.15464	8.24742	9.27835
5	0	0	0	0.3367	0	0	2.06186	2.06186	3.09278	0.6734	1.0101	0.3367	6.18557	6.18557	6.18557	3.09278	6.18557	4.12371
6	0	0	0	0	0	0	5.15464	10.3093	7.21649	0.3367	0	0.3367	3.09278	3.09278	4.12371	4.12371	6.18557	4.12371
7	0	0	0	0	0	0	6.18557	5.15464	6.18557	0	0	0	3.09278	2.06186	1.03093	2.06186	2.06186	4.12371
8	0	0	0	0	0	0	0	4.12371	2.06186	0	0	0	6.18557	3.09278	4.12371	3.09278	4.12371	1.03093
9	0	0	0	0	0	0	2.06186	2.06186	4.12371	0	0	0	1.03093	2.06186	5.15464	0	2.06186	5.15464
10	0	0	0	0	0	0	1.03093	1.03093	2.06186	0	0	0	0	2.06186	2.06186	2.06186	1.03093	1.03093
11	0	0	0	0	0	0	2.06186	0	2.06186	0	0	0	3.09278	2.06186	2.06186	1.03093	0	2.06186
12	0	0	0	0	0	0	2.06186	0	2.06186	0	0	0	0	2.06186	2.06186	2.06186	0	1.03093
13	0	0	0	0	0	0	3.09278	0	0	0	0	0	0	1.03093	2.06186	1.03093	1.03093	1.03093
14	0	0	0	0	0	0	1.03093	0	0	0	0	0	1.03093	2.06186	1.03093	1.03093	0	1.03093
15	0	0	0	0	0	0	1.03093	1.03093	2.06186	0	0	0	1.03093	1.03093	0	1.03093	0	2.06186
16	0	0	0	0	0	0	3.09278	1.03093	0	0	0	0	3.09278	1.03093	1.03093	3.09278	0	1.03093
17	0	0	0	0	0	0	0	2.06186	1.03093	0	0	0	0	1.03093	1.03093	0	1.03093	0
18	0	0	0	0	0	0	0	1.03093	0	0	0	0	1.03093	0	1.03093	1.03093	2.06186	0
19	0	0	0	0	0	0	0	0	0	0	0	0	0	0	1.03093	1.03093	1.03093	2.06186
20	0	0	0	0	0	0	1.03093	0	1.03093	0	0	0	0	1.03093	1.03093	1.03093	0	0
21	0	0	0	0	0	0	0	0	1.03093	0	0	0	0	0	1.03093	2.06186	0	0
22	0	0	0	0	0	0	1.03093	1.03093	0	0	0	0	1.03093	0	0	0	0	0
23	0	0	0	0	0	0	2.06186	1.03093	0	0	0	0	0	1.03093	0	0	1.03093	0
24	0	0	0	0	0	0	0	0	1.03093	0	0	0	1.03093	0	0	0	1.03093	0
25	0	0	0	0	0	0	0	0	0	0	0	0	0	0	1.03093	0	1.03093	0
26	0	0	0	0	0	0	1.03093	0	0	0	0	0	0	0	0	0	0	2.06186
27	0	0	0	0	0	0	0	0	1.03093	0	0	0	0	0	0	1.03093	0	0
28	0	0	0	0	0	0	0	0	0	0	0	0	0	0	1.03093	1.03093	0	0
29	0	0	0	0	0	0	0	1.03093	0	0	0	0	0	1.03093	0	0	1.03093	0
30	0	0	0	0	0	0	0	1.03093	0	0	0	0	0	1.03093	1.03093	0	1.03093	1.03093
31	0	0	0	0	0	0	1.03093	0	0	0	0	0	0	0	0	0	0	0
32	0	0	0	0	0	0	0	0	0	0	0	0	0	0	2.06186	1.03093	0	0
33	0	0	0	0	0	0	0	0	0	0	0	0	0	0	0	0	1.03093	0
35	0	0	0	0	0	0	0	0	1.03093	0	0	0	0	0	0	0	0	0
36	0	0	0	0	0	0	0	0	0	0	0	0	1.03093	0	0	0	0	0
37	0	0	0	0	0	0	0	0	0	0	0	0	0	1.03093	0	0	0	1.03093
38	0	0	0	0	0	0	0	0	0	0	0	0	1.03093	0	0	0	0	0

Final data points of the plot

Cluster size	WT			IC			P1C(1)			IPC			P2C			P1C(2)		
	WT1	WT2	WT3	IC1	IC2	IC3	PC1	PC2	PC3	IPC1	IPC2	IPC3	P2C1	P2C2	P2C3	PC1	PC2	PC3
1	95.9596	96.633	97.6431	94.9495	96.633	96.2963	31.9588	36.0825	34.0206	89.899	92.2559	92.9293	32.9897	38.1443	32.9897	37.1134	38.1443	39.1753
2-4	4.0404	3.367	2.3569	4.7138	3.367	3.7037	32.9897	29.8969	28.866	9.09091	6.73401	6.39731	34.0206	27.8351	25.7732	30.9278	28.866	26.8041
>4	0	0	0	0.3367	0	0	35.0515	34.0206	37.1134	1.0101	1.0101	0.6734	32.9897	34.0206	41.2371	31.9588	32.9897	34.0206

	WT	IC	P1C(1)	IPC	P2C	P1C(2)
1	96.75±0.49%	95.96±0.51%	34.02±1.19%	91.69±0.92%	34.71±1.72%	38.14±0.6%
2-4	3.25±0.49%	3.93±0.4%	30.58±1.24%	7.41±0.85%	29.21±2.48%	28.87±1.19%
>4	0±0%	0.11±0.11%	35.4±0.91%	0.9±0.11%	36.08±2.59%	32.99±0.6%

Average clone size

WT	IC	P1C(1)	IPC	P2C	P1C(2)
1.04±0.01	1.06±0.01	5.24±0.36	1.17±0.02	5.55±0.41	5.3±0.4

1	1	1	1	1	1		1	1	1		
1	1	1	1	1	1		1	1	1		
1	1	1	1	1	1		2	1	1		
1	1	1	1	1	1		1	1	1		
1	1	1	1	1	1		1	1	5		
1	1	1	1	1	1		1	1	1		
1	1	1	1	1	1		1	1	1		
1	1	1	1	1	1		1	1	1		
1	1	1	1	1	1		1	2	1		
1	1	1	1	1	1		1	1	1		
1	1	1	1	1	3		1	1	1		
1	1	1	1	1	1		1	1	1		
1	1	1	1	2	1		1	1	1		
1	1	1	1	1	1		1	1	3		
1	1	1	1	1	1		4	1	1		
1	1	1	1	1	1		1	1	1		
1	1	1	1	1	1		1	1	1		
1	1	1	1	1	1		1	1	1		
1	1	1	1	1	1		1	1	1		
1	1	1	1	1	1		1	1	1		
1	1	1	1	1	1		1	1	1		
1	1	1	1	1	1		1	1	1		
1	1	1	1	1	1		1	1	1		
1	1	1	1	1	1		1	1	1		
1	1	1	2	1	1		1	3	1		
1	1	1	1	1	1		1	1	1		
1	1	1	1	1	1		1	1	1		
1	1	1	1	1	1		1	1	1		
1	1	1	1	1	1		1	1	1		
1	1	1	1	1	1		1	1	1		
1	1	1	1	1	1		1	1	1		
1	1	1	1	1	1		1	1	1		
1	1	1	1	1	1		1	1	1		
1	1	1	1	1	1		1	1	1		
1	1	1	1	1	1		1	1	1		
1	1	1	1	1	1		1	1	3		
1	1	1	1	1	4		1	1	1		
1	1	1	1	1	1		1	1	1		
1	1	1	1	1	1		1	1	1		
1	1	1	1	1	1		1	1	1		
1	1	1	1	1	1		1	1	1		
1	1	1	1	1	1		4	1	1		
1	1	1	1	1	1		1	1	1		
1	1	1	1	2	1		1	1	1		
1	1	1	1	1	1		1	3	1		
1	1	1	1	1	1		1	1	1		
1	1	1	1	1	1		1	1	1		
1	1	1	1	1	1		1	1	1		
1	1	1	1	1	1		3	1	1		
1	1	1	1	1	1		1	1	1		
1	1	1	1	1	1		1	1	1		
1	1	1	1	1	1		1	1	1		
1	1	1	1	1	1		1	2	1		
1	1	1	1	1	1		1	1	1		
1	1	1	1	1	1		1	1	1		
1	1	1	1	1	1		2	1	1		
1	1	1	2	1	1		1	1	1		
1	1	1	1	1	1		1	1	1		
1	1	1	1	1	1		1	1	1		
1	1	1	1	1	1		1	1	1		
1	1	1	1	1	1		1	1	1		
1	1	1	1	1	1		1	1	1		
1	1	1	1	1	1		1	1	1		
1	1	1	1	1	1		1	1	4		
1	1	1	1	1	1		1	1	1		
1	1	1	1	1	1		1	1	1		
1	1	1	1	1	1		1	1	1		
1	1	1	1	1	1		1	1	1		
1	1	1	1	1	1		1	1	1		
1	1	1	1	1	2		1	2	1		
1	1	1	1	1	1		1	1	1		
1	1	1	1	1	1		1	1	1		
1	1	1	1	1	1		1	1	1		
1	1	1	1	1	1		3	1	1		
1	1	1	1	1	1		1	1	1		
1	1	1	1	1	1		1	1	1		
1	1	1	1	1	1		1	1	1		
1	1	1	1	1	1		1	1	1		
1	1	1	1	1	1		1	1	1		
1	1	1	1	1	1		1	1	1		
1	1	1	1	1	1		1	1	1		
1	1	1	3	1	1		1	5	1		
1	1	1	1	1	1		1	1	1		
1	1	1	1	1	1		1	1	1		
1	1	1	1	1	1		1	1	1		
1	1	1	1	1	1		1	1	1		
1	1	1	1	1	1		1	1	1		
1	1	1	1	1	1		1	1	4		
1	1	1	1	1	1		1	1	1		
1	1	1	1	1	1		2	1	1		
1	1	1	1	1	1		1	1	1		
1	1	1	1	1	1		1	1	1		
1	1	1	1	2	1		1	1	1		
1	1	1	1	1	1		1	1	1		
1	1	1	1	1	1		1	1	1		
1	1	1	1	1	1		1	4	1		
1	1	1	1	1	1		1	1	1		
1	1	1	1	1	1		1	1	2		
1	1	1	1	1	1		1	1	1		
1	1	1	1	1	1		1	1	1		
1	1	1	1	1	1		3	1	1		
1	1	1	1	1	1		1	1	1		
1	1	1	1	2	1		1	1	1		

Figure 2H.3J

6 months																	
WT			IFT88f/f CAG			PKD1 f/f CAG(1)			IFT88f/f PKD1 f/f CAG			PKD2 f/f CAG			PKD1 f/f CAG(2)		
WT1	WT2	WT3	IC1	IC2	IC3	P1C1	P1C2	P1C3	IPC1	IPC2	IPC3	P2C1	P2C2	P2C3	P1C1	P1C2	P1C3
1	1	1	1	1	3	72	44	8	1	1	1	15	52	5	1	16	6
1	1	1	1	1	1	1	1	50	1	1	1	4	3	60	6	8	21
1	1	1	1	1	1	5	1	10	1	1	1	6	4	3	9	3	1
1	1	3	1	1	1	1	6	3	1	1	1	1	1	3	12	34	11
1	1	1	1	1	4	2	1	1	1	1	4	8	1	8	1	2	5
1	1	1	1	3	1	24	5	1	2	1	1	1	3	3	2	3	1
1	1	1	1	1	1	16	11	2	1	1	1	1	12	2	5	2	1
1	1	1	1	1	1	1	20	13	1	1	1	7	8	2	1	1	2
1	1	1	1	1	1	2	5	24	1	1	1	5	1	1	9	1	4
1	1	1	2	1	1	1	2	4	1	1	1	28	1	32	11	2	1
1	1	1	2	1	1	15	30	20	1	1	1	4	13	1	1	1	5
1	1	1	1	1	1	7	6	2	1	1	1	1	8	1	8	5	11
1	1	1	1	1	1	2	3	1	1	1	1	5	17	2	1	49	4
1	1	1	1	1	1	1	9	1	1	2	1	16	72	3	7	2	1
1	1	1	5	1	1	1	4	46	1	1	1	1	1	2	5	1	1
1	1	1	1	1	1	52	17	3	1	1	1	1	1	1	1	3	14
1	1	1	1	1	2	6	1	3	1	1	2	1	20	1	3	10	3
1	1	1	1	1	1	7	2	9	1	1	1	2	8	40	3	2	2
2	1	1	1	1	1	2	1	2	2	1	1	108	1	24	1	7	1
1	1	1	1	1	1	1	36	1	4	1	1	1	12	11	1	2	6
1	1	1	1	1	1	5	3	1	1	1	1	4	1	2	2	4	9
1	1	2	1	2	1	15	1	3	1	3	1	2	9	4	6	1	12
1	1	1	1	1	1	1	5	6	1	1	1	1	11	2	5	3	5
1	1	1	1	1	1	5	1	2	1	1	1	3	8	1	1	9	3
1	1	1	1	1	1	3	1	4	1	1	1	46	3	1	54	6	1
1	1	1	1	1	1	5	2	17	1	2	1	4	2	24	7	8	90
1	1	1	1	1	1	1	2	11	1	1	1	2	2	3	2	1	1
1	1	1	1	1	1	3	1	3	1	1	1	1	1	1	1	2	1
1	2	1	2	1	1	1	4	1	2	1	1	16	8	1	19	16	4
1	1	1	1	1	1	25	4	2	1	1	1	1	18	36	1	25	9
1	1	1	1	2	1	4	72	2	1	1	3	1	4	7	31	1	5
1	1	1	1	1	1	5	5	2	1	1	1	4	56	10	1	6	1
1	1	1	1	1	1	1	3	1	1	1	1	23	3	3	8	5	6
1	1	1	1	1	1	2	1	53	1	1	1	8	3	1	1	1	8
1	1	1	1	2	1	18	7	44	1	1	1	11	2	2	1	3	3
1	1	1	1	4	1	1	2	1	1	1	1	6	1	1	14	1	1
1	1	1	1	5	1	1	5	10	1	1	1	6	1	1	7	72	10
1	1	1	1	1	3	4	3	1	1	1	3	1	28	45	1	9	1
1	1	1	1	1	1	20	28	8	1	1	2	1	6	2	3	11	4
1	1	1	1	1	1	3	3	6	1	2	1	9	5	7	16	1	29
1	1	1	1	1	1	8	1	4	1	1	1	20	17	16	2	6	3
2	1	1	1	1	1	86	4	2	1	1	1	4	1	1	2	1	7
1	1	1	1	1	1	2	15	1	1	1	1	3	1	6	2	1	16
1	1	1	1	1	1	3	8	1	3	1	1	11	1	1	1	34	1
1	1	3	1	1	1	1	7	24	1	1	1	1	36	1	3	5	2
1	1	1	1	1	1	8	13	3	1	5	1	5	3	32	2	2	6
1	1	1	1	1	1	12	5	5	1	1	1	1	4	3	8	1	2
1	1	1	1	1	1	9	1	1	1	1	1	1	1	2	15	6	11
1	1	1	1	1	1	10	1	10	1	1	1	1	2	3	12	9	7
1	2	1	1	1	1	6	2	6	1	1	1	3	3	1	3	8	1
1	1	1	1	1	2	1	1	12	1	1	1	15	7	1	6	1	14
1	1	1	1	1	1	6	45	6	1	1	1	5	5	5	7	5	1
1	1	1	1	1	1	9	3	5	4	1	1	1	2	30	1	1	36
2	1	1	1	1	1	14	5	1	1	1	1	3	8	5	26	1	1
1	1	1	2	1	1	3	11	3	1	1	1	1	16	5	1	7	2
1	1	1	1	1	1	2	3	1	1	1	1	6	1	20	2	45	13
1	1	1	1	1	1	2	2	6	1	1	1	1	1	1	6	1	2
1	1	1	1	1	1	1	10	17	1	1	5	32	15	16	9	2	1
1	1	1	1	1	1	1	1	2	1	1	1	2	4	2	5	18	2
1	1	1	2	1	1	1	1	9	1	1	1	5	5	8	1	1	4
1	1	1	1	1	1	28	2	9	1	1	1	3	24	2	4	7	1
1	2	1	1	1	1	6	1	8	1	1	1	10	19	13	2	2	40
1	1	1	1	1	1	4	4	21	1	1	1	4	6	9	1	32	5
1	1	1	1	1	1	7	4	2	1	1	5	3	16	1	17	3	1
1	1	1	1	1	1	1	6	1	1	1	1	1	8	37	3	2	28
1	1	1	2	1	1	1	1	6	1	1	1	2	1	7	9	1	3
1	1	1	2	1	1	3	16	36	3	1	1	1	40	1	2	21	1
1	1	1	1	1	1	20	3	7	1	1	1	6	1	1	6	1	18
1	1	1	1	1	1	2	2	9	1	1	1	24	2	2	29	3	1
1	1	1	1	1	1	2	1	4	1	2	1	2	1	2	1	14	48
1	1	1	1	1	1	1	1	5	1	1	1	3	2	3	1	1	2
1	1	1	1	1	1	4	2	1	1	1	1	4	3	3	5	1	7
1	1	1	1	1	1	1	2	1	1	1	2	2	2	4	2	68	2
1	1	1	2	3	1	16	27	1	2	1	1	3	7	25	30	27	1
1	1	1	1	1	1	2	1	1	1	1	1	1	1	9	1	1	30
1	1	1	1	1	1	5	2	40	1	1	1	3	2	7	16	2	2
1	1	1	1	1	1	1	1	6	1	1	1	20	5	2	1	1	1
1	1	1	1	1	1	9	1	9	1	1	1	1	1	1	1	6	1
1	1	2	1	1	4	8	26	2	1	1	2	1	3	4	6	2	24
3	1	1	2	1	1	1	4	1	1	1	1	2	1	8	7	3	1
1	1	1	1	1	1	47	3	1	1	1	1	2	4	3	13	2	9
1	1	1	1	1	1	1	7	5	1	1	1	37	1	3	1	5	6
1	1	1	1	1	1	3	5	16	1	1	1	6	12	2	2	1	1
1	1	1	1	1	1	3	2	1	1	1	1	14	1	1	4	1	3
1	2	1	1	1	1	2	6	1	5	1	1	8	30	1	3	18	1
1	1	1	1	1	2	1	1	5	1	2	1	9	6	10	2	6	38
1	1	1	1	1	1	1	1	2	1	1	1	7	1	1	45	2	2
1	1	1	1	1	1	7	13	5	1	1	1	8	9	1	1	1	16
1	1	1	1	1	1	1	1	1	1	1	1	1	15	2	2	33	1
1	1	1	1	1	1	2	1	1	1	1	1	1	2	21	3	2	1
1	1	1	1	1	1	5	38	3	1	1	1	5	10	1	4	1	4
1	1	1	1	1	1	1	1	2	1	1	1	13	2	1	20	2	1
1	1	1	1	1	1	1	8	1	1	1	1	4	4	6	10	5	1
1	1	2	1	1	1	11	2	1	1	1	1	1	28	5	1	23	3
1	1	3	1	1	1	1	1	3	1	1	2	2	2	9	3	11	1
1	1	1	1	3	1	1	4	3	1	1	1	8	1	1	3	1	12
1	4	1	3	1	1	6	1	1	7	1	1	5	1	3	1	8	1
1	1	1	1	1	1				1	1	1						
1	1	1	1	1	1				1	1	1						
1	1	1	1	1	1				1	1	1						
1	1	1	1	1	1				1	1	1						
1	1	1	1	1	1				1	1	1						
1	1	1	1	1	1				1	1	1						
2	1	1	1	1	1				1	4	1						
1	1	1	1	1	1				1	1	1						
1	1	1	1	1	1				1	1	1						
1	1	2	1	1	1				1	1	1						
1	1	1	1	2	1				1	1	5						
1	1	1	1	1	1				1	1	1						
1	1	1	1	1	3				1	1	2						
1	1	1	1	1	1				1	1	1						
1	1	1	1	1	1				3	1	1						
1	1	1	1	1	1				1	1	1						
1	1	1	1	1	1				1	1	1						
1	1	1	1	1	1				1	1	1						
1	1	1	1	1	1				1	1	1						
1	1	1	1	1	1				1	1	1						
1	1	1	1	1	1				1	1	1						
1	1	1	1	1	1				1	1	1						
1	1	1	1	1	1				1	1	1						
1	1	1	1	1	1				1	1	1						
3	1	2	2	1	1				1	2	1						
1	1	1	1	1	1				1	1	1						
1	1	1	1	1	1				1	1	1						
1	1	1	1	1	1				1	1	1						
1	1	1	1	1	1				1	3	1						
1	1	1	1	1	1				1	2	1						
1	2	1	1	1	1				1	1	3						
1	1	1	1	1	1				2	1	1						
1	1	1	1	1	1				1	1	1						
1	1	1	1	1	1				1	1	1						
1	1	1	1	1	2				1	1	1						
1	1	1	1	1	1				1	1	1						
1	1	1	1	1	1				1	1	1						
1	1	1	1	1	1				1	1	1						
1	1	1	1	1	1				1	1	1						
1	1	1	1	1	1				1	1	1						
1	1	1	1	1	1				1	2	1						
1	1	1	1	1	1				1	1	1						
1	1	1	1	1	1				1	1	1						
2	1	1	1	1	1				1	1	1						
1	1	1	1	1	1				1	1	2						
1	1	1	1	1	1				4	1	1						
1	1	1	1	1	1				1	1	1						
1	1	1	1	1	1				1	1	1						
1	1	1	1	1	1				1	1	1						
1	1	1	1	1	1				1	1	1						
1	2	1	1	1	1				1	1	1						
1	1	1	1	1	1				1	1	1						
1	1	1	1	2	1				1	2	1						
1	1	1	1	1	1				1	1	1						
1	1	1	1	1	3				1	1	4						
1	1	1	1	1	1				1	1	1						
1	1	2	1	1	1				1	1	1						
1	1	1	1	1	1				1	1	1						
1	1	1	1	1	1				1	1	1						
1	1	1	1	1	1				6	1	1						
1	1	1	1	1	1				1	1	1						
1	1	1	1	1	1				1	1	1						
2	1	1	1	1	1				1	1	1						
1	1	1	1	1	1				1	1	1						
1	1	1	1	1	3				1	4	2						
1	1	1	3	1	1				1	1	1						
1	1	1	1	1	1				1	1	1						
1	1	1	1	1	1				2	1	1						
1	1	1	1	1	2				1	1	2						
1	3	1	1	1	1				1	1	1						
1	1	1	1	1	1				1	1	1						
1	1	1	1	1	1				1	1	1						
1	1	1	1	3	1				1	1	1						
1	1	1	1	1	1				1	1	1						
1	1	1	1	1	1				1	1	1						
1	1	1	1	1	1				1	1	1						
2	1	2	1	1	1				1	1	1						
1	1	1	2	1	1				1	1	1						
1	1	1	1	1	1				2	6	1						
1	1	1	1	1	1				1	1	1						
1	1	1	1	1	1				1	1	1						
1	1	1	1	1	1				1	1	1						
1	1	1	1	1	1				1	1	1						
1	1	1	2	1	1				1	1	3						
1	2	1	1	1	1				1	1	1						
1	1	1	1	1	1				1	1	1						
1	1	1	1	1	1				2	1	1						
1	1	1	1	1	1				1	1	1						
1	1	1	1	1	1				1	1	1						
1	1	1	1	1	1				1	1	1						
1	1	1	1	1	1				1	1	1						
1	1	1	1	1	1				1	1	1						
1	1	1	1	1	1				1	5	1						
1	1	2	1	1	1				1	1	4						
1	1	1	1	1	1				1	1	1						
1	1	1	1	2	1				1	1	1						

Cluster magnitude

6 months Frequency (%)																		
	WT1	WT2	WT3	IC1	IC2	IC3	P1C1(1)	P1C2(1)	P1C3(1)	IPC1	IPC2	IPC3	P2C1	P2C2	P2C3	P1C1(2)	P1C2(2)	P1C3(2)
1	95.9596	95.6229	95.2862	93.266	94.6128	93.9394	32.9897	30.9278	29.8969	91.5825	92.5926	91.2458	29.8969	28.866	29.8969	29.8969	29.8969	35.0515
2	3.0303	3.0303	3.367	4.3771	2.3569	2.0202	13.4021	14.433	13.4021	3.7037	4.0404	4.0404	10.3093	12.3711	16.4948	14.433	17.5258	11.3402
3	1.0101	1.0101	1.3468	1.0101	2.0202	2.3569	8.24742	9.27835	10.3093	2.0202	0.6734	2.0202	9.27835	9.27835	13.4021	10.3093	8.24742	7.21649
4	0	0.3367	0	0.3367	0.3367	1.3468	4.12371	8.24742	4.12371	1.0101	1.0101	1.3468	9.27835	6.18557	3.09278	3.09278	1.03093	6.18557
5	0	0	0	1.0101	0.6734	0	7.21649	8.24742	6.18557	1.0101	1.0101	1.3468	7.21649	4.12371	5.15464	5.15464	6.18557	5.15464
6	0	0	0	0	0	0.3367	5.15464	4.12371	7.21649	0.3367	0.6734	0	6.18557	3.09278	2.06186	6.18557	6.18557	5.15464
7	0	0	0	0	0	0	4.12371	3.09278	1.03093	0.3367	0	0	2.06186	2.06186	4.12371	5.15464	3.09278	3.09278
8	0	0	0	0	0	0	3.09278	2.06186	3.09278	0	0	0	5.15464	7.21649	3.09278	3.09278	4.12371	1.03093
9	0	0	0	0	0	0	3.09278	1.03093	5.15464	0	0	0	2.06186	2.06186	3.09278	4.12371	3.09278	3.09278
10	0	0	0	0	0	0	1.03093	1.03093	3.09278	0	0	0	1.03093	1.03093	2.06186	1.03093	1.03093	1.03093
11	0	0	0	0	0	0	1.03093	2.06186	1.03093	0	0	0	2.06186	1.03093	1.03093	1.03093	2.06186	3.09278
12	0	0	0	0	0	0	1.03093	0	1.03093	0	0	0	0	3.09278	0	2.06186	0	2.06186
13	0	0	0	0	0	0	0	2.06186	1.03093	0	0	0	1.03093	1.03093	1.03093	1.03093	0	1.03093
14	0	0	0	0	0	0	1.03093	0	0	0	0	0	1.03093	0	0	1.03093	1.03093	2.06186
15	0	0	0	0	0	0	2.06186	1.03093	0	0	0	0	2.06186	2.06186	0	1.03093	0	0
16	0	0	0	0	0	0	2.06186	1.03093	1.03093	0	0	0	2.06186	2.06186	2.06186	2.06186	2.06186	2.06186
17	0	0	0	0	0	0	0	1.03093	2.06186	0	0	0	0	2.06186	0	1.03093	0	0
18	0	0	0	0	0	0	1.03093	0	0	0	0	0	0	1.03093	0	0	2.06186	1.03093
19	0	0	0	0	0	0	0	0	0	0	0	0	0	1.03093	0	1.03093	0	0
20	0	0	0	0	0	0	2.06186	1.03093	1.03093	0	0	0	2.06186	1.03093	1.03093	1.03093	0	0
21	0	0	0	0	0	0	0	0	1.03093	0	0	0	0	0	1.03093	0	1.03093	1.03093
22	0	0	0	0	0	0	0	0	0	0	0	0	0	0	0	0	0	0
23	0	0	0	0	0	0	0	0	0	0	0	0	1.03093	0	0	0	1.03093	0
24	0	0	0	0	0	0	1.03093	0	2.06186	0	0	0	1.03093	1.03093	2.06186	0	0	1.03093
25	0	0	0	0	0	0	1.03093	0	0	0	0	0	0	0	1.03093	0	1.03093	0
26	0	0	0	0	0	0	0	1.03093	0	0	0	0	0	0	0	1.03093	0	0
27	0	0	0	0	0	0	0	1.03093	0	0	0	0	0	0	0	0	1.03093	0
28	0	0	0	0	0	0	1.03093	1.03093	0	0	0	0	1.03093	2.06186	0	0	0	1.03093
29	0	0	0	0	0	0	0	0	0	0	0	0	0	0	0	1.03093	0	1.03093
30	0	0	0	0	0	0	0	1.03093	0	0	0	0	0	1.03093	1.03093	1.03093	0	1.03093
31	0	0	0	0	0	0	0	0	0	0	0	0	0	0	0	1.03093	0	0
32	0	0	0	0	0	0	0	0	0	0	0	0	1.03093	0	2.06186	0	1.03093	0
33	0	0	0	0	0	0	0	0	0	0	0	0	0	0	0	0	1.03093	0
34	0	0	0	0	0	0	0	0	0	0	0	0	0	0	0	0	2.06186	0
36	0	0	0	0	0	0	0	1.03093	1.03093	0	0	0	0	1.03093	1.03093	0	0	1.03093
37	0	0	0	0	0	0	0	0	0	0	0	0	1.03093	0	1.03093	0	0	0
38	0	0	0	0	0	0	0	1.03093	0	0	0	0	0	0	0	0	0	1.03093
40	0	0	0	0	0	0	0	0	1.03093	0	0	0	0	1.03093	1.03093	0	0	1.03093
44	0	0	0	0	0	0	0	1.03093	1.03093	0	0	0	0	0	0	0	0	0
45	0	0	0	0	0	0	0	1.03093	0	0	0	0	0	0	1.03093	1.03093	1.03093	0
46	0	0	0	0	0	0	0	0	1.03093	0	0	0	1.03093	0	0	0	0	0
47	0	0	0	0	0	0	1.03093	0	0	0	0	0	0	0	0	0	0	0
48	0	0	0	0	0	0	0	0	0	0	0	0	0	0	0	0	0	1.03093
49	0	0	0	0	0	0	0	0	0	0	0	0	0	0	0	0	1.03093	0
50	0	0	0	0	0	0	0	0	1.03093	0	0	0	0	0	0	0	0	0
52	0	0	0	0	0	0	1.03093	0	0	0	0	0	0	1.03093	0	0	0	0
53	0	0	0	0	0	0	0	0	1.03093	0	0	0	0	0	0	0	0	0
54	0	0	0	0	0	0	0	0	0	0	0	0	0	0	0	1.03093	0	0
56	0	0	0	0	0	0	0	0	0	0	0	0	0	1.03093	0	0	0	0
60	0	0	0	0	0	0	0	0	0	0	0	0	0	0	1.03093	0	0	0
68	0	0	0	0	0	0	0	0	0	0	0	0	0	0	0	0	1.03093	0
72	0	0	0	0	0	0	1.03093	1.03093	0	0	0	0	0	1.03093	0	0	1.03093	0
74	0	0	0	0	0	0	0	0	0	0	0	0	0	0	0	0	0	0
86	0	0	0	0	0	0	1.03093	0	0	0	0	0	0	0	0	0	0	0
90	0	0	0	0	0	0	0	0	0	0	0	0	0	0	0	0	0	1.03093
108	0	0	0	0	0	0	0	0	0	0	0	0	1.03093	0	0	0	0	0

Final data points of the plot

Cluster size	WT			IC			P1C(1)			IPC			P2C			P1C(2)		
	WT1	WT2	WT3	IC1	IC2	IC3	PC1	PC2	PC3	IPC1	IPC2	IPC3	P2C1	P2C2	P2C3	PC1	PC2	PC3
1	95.9596	95.6229	95.2862	93.266	94.6128	93.9394	32.9897	30.9278	29.8969	91.5825	92.5926	91.2458	29.8969	28.866	29.8969	29.8969	29.8969	35.0515
2-4	4.0404	4.3771	4.7138	5.72391	4.7138	5.72391	25.7732	31.9588	27.8351	6.73401	5.72391	7.40741	28.866	27.8351	32.9897	27.8351	26.8041	24.7423
>4	0	0	0	1.0101	0.6734	0.3367	41.2371	37.1134	42.268	1.6835	1.6835	1.3468	41.2371	43.299	37.1134	42.268	43.299	40.2062

	WT	IC	P1C(1)	IPC	P2C	P1C(2)
1	95.62±0.19%	93.94±0.39%	31.27±0.91%	91.81±0.4%	29.55±0.34%	31.62±1.72%
2-4	4.38±0.19%	5.39±0.34%	28.52±1.82%	6.62±0.49%	29.9±1.57%	26.46±0.91%
>4	0±0%	0.67±0.19%	40.21±1.57%	1.57±0.11%	40.55±1.82%	41.92±0.91%

Average clone size

WT	IC	P1C(1)	IPC	P2C	P1C(2)
1.06±0.01	1.11±0.02	7.44±0.71	1.17±0.02	7.77±0.72	7.6±0.7

1	1	1	1	1	1		5	1	1		
1	1	1	1	1	1		1	1	1		
1	1	1	1	1	1		1	1	1		
1	1	1	1	1	1		1	1	1		
1	1	2	1	1	1		1	1	1		
1	1	1	1	1	4		1	1	1		
1	1	1	1	1	1		1	1	1		
1	1	1	1	1	1		1	1	1		
1	1	1	1	1	1		1	1	1		
1	1	1	1	5	1		1	1	1		
1	1	1	1	1	1		1	1	1		
2	1	1	3	1	1		1	1	1		
1	1	1	1	1	1		2	1	1		
1	1	1	1	1	1		1	1	3		
1	1	1	1	1	1		1	4	1		
1	1	1	1	1	1		1	1	1		
1	1	1	1	1	1		1	1	1		
1	1	1	1	1	1		1	1	1		
1	1	1	1	1	3		1	1	1		
1	1	1	1	1	1		3	1	1		
1	1	1	1	1	1		1	1	1		
1	1	1	1	1	1		1	1	1		
1	2	1	1	1	1		1	1	1		
1	1	1	1	1	1		1	1	1		
1	1	1	1	1	1		1	1	1		
1	1	1	1	1	1		1	1	1		
1	1	1	1	1	1		1	5	1		
1	1	1	1	1	1		1	1	1		
1	1	1	1	1	1		1	1	1		
1	3	1	1	1	1		1	1	1		
1	1	1	1	1	1		1	1	1		
1	1	1	1	1	1		1	1	5		
1	1	1	1	3	1		1	1	1		
1	1	1	1	1	1		3	1	1		
1	1	1	1	1	1		1	1	1		
1	1	1	1	1	1		1	1	1		
1	1	1	2	1	1		1	1	1		
1	1	1	1	1	1		1	1	1		
1	1	1	1	1	1		1	1	1		
1	1	1	1	1	1		1	1	1		
1	1	1	1	1	1		1	1	1		
1	1	1	1	1	1		1	1	1		
1	1	1	1	1	1		1	1	2		
1	1	1	1	1	1		1	1	1		
1	1	1	1	1	1		1	1	1		
1	1	1	1	1	1		1	1	1		
1	1	1	1	1	4		1	1	1		
1	3	1	1	1	1		2	1	1		
1	1	1	1	1	1		1	1	2		
1	1	1	1	1	1		1	1	1		
1	1	1	1	1	1		1	1	1		
1	1	1	1	1	1		1	1	1		
1	1	1	1	1	1		1	2	1		
1	1	1	1	1	1		1	1	1		
1	1	1	1	1	1		1	1	1		
1	1	1	1	1	1		1	1	1		
1	1	1	1	1	1		1	1	1		
1	1	1	1	1	1		1	1	3		
1	1	1	1	1	1		1	1	1		
1	1	1	1	1	6		1	1	1		
2	1	1	1	1	1		1	1	1		
1	1	1	1	1	1		1	1	1		
1	1	1	1	1	1		1	1	1		
1	1	1	1	1	1		1	1	1		
1	1	1	1	1	1		2	1	1		
1	1	1	1	1	1		1	1	1		
1	1	3	4	1	1		1	1	1		
1	1	1	1	1	1		1	1	1		
1	1	1	1	1	1		1	1	1		
1	1	1	1	1	1		1	1	1		
1	1	1	1	1	1		1	1	1		
1	1	1	1	1	1		1	1	1		
1	1	1	1	1	1		1	6	1		
1	1	1	1	3	1		1	1	1		
1	2	1	1	1	1		1	1	1		
1	1	1	1	1	1		1	1	1		
1	1	1	1	1	1		1	1	1		
1	1	1	1	1	1		1	1	2		
1	1	1	1	1	1		1	1	1		
1	1	1	1	1	1		1	1	1		
1	1	1	1	1	1		5	1	1		
1	1	1	1	1	1		1	1	1		
1	1	1	1	1	1		1	1	1		
1	1	1	1	1	1		1	1	1		
1	1	1	1	1	1		1	1	1		
1	1	1	1	1	1		1	1	1		
1	1	1	1	1	2		1	1	1		
1	1	1	1	1	1		1	1	1		
1	1	1	1	1	1		1	1	1		
1	1	1	1	1	1		1	1	1		
1	1	1	5	1	1		1	1	4		
3	1	1	1	1	1		1	2	1		
1	1	1	1	1	1		1	1	1		
1	1	1	1	1	1		1	1	1		
1	1	2	1	2	1		1	2	1		
1	1	1	1	1	1		1	1	1		
1	1	1	1	1	1		1	1	1		
1	1	1	1	1	1		1	1	1		
1	1	1	1	1	3		1	1	1		
1	1	1	1	1	1		1	1	1		
1	1	1	1	1	1		1	1	1		
1	1	1	5	1	1		3	1	1		
1	1	1	1	1	1		1	1	1		
1	1	1	1	1	1		1	1	1		

Figure 4F,4G,4H

2 months						4 months						6 months					
WT			CK19 PKD1			WT			CK19 PKD1			WT			CK19 PKD1		
WT1	WT2	WT3	CP1	CP2	CP3	WT1	WT2	WT3	CP1	CP2	CP3	WT1	WT2	WT3	CP1	CP2	CP3
1	1	1	1	1	1	1	1	1	1	11	3	1	2	1	1	35	15
1	1	1	1	1	1	1	1	1	33	10	1	2	1	1	34	24	17
1	1	1	1	1	1	1	1	1	1	3	1	1	1	1	1	42	1
1	2	1	1	2	1	1	1	1	2	1	1	1	1	1	1	4	40
1	1	1	1	1	1	1	1	1	1	25	1	1	1	2	1	1	1
1	1	1	3	1	1	1	1	1	1	4	1	2	1	1	15	1	1
1	1	1	1	1	1	1	1	1	1	1	1	1	1	1	1	28	21
1	1	1	1	1	1	1	1	1	1	1	1	1	1	1	1	1	1
1	1	1	1	1	15	1	1	1	1	1	22	1	2	1	26	1	2
1	1	1	1	1	1	1	2	1	2	1	1	1	1	1	1	17	1
1	1	1	2	1	1	1	1	1	10	1	1	3	1	1	1	1	1
1	1	2	1	1	1	1	1	1	2	1	1	1	1	1	1	1	7
2	1	1	1	1	1	1	1	1	1	21	2	1	1	1	48	3	2
1	1	1	1	2	1	1	1	2	22	1	1	1	1	2	25	1	9
1	1	1	1	1	1	1	1	1	6	1	3	1	1	1	1	1	2
1	1	1	3	1	1	1	1	1	1	1	1	1	1	1	16	1	1
1	1	1	1	1	2	1	1	1	1	1	12	1	2	1	12	24	1
1	1	1	1	1	1	1	1	1	1	1	3	1	1	1	1	5	1
1	1	1	1	1	1	1	1	1	1	4	1	1	1	1	1	8	12
1	1	1	1	3	1	2	1	1	7	1	8	1	1	1	1	7	36
1	1	1	1	1	1	1	1	1	1	2	2	1	1	1	23	4	16
1	2	1	2	1	1	1	1	1	6	5	1	1	1	1	1	1	44
1	1	1	1	1	1	1	1	1	1	1	1	1	1	1	1	1	1
1	1	1	1	1	1	1	1	1	1	1	1	1	4	1	2	10	1
1	1	1	1	3	1	1	1	1	1	1	20	1	1	1	3	1	1
1	1	1	1	1	1	1	1	1	1	8	1	1	1	1	5	4	1
1	1	1	5	1	1	3	1	1	1	4	1	1	1	1	1	1	4
1	1	1	1	1	1	1	1	1	1	1	1	1	1	1	1	12	1
1	1	1	1	1	1	1	1	1	5	1	2	1	1	1	1	1	1
1	1	1	1	1	1	1	1	1	1	1	1	1	1	1	6	7	1
1	1	1	1	1	1	1	1	1	6	2	1	1	2	1	1	1	8
1	1	1	6	1	1	1	1	1	1	4	1	1	1	1	1	5	3
1	1	1	1	1	1	1	1	1	1	1	7	1	1	1	1	1	1
1	1	1	1	1	1	1	1	2	1	1	4	1	1	1	16	4	1
1	1	1	1	1	1	1	1	1	1	1	1	1	1	2	23	1	16
1	1	2	1	10	1	1	1	1	1	1	1	1	1	1	2	1	1
1	1	1	1	1	1	1	1	1	1	9	1	2	1	1	1	13	1
1	1	1	1	1	1	1	2	1	1	1	2	1	1	1	1	1	9
1	1	1	1	1	1	1	1	1	14	1	2	1	2	1	1	2	20
1	1	1	3	1	1	1	1	1	1	11	1	1	1	1	1	48	1
2	1	1	1	1	1	1	1	1	1	1	1	1	1	1	17	1	1
1	1	1	1	1	1	1	1	1	2	1	6	1	1	1	9	1	1
1	1	1	1	1	1	1	1	1	4	1	1	1	1	1	46	1	2
1	1	1	1	1	1	2	1	1	1	5	1	1	1	1	1	1	1
1	1	1	1	1	1	1	1	1	1	1	1	4	1	1	13	1	1
1	1	1	1	1	8	1	1	2	1	10	5	1	1	1	16	21	29
1	1	1	1	1	1	1	1	1	7	2	1	1	1	1	8	34	1
1	1	1	1	1	1	1	1	1	13	2	4	1	1	1	7	1	4
1	1	1	1	1	1	1	2	1	1	3	2	1	1	1	14	1	16
1	1	1	1	1	1	1	1	1	1	15	29	1	1	3	4	23	1
1	1	1	1	1	1	1	1	1	1	1	2	1	1	1	3	1	1
1	1	1	1	1	1	1	1	1	2	1	8	1	1	1	1	1	1
1	1	1	1	2	1	1	1	1	6	1	4	1	1	1	1	1	6
1	1	1	1	1	1	1	1	1	9	1	1	1	1	1	1	1	1
1	2	1	1	1	1	1	1	1	1	28	11	1	1	1	1	5	5
1	1	1	1	1	1	1	1	1	1	1	8	1	1	1	16	7	11
1	1	1	1	1	1	1	1	1	1	2	5	1	1	1	1	12	1
1	1	1	3	1	1	1	1	1	17	3	1	1	2	1	1	1	46
1	1	1	1	1	1	1	1	1	1	1	1	1	1	1	37	16	3
1	1	1	1	1	1	2	1	1	1	1	2	1	1	1	29	37	3
1	1	1	1	1	1	1	1	1	1	7	3	1	1	1	5	11	1
1	1	1	1	1	5	1	1	1	26	19	1	1	1	1	1	2	2
1	1	1	1	1	1	1	1	1	2	1	1	1	1	1	1	8	33
1	1	1	1	1	1	1	1	2	6	1	21	1	1	1	2	1	1
1	1	1	1	1	1	1	1	1	1	1	17	1	1	2	12	4	8
1	1	1	1	1	1	1	1	1	1	36	1	1	1	1	4	1	20
1	1	1	1	1	1	1	1	1	10	9	1	1	1	1	9	1	1
1	1	1	1	1	1	1	1	1	1	1	5	1	1	1	1	25	1
1	1	1	1	1	1	1	1	1	1	1	1	1	1	1	1	1	18
1	1	1	1	1	1	1	1	1	24	1	1	1	1	1	6	1	2
2	1	1	1	1	1	1	1	1	1	1	9	1	1	1	1	1	1
1	1	1	1	1	1	1	1	1	3	12	1	1	1	1	1	10	3
1	1	1	1	6	1	1	1	1	6	10	1	1	1	1	1	17	4
1	1	1	1	1	1	1	1	1	1	2	1	1	1	2	1	1	11
1	1	1	1	1	1	1	2	1	1	1	7	1	1	1	1	1	49
1	1	1	1	1	2	1	1	1	1	1	1	1	1	1	12	1	1
1	1	1	8	1	1	1	1	1				2	1	1			
1	1	1	1	1	1	1	1	1				1	1	1			
1	1	1	1	1	1	1	1	1				1	1	1			
1	1	1	1	1	1	1	1	1				1	1	1			
1	1	1	1	1	1	1	1	1				1	1	1			
1	1	1	1	1	1	1	1	1				1	1	1			
1	1	1	2	1	1	1	1	1				1	1	1			
1	1	1	2	1	1	1	1	1				1	1	1			
1	1	1	1	1	1	1	1	2				1	1	1			
1	1	1	1	1	1	1	1	1				1	2	1			
1	1	2	1	1	1	1	1	1				1	1	1			
1	1	1	1	1	1	1	1	1				1	1	1			
1	1	1	1	1	4	1	1	1				1	1	1			
1	1	1	1	1	1	1	1	1				1	1	1			
1	1	1	1	1	1	1	1	1				1	1	1			
1	1	1	1	1	1	1	1	1				1	3	1			
1	1	1	1	7	1	1	1	1				1	1	1			
1	1	1	1	1	1	1	3	1				1	1	1			
1	2	1	1	1	1	1	1	1				2	1	1			
3	1	1	1	1	1	1	1	1				1	1	1			
1	1	1	1	1	1	1	1	1				1	1	1			
1	1	1	1	1	3	1	1	1				1	1	1			
1	1	1	1	1	1	1	1	2				1	1	1			
1	1	1	1	2	1	1	1	1				1	1	1			
1	1	1	1	1	1	1	1	1				1	1	1			
1	1	1	14	1	1	1	1	1				1	1	1			
1	1	1	1	1	1	1	1	1				1	1	2			
1	1	1	1	1	2	1	1	1				1	1	1			
1	1	1	1	2	1	1	1	1				1	1	1			
1	1	1	1	1	1	1	1	1				1	1	1			
1	1	1	1	1	1	1	1	1				1	1	1			
1	1	1	1	1	1	1	1	1				1	1	1			
1	1	1	1	1	2	1	1	1				1	1	1			
1	1	1	1	1	1	1	1	1				1	1	1			
1	1	1	1	1	1	1	1	1				1	2	1			
1	1	1	3	5	1	1	3	1				1	1	1			
1	2	1	1	1	1	1	1	1				1	1	1			
1	1	1	1	1	1	1	1	1				1	1	1			
1	1	1	1	1	1	1	1	1				1	1	1			
1	1	2	1	1	1	1	1	1				1	2	1			

Cluster magnitude

2 months Frequency(%)

	WT1	WT2	WT3	CP1	CP2	CP3
1	96.8	96	96.8	88.42975	90.90909	92.56198
2	2.4	4	3.2	3.305785	4.132231	3.305785
3	0.8	0	0	4.132231	1.652893	0.826446
4	0	0	0	0.826446	0	0.826446
5	0	0	0	0.826446	0.826446	0.826446
6	0	0	0	0.826446	0.826446	0
7	0	0	0	0	0.826446	0
8	0	0	0	0.826446	0	0.826446
9	0	0	0	0	0	0
10	0	0	0	0	0.826446	0
11	0	0	0	0	0	0
12	0	0	0	0	0	0
13	0	0	0	0	0	0
14	0	0	0	0.826446	0	0
15	0	0	0	0	0	0.826446

Final data points of the plot

Cluster size	WT			CK19 PKD1		
	WT1	WT2	WT3	CP1	CP2	CP3
1	96.8	96	96.8	88.42975	90.90909	92.56198
2-4	3.2	4	3.2	8.264463	5.785124	4.958678
>4	0	0	0	3.305785	3.305785	2.479339

	WT	CP
1	96.53±0.27%	90.63±1.2%
2-4	3.47±0.27%	6.34±0.99%
>4	0±0%	3.03±0.28%

Average clone size

WT	CP
1.04±0.01	1.31±0.07

Cluster magnitude

4 months Frequency(%)

	WT1	WT2	WT3	CP1	CP2	CP3
1	96.05263	94.73684	94.73684	64.47368	59.21053	57.89474
2	3.289474	3.947368	5.263158	7.894737	7.894737	10.52632
3	0.657895	1.315789	0	1.315789	3.947368	5.263158
4	0	0	0	1.315789	5.263158	3.947368
5	0	0	0	1.315789	2.631579	3.947368
6	0	0	0	7.894737	0	1.315789
7	0	0	0	2.631579	1.315789	2.631579
8	0	0	0	0	1.315789	3.947368
9	0	0	0	1.315789	2.631579	1.315789
10	0	0	0	2.631579	3.947368	0
11	0	0	0	0	2.631579	1.315789
12	0	0	0	0	1.315789	1.315789
13	0	0	0	1.315789	0	0
14	0	0	0	1.315789	0	0
15	0	0	0	0	1.315789	0
16	0	0	0	0	0	0
17	0	0	0	1.315789	0	1.315789
18	0	0	0	0	0	0
19	0	0	0	0	1.315789	0
20	0	0	0	0	0	1.315789
21	0	0	0	0	1.315789	1.315789
22	0	0	0	1.315789	0	1.315789
23	0	0	0	0	0	0
24	0	0	0	1.315789	0	0
25	0	0	0	0	1.315789	0
26	0	0	0	1.315789	0	0
27	0	0	0	0	0	0
28	0	0	0	0	1.315789	0
29	0	0	0	0	0	1.315789
30	0	0	0	0	0	0
33	0	0	0	1.315789	0	0
36	0	0	0	0	1.315789	0

Final data points of the plot

Cluster size	WT			CK19 PKD1		
	WT1	WT2	WT3	CP1	CP2	CP3
1	96.05263	94.73684	94.73684	64.47368	59.21053	57.89474
2-4	3.947368	5.263158	5.263158	10.52632	17.10526	19.73684
>4	0	0	0	25	23.68421	22.36842

	WT	CP
1	95.18±0.44%	60.53±2.01%
2-4	4.82±0.44%	15.79±2.74%
>4	0±0%	23.68±0.76%

Average clone size

WT	CP
1.05±0.01	4.03±0.41

Cluster magnitude

6 months Frequency (%)

	WT1	WT2	WT3	CP1	CP2	CP3
1	93.79845	91.47287	93.79845	53.94737	52.63158	48.68421
2	4.651163	6.976744	5.426357	3.947368	2.631579	7.894737
3	0.775194	0.775194	0.775194	2.631579	1.315789	5.263158
4	0.775194	0.775194	0	2.631579	6.578947	3.947368
5	0	0	0	2.631579	3.947368	1.315789
6	0	0	0	2.631579	0	1.315789
7	0	0	0	1.315789	3.947368	1.315789
8	0	0	0	1.315789	2.631579	2.631579
9	0	0	0	2.631579	0	2.631579
10	0	0	0	0	2.631579	0
11	0	0	0	0	1.315789	2.631579
12	0	0	0	3.947368	2.631579	1.315789
13	0	0	0	1.315789	1.315789	0
14	0	0	0	1.315789	0	0
15	0	0	0	1.315789	0	1.315789
16	0	0	0	5.263158	1.315789	3.947368
17	0	0	0	1.315789	2.631579	1.315789
18	0	0	0	0	0	1.315789
19	0	0	0	0	0	0
20	0	0	0	0	0	2.631579
21	0	0	0	0	1.315789	1.315789
22	0	0	0	0	0	0
23	0	0	0	2.631579	1.315789	0
24	0	0	0	0	2.631579	0
25	0	0	0	1.315789	1.315789	0
26	0	0	0	1.315789	0	0
27	0	0	0	0	0	0
28	0	0	0	0	1.315789	0
29	0	0	0	1.315789	0	1.315789
30	0	0	0	0	0	0
31	0	0	0	0	0	0
32	0	0	0	0	0	0

1	1	1	1	1	1	2	1	1	1	1	1
1	1	1	1	1	1	1	1	2	1	1	1
1	1	1	1	1	1	1	1	1	1	1	1
1	1	1	1	1	1	1	1	1	1	1	1
1	1	1	4	1	1	1	1	1	1	1	1
1	1	1				1	1	1	1	1	1
1	1	1				1	1	1	1	1	1
1	1	1				1	1	1	1	1	2
1	1	1				1	2	1	1	1	1
						1	1	1	1	1	1
						1	1	1	2	1	1
						1	1	1	1	1	1
						1	1	1	1	1	1
						1	1	1			
						1	1	1			
						1	1	1			
						1	1	2			
						1	1	1			
						1	1	1			
						1	1	1			
						1	1	1			
						2	1	1			
						1	1	1			
						1	1	1			
						1	1	1			
						1	1	1			
						1	1	1			
						1	1	1			
						1	1	1			
						1	1	1			
						1	1	1			
						1	1	1			
						1	2	1			
						1	1	1			
						1	1	1			
						1	1	1			

33	0	0	0	0	0	1.315789
34	0	0	0	1.315789	1.315789	0
35	0	0	0	0	1.315789	0
36	0	0	0	0	0	1.315789
37	0	0	0	1.315789	1.315789	0
38	0	0	0	0	0	0
39	0	0	0	0	0	0
40	0	0	0	0	0	1.315789
42	0	0	0	0	1.315789	0
44	0	0	0	0	0	1.315789
46	0	0	0	1.315789	0	1.315789
48	0	0	0	1.315789	1.315789	0
49	0	0	0	0	0	1.315789

Final data points of the plot

Cluster size	WT			CK19 PKD1		
	WT1	WT2	WT3	CP1	CP2	CP3
1	93.79845	91.47287	93.79845	53.94737	52.63158	48.68421
2-4	6.20155	8.527132	6.20155	9.210526	10.52632	17.10526
>4	0	0	0	36.84211	36.84211	34.21053

	WT	CP
1	93.02±0.78%	51.75±1.58%
2-4	6.98±0.78%	12.28±1.73%
>4	0±0%	35.96±0.51%

Average clone size

WT	CP
1.09±0.02	7.63±0.74

Figure 1A

11 days		
CAG^{creER} ; Confetti$^{fl/wt}$		
WT1	WT2	WT3
1	1	1
1	1	1
1	1	1
1	1	1
1	1	1
1	1	1
1	1	1
1	1	1
1	1	1
1	1	1
1	1	1
1	1	1
1	1	1
1	1	1
1	1	1
1	1	1
1	1	1
1	1	1
1	1	1
1	1	1
1	1	1
1	1	1
1	1	1
1	1	1
1	1	1
1	1	1
1	1	1
1	1	1
1	1	1
1	1	1
1	1	1
1	1	1
1	1	1
1	1	2
1	1	1
1	1	1
2	1	1
1	1	1
1	1	1
1	1	1
1	1	1
1	1	1
1	1	1
1	1	1
1	1	1
1	1	1
1	1	1
1	1	1
1	1	1
1	1	1
1	1	1
1	1	1
1	1	1
1	1	1
1	1	1
1	1	1
1	1	1
1	1	1
1	2	1
1	1	1
1	1	1
1	1	1
1	1	1
1	1	1
1	1	1
1	1	1
1	1	1
1	1	1
1	1	1
1	1	1
1	1	1
1	1	1
1	1	1
1	1	1
1	1	1
1	1	1
1	1	1
1	1	1
1	1	1
1	1	1
1	1	1
1	1	1
1	1	1
1	1	1
1	1	1
1	1	1
1	1	1
2	1	1
1	1	1
1	1	1
1	1	1
1	1	1
1	1	1
1	1	1
1	1	1
1	1	1
1	1	1
1	1	1
1	1	1
1	1	1
1	1	1
1	1	1
1	2	1
1	1	1
1	1	1
1	1	1
1	1	1
1	1	1
1	1	1
1	1	1
1	1	1
1	1	1
1	1	1
1	1	1
1	1	1
1	1	1
1	1	1
1	1	1

Figure 2A

32 days		
CAG^{creER} ; Confetti$^{fl/wt}$		
WT1	WT2	WT3
1	1	1
1	1	1
1	1	1
1	1	1
1	1	1
1	1	1
2	1	1
1	1	1
1	1	1
1	1	1
1	1	1
1	1	1
1	1	1
1	1	1
1	1	1
1	1	1
1	1	2
1	1	1
1	1	1
1	1	1
1	1	1
1	1	1
1	1	1
1	1	1
1	1	1
1	1	1
1	1	1
1	1	1
1	1	1
1	1	1
2	1	1
1	1	1
1	1	1
1	1	1
1	1	1
1	1	1
1	1	1
1	1	1
1	1	1
1	1	1
1	1	1
1	1	1
1	1	1
1	1	1
1	1	1
1	2	1
1	1	1
1	1	1
1	1	1
1	1	1
1	1	1
1	1	3
1	1	1
1	1	1
1	1	1
1	1	1
1	1	1
1	1	1
1	1	1
1	1	1
1	1	1
1	1	1
1	1	1
1	1	1
1	1	1
1	1	1
1	1	1
1	1	1
1	1	1
1	1	1
1	1	1
1	1	1
1	1	1
1	3	1
1	1	1
1	1	1
1	1	1
1	1	1
1	1	1
1	1	1
1	1	1
1	1	1
1	1	1
1	1	1
1	1	1
1	1	1
1	1	1
1	1	1
1	1	1
1	1	1
1	1	1
1	1	1
1	1	2
1	1	1
1	1	1
1	1	1
1	1	1
1	1	1
1	1	1
1	1	1
1	1	1
1	1	1
1	1	1
1	1	1
1	1	1
1	1	1
1	1	1
1	1	1
1	1	1
1	1	1
1	1	1
1	1	1
1	1	1
1	1	1
1	1	1
1	1	1
1	1	1
1	1	1

Figure 4A

32 days		
CK19^{creER} ; Confetti$^{fl/wt}$		
WT1	WT2	WT3
1	1	1
1	1	1
1	1	1
1	1	2
1	1	1
1	1	1
1	1	1
1	1	1
1	1	1
1	1	1
1	1	1
1	1	1
1	1	1
1	1	1
1	1	1
1	1	1
1	1	1
1	1	1
1	1	1
1	1	1
1	1	1
1	2	1
1	1	1
1	1	1
1	1	1
1	1	1
1	1	1
1	1	1
2	1	1
1	1	1
1	1	1
1	1	1
1	1	1
1	1	1
1	1	1
1	1	1
1	1	1
1	1	1
1	1	1
1	1	1
1	1	1
1	1	2
1	1	1
1	1	1
1	1	1
1	1	1
1	1	1
1	1	1
1	1	1
1	1	1
1	1	1
1	1	1
1	1	1
2	1	1
1	1	1
1	1	1
1	1	1
1	1	1
1	1	1
1	1	1
1	1	1
1	1	1
1	1	1
1	1	1
1	2	1
1	1	1
1	1	1
1	1	1
1	1	1
1	1	1
1	1	1
1	1	1
1	1	1
1	1	1
1	1	1
1	1	1
1	1	1
1	1	1
1	1	1
1	1	1
1	1	1
1	1	1
1	1	1
1	1	1
1	1	1
1	1	1
1	1	1
1	1	2
1	1	1
1	1	1
1	1	1
2	1	1
1	1	1
1	1	1
1	1	1
1	1	1
1	1	1

11 days Frequency(%)

Cluster magnitude	WT1	WT2	WT3
1	98.316498	98.989899	98.653199
2	1.3468013	1.010101	1.3468013
3	0.3367003	0	0

Final data points of the plot

	WT
1	98.65±0.19%
2-4	0.67±0.26%

Average clone size

WT
1.01±0

32 days Frequency(%)

Cluster magnitude	WT1	WT2	WT3
1	97.979798	98.316498	97.643098
2	1.6835017	1.3468013	2.020202
3	0.3367003	0.3367003	0.3367003

Final data points of the plot

	WT
1	97.98±0.19%
2-4	1.01±0.31%

Average clone size

WT
1.02±0.01

32 days Frequency(%) liver

Cluster magnitude	WT1	WT2	WT3
1	96.907216	97.938144	96.907216
2	3.0927835	2.0618557	3.0927835
3	0	0	0

Final data points of the plot

	WT
1	97.25±0.34%
2-4	1.37±0.63%

Average clone size

WT
1.03±0.01

1	1	1	1	1	1
1	1	1	3	1	1
1	1	1	1	1	2
1	1	1	1	1	1
1	1	1	1	1	1
1	1	1	1	1	1
1	1	1	1	1	1
1	1	1	1	1	1
1	1	1	1	1	1
1	1	1	1	1	1
1	1	1	1	1	1
1	1	1	1	1	1
1	1	1	1	1	1
1	1	2	1	1	1
1	1	1	1	1	1
1	1	1	1	1	1
1	1	1	1	1	1
1	1	1	1	1	1
1	1	1	1	1	1
1	1	1	1	1	1
1	1	1	1	1	1
1	1	1	2	1	1
1	1	1	1	1	1
1	1	1	1	1	1
1	1	1	1	1	1
1	1	1	1	1	1
1	1	1	1	1	1
1	1	1	1	1	1
1	1	1	1	1	1
1	1	1	1	1	1
1	1	1	1	1	1
1	1	1	1	1	1
2	1	1	1	1	1
1	1	1	1	2	1
1	1	1	1	1	1
1	1	1	1	1	1
1	1	1	1	1	1
1	1	1	1	1	1
1	1	1	1	1	1
1	1	1	1	1	1
1	1	1	1	1	1
1	1	1	1	1	1
1	1	1	1	1	1
1	1	1	1	1	1
1	1	1	1	1	1
1	1	1	1	1	1
1	1	1	1	1	1
1	1	1	1	1	1
1	1	1	1	1	1
1	1	1	1	1	1
1	1	1	1	1	1
1	1	1	1	1	2
1	1	1	1	1	1
1	1	1	1	1	1
1	1	1	1	1	1
1	1	1	1	1	1
1	1	1	1	1	1
1	1	1	1	1	1
1	1	1	1	1	1
1	1	1	1	1	1
1	1	1	1	1	1
1	1	1	1	1	1
1	2	1	1	1	1
1	1	1	1	1	1
1	1	1	1	1	1
1	1	1	1	1	1
1	1	1	1	1	1
1	1	1	1	1	1
1	1	1	1	1	1
1	1	1	1	1	1
1	1	1	1	1	1
1	1	1	1	1	2
1	1	1	1	1	1
1	1	1	1	1	1
1	1	1	1	1	1
1	1	1	1	1	1
1	1	1	1	1	1
1	1	1	1	1	1
1	1	1	1	1	1
1	1	1	1	1	1
1	1	1	1	1	1
1	1	1	1	1	1
1	1	1	1	1	1
1	1	1	1	1	1
1	1	1	1	1	1
1	1	1	1	1	1
2	1	1	1	1	1
1	1	1	1	1	1
1	1	1	1	1	1
1	1	1	1	1	1
1	1	1	1	1	1
1	1	1	1	1	1
1	1	1	1	1	1
1	1	1	1	1	1
1	1	1	1	1	1
1	1	1	1	1	1
1	1	1	1	1	1
1	1	1	1	1	1
1	1	1	1	2	1
1	1	1	1	1	1
1	1	1	1	1	1
1	1	1	1	1	1
1	1	1	1	1	1
1	1	1	1	1	1
1	1	1	1	1	1
1	1	1	1	1	1
1	1	1	1	1	1
1	1	1	1	1	1
1	1	1	1	1	1
1	1	1	1	1	1
1	1	2	1	1	1
1	1	1	1	1	1
1	1	1	1	1	1
1	1	1	1	1	1
1	1	1	1	1	1
1	1	1	1	1	1
1	1	1	1	1	1
1	1	1	1	1	1
1	1	1	1	1	1
1	1	1	1	1	1
1	1	1	1	1	1
1	1	1	1	1	1

1	1	1
1	1	1
1	1	1
1	1	1
1	1	1
1	1	1
1	1	1
1	1	1
1	1	1
1	1	1
1	1	1
1	1	1
1	1	1
1	1	1
1	1	1
1	1	1
1	1	1
1	1	1
1	1	1
1	1	1
1	1	1
1	1	1
1	1	1
1	1	1
1	1	1
1	1	1
1	1	1
1	1	1
1	1	1
1	1	1
1	1	1
1	1	1
1	1	1
1	1	1
1	1	1
1	1	1
1	1	1
1	1	1
3	1	1
1	1	1
1	1	1
1	1	1
1	1	1
1	1	1
1	1	1
1	1	1
1	1	1
1	1	1
1	1	2
1	1	1
1	1	1
1	1	1
1	1	1
1	1	1
1	1	1
1	1	1
1	1	1

1	1	1
1	1	1
1	1	1
1	1	1
1	1	1
1	1	1
1	1	1
1	1	1
2	1	1
1	1	1
1	1	1
1	1	1
1	1	1
1	1	1
1	1	1
1	1	1
1	1	1
1	1	1
1	1	1
1	1	1
1	1	1
1	1	1
1	1	1
1	1	2
1	1	1
1	1	1
1	1	1
1	1	1
1	1	1
1	1	1
1	1	1
1	1	1
1	1	1
1	1	1
1	1	1
1	1	1
1	2	1
1	1	1
1	1	1
1	1	1
1	1	1
1	1	1
1	1	1
1	1	1
1	1	1
1	1	1
1	1	1
1	1	1
1	1	1
1	1	1
1	1	1
1	1	1
2	1	1
1	1	1
1	1	1
1	1	1
1	1	1
