# Supplementary material for: Epithelial Dynamics of Cystogenesis in Genetic Models of Autosomal Dominant Polycystic Kidney Disease
Source: Cells. 2026 Feb 4;15(3):297. doi: 10.3390/cells15030297 (PMC12896517; doi:10.3390/cells15030297)
Supplement: Supplementary file 1 [file cells-15-00297-s001.zip › Table S2.pdf]

Supplementary Table S2

Tabular presentation of oligonucleotide sequences

**Genomic target**	**Orientation**	**Sequence**
Pkd2-gRNA	gRNA1	TGTATTACCAGGGTATCCACAGG
	gRNA2	GCAGCGACATCACAGTCTATGGG
$Pkd2^{fl}$ genotype	Forward (F1)	TGCTTGTCATTAGAACCCACAGT
	Reverse (R1)	CTCTGCATTTACTGACATGGAATGG
$Pkd2^{fl}$ genotype	Forward (F2)	CTCCACTTCTGTGGGTAGAACTTG
	Reverse (R2)	TCCGACATAACCAAACAGACTGA
$Pkd1^{fl}$ genotype	Forward (F1)	GTGGTGCTTGTAGCTACCTGAT
	Reverse (R1)	CTTCCTTCTCAGTGGATAATC
$Pkd1^{fl}$ genotype	Forward (F2)	GAGCAGGTACTCCGCCAGTTC
	Reverse (R2)	GATTCTCATAGGTATTGGACAC
CAG^{CreER} genotype	forward	GCTAACCATGTTCATGCCTTC
	reverse	AGGCAAATTTTGGTGTACGG
$Ck19^{CreER}$ genotype	forward	CCAGAATCCCCACGAATTGACC
	reverse	GTTCTTGCGAACCTCATCACTC
$Ift88^{fl}$ genotype	forward	GACCACCTTTTTAGCCTCCTG
	reverse	AGGGAAGGGACTTAGGAATGA
$Rosa^{Brainbow}$ genotype	forward	GAATTAATTCCGGTATAACTTCG
	reverse	AAAGTCGCTCTGAGTTGTTAT CCAGATGACTACCTATCCTC
