# Supplementary figures and images for: Epithelial Dynamics of Cystogenesis in Genetic Models of Autosomal Dominant Polycystic Kidney Disease
Source: Cells. 2026 Feb 4;15(3):297. doi: 10.3390/cells15030297 (PMC12896517; doi:10.3390/cells15030297)

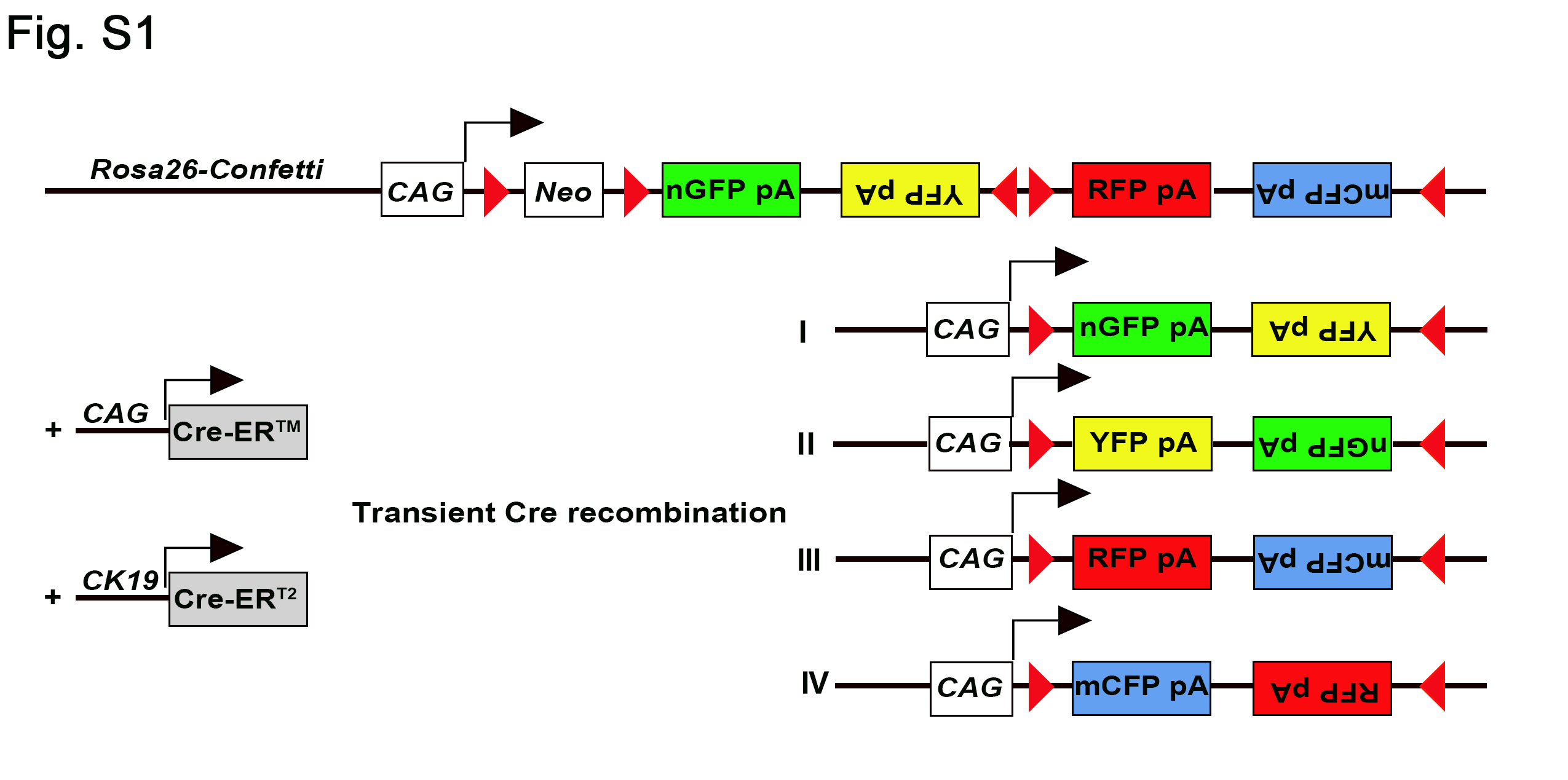

Supplement: Supplementary file 1 [file cells-15-00297-s001.zip › Supplemental Figure S1.tif]

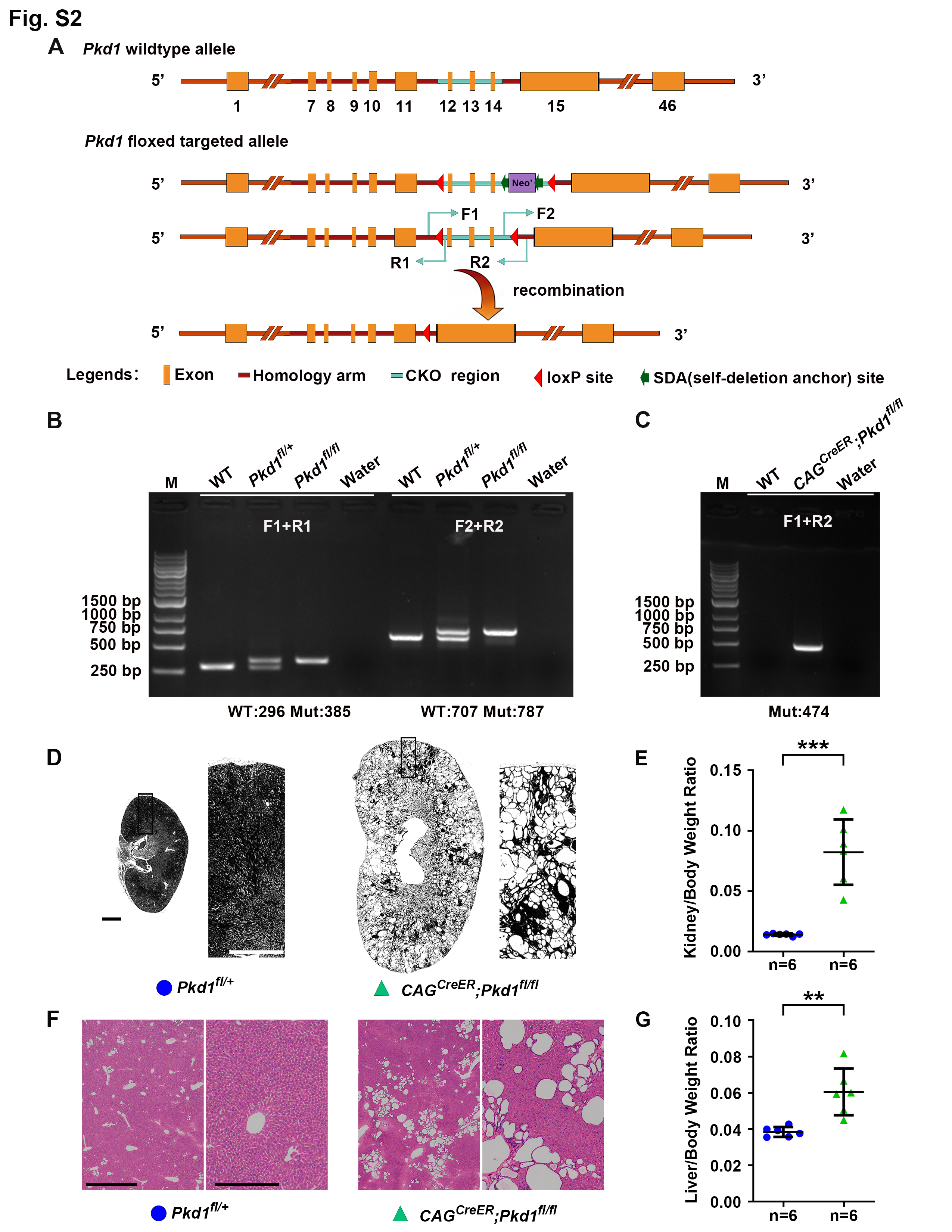

Supplement: Supplementary file 1 [file cells-15-00297-s001.zip › Supplemental Figure S2.tif]

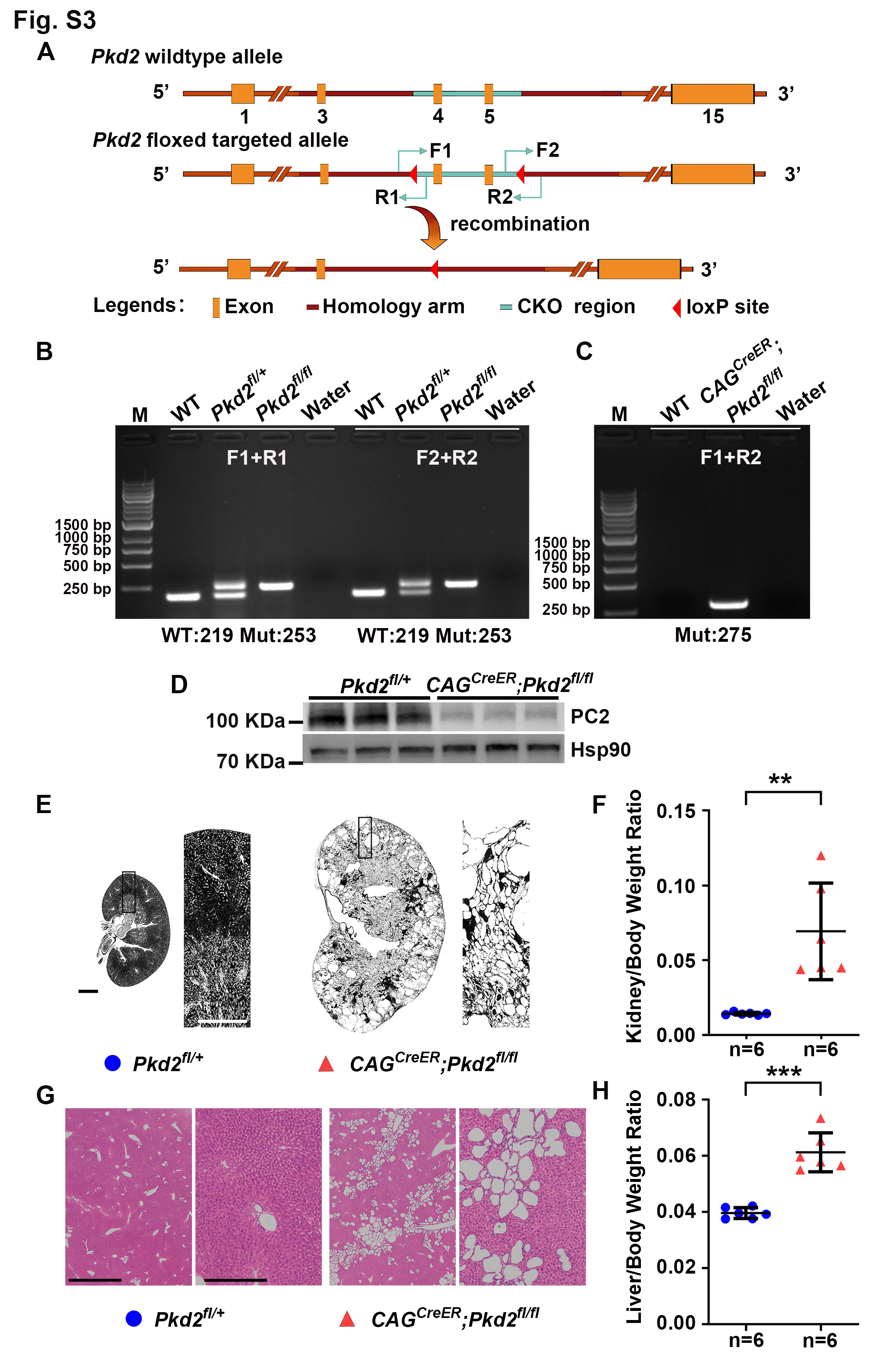

Supplement: Supplementary file 1 [file cells-15-00297-s001.zip › Supplemental Figure S3.tif]

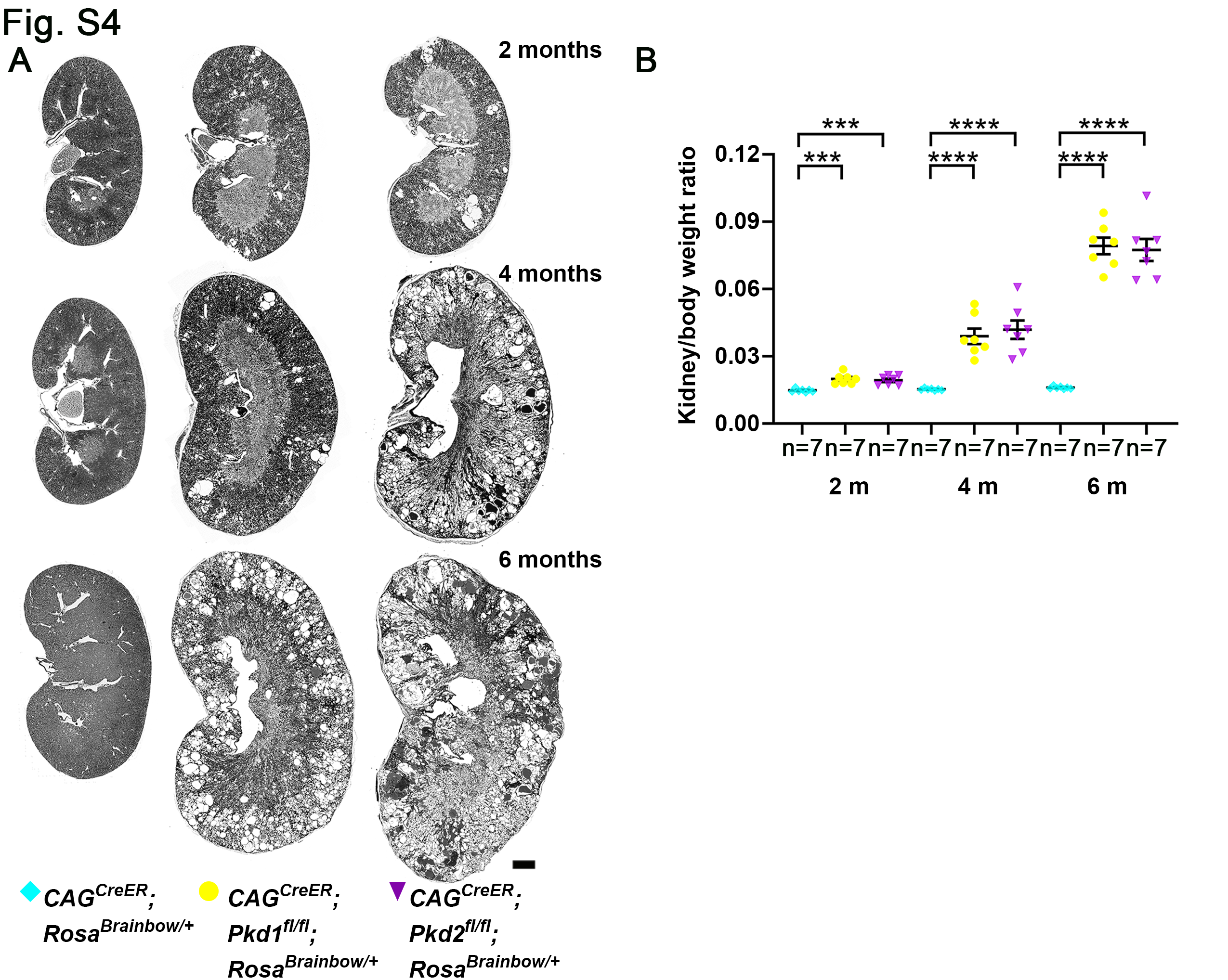

Supplement: Supplementary file 1 [file cells-15-00297-s001.zip › Supplemental Figure S4.tif]

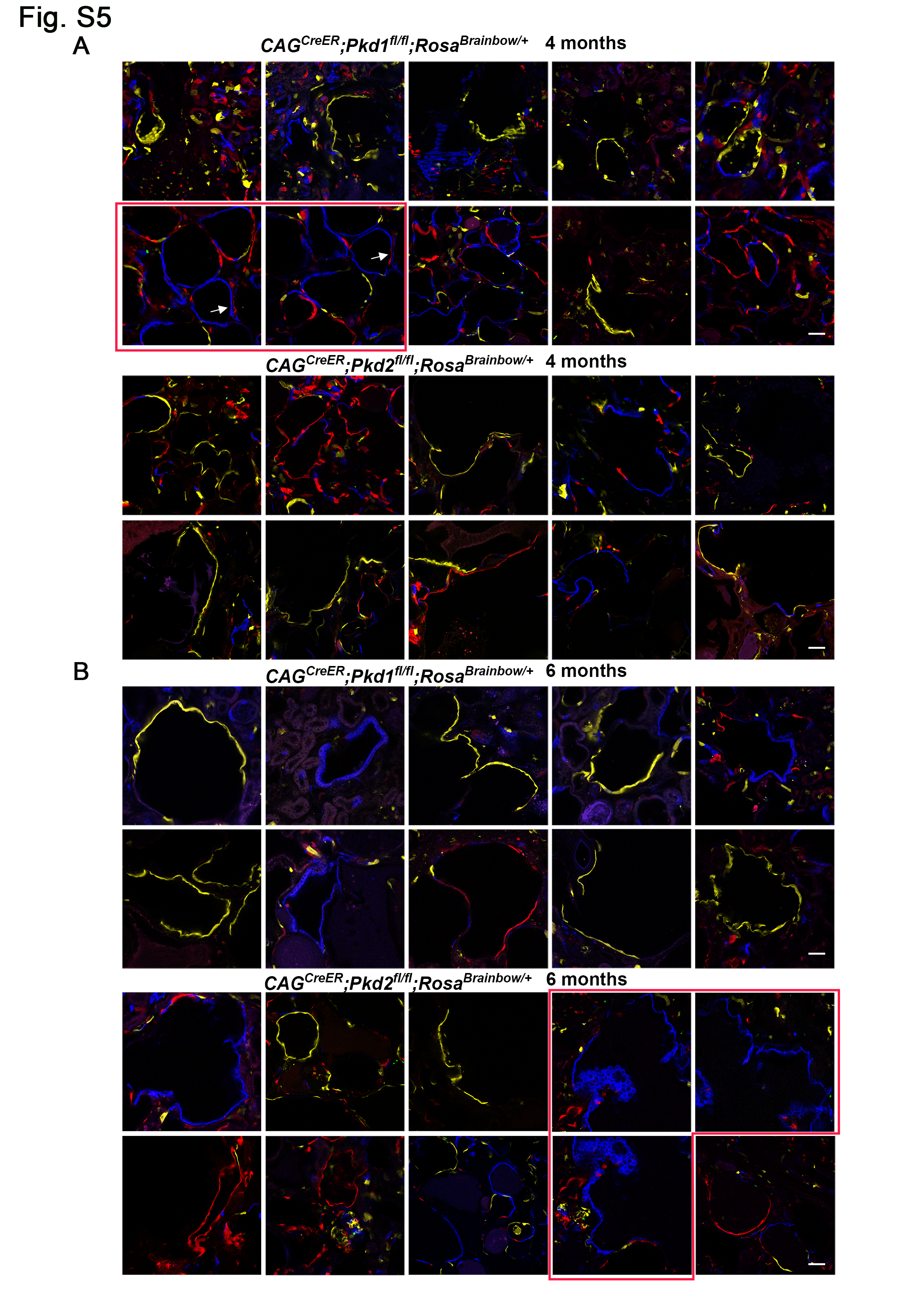

Supplement: Supplementary file 1 [file cells-15-00297-s001.zip › Supplemental Figure S5.tif]

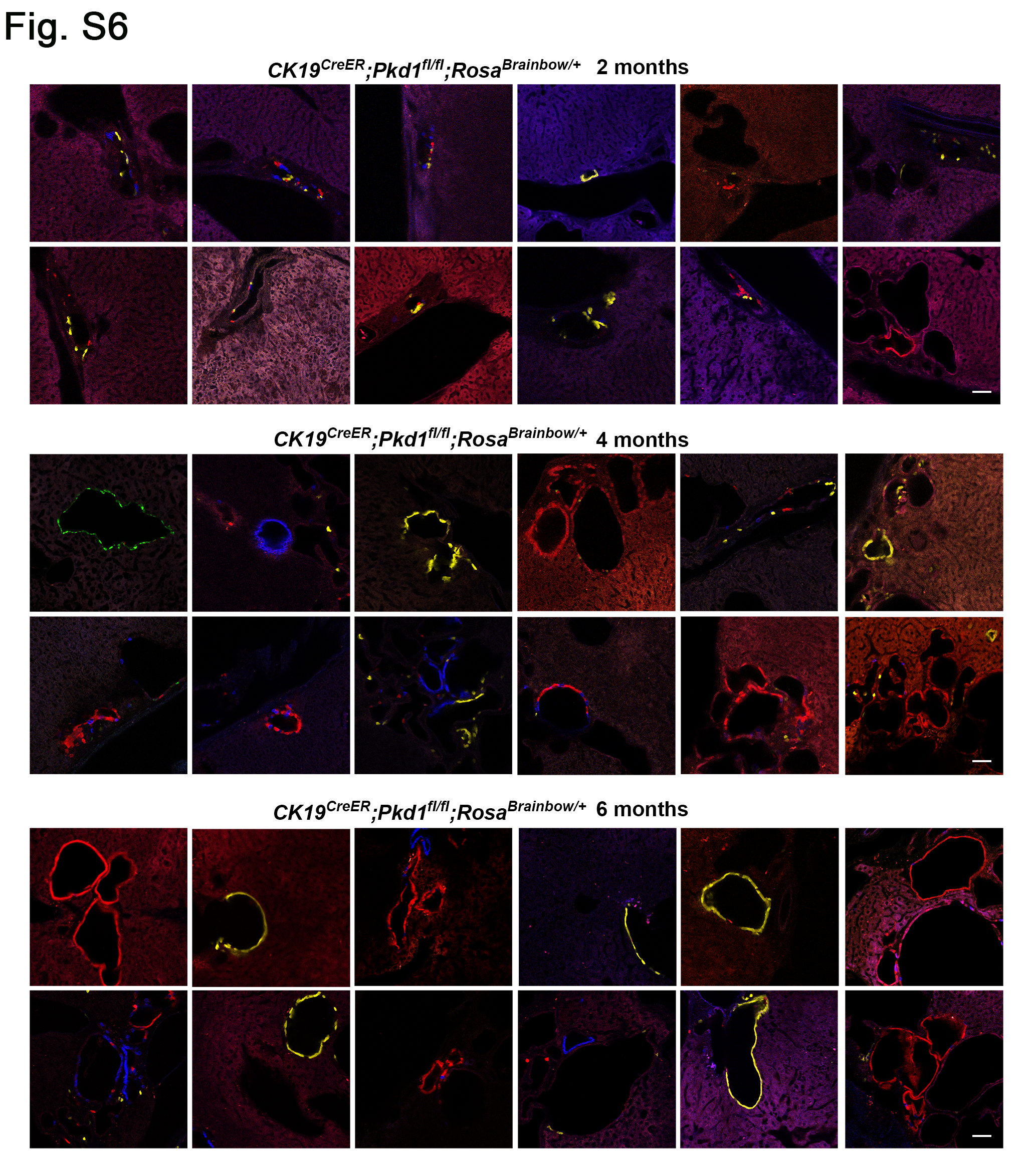

Supplement: Supplementary file 1 [file cells-15-00297-s001.zip › Supplemental Figure S6.tif]
